# Development of Selective ADAMTS-5 Peptide Substrates to Monitor Proteinase Activity

**DOI:** 10.1021/acs.jmedchem.2c02090

**Published:** 2023-02-22

**Authors:** Milan M. Fowkes, Linda Troeberg, Paul E. Brennan, Tonia L. Vincent, Morten Meldal, Ngee H. Lim

**Affiliations:** † Centre for OA Pathogenesis Versus Arthritis, Kennedy Institute of Rheumatology, University of Oxford, Roosevelt Drive, Headington, Oxford OX3 7FY, United Kingdom; ‡ Norwich Medical School, Bob Champion Research and Education Building, Rosalind Franklin Road, University of East Anglia, Norwich NR4 7UQ, United Kingdom; § Alzheimer’s Research UK Oxford Drug Discovery Institute, Centre for Medicines Discovery, Nuffield Department of Medicine Research Building, University of Oxford, Old Road Campus, Headington, Oxford OX3 7FZ, United Kingdom; ∥ Department of Chemistry, University of Copenhagen, Universitetsparken 5, Building B304, Copenhagen DK-2100, Denmark

## Abstract

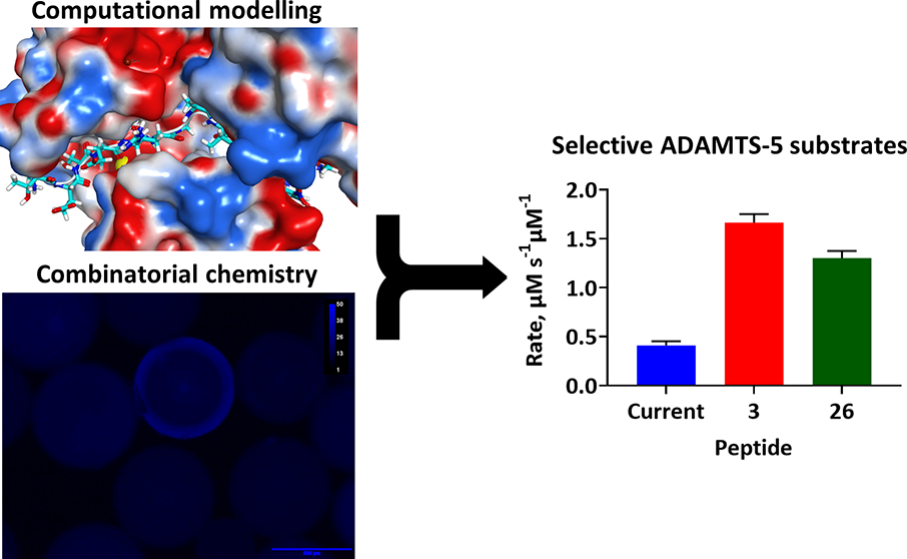

The dysregulation of proteinase activity is a hallmark of osteoarthritis (OA), a disease characterized by progressive degradation of articular cartilage by catabolic proteinases such as a disintegrin and metalloproteinase with thrombospondin type I motifs-5 (ADAMTS-5). The ability to detect such activity sensitively would aid disease diagnosis and the evaluation of targeted therapies. Förster resonance energy transfer (FRET) peptide substrates can detect and monitor disease-related proteinase activity. To date, FRET probes for detecting ADAMTS-5 activity are nonselective and relatively insensitive. We describe the development of rapidly cleaved and highly selective ADAMTS-5 FRET peptide substrates through *in silico* docking and combinatorial chemistry. The lead substrates **3** and **26** showed higher overall cleavage rates (∼3–4-fold) and catalytic efficiencies (∼1.5–2-fold) compared to the best current ADAMTS-5 substrate *ortho*-aminobenzoyl(Abz)-TESE↓SRGAIY-*N*-3-[2,4-dinitrophenyl]-l-2,3-diaminopropionyl(Dpa)-KK-NH_2_. They exhibited high selectivity for ADAMTS-5 over ADAMTS-4 (∼13–16-fold), MMP-2 (∼8–10-fold), and MMP-9 (∼548–2561-fold) and detected low nanomolar concentrations of ADAMTS-5.

## Introduction

The degradation of articular cartilage is a major pathological feature of osteoarthritis (OA), a joint disease affecting more than 300 million people worldwide.^1,2^ The extracellular matrix of cartilage consists of two major structural components: type II collagen and aggrecan. OA is thought to be characterized by the initial degradation of aggrecan by proteinases of the a disintegrin and metalloproteinase with thrombospondin type I motifs (ADAMTS) family. More specifically, ADAMTS-5 has been identified as the main aggrecanase in surgical mouse models of osteoarthritis,^3,4^ whereas both ADAMTS-4 and ADAMTS-5 have been implicated in human disease progression.^5−7^ Aggrecan degradation is likely followed by the breakdown of type II collagen by members of the matrix metalloproteinase family (MMP-1, MMP-8, and MMP-13).^8−15^ The temporal nature of specific proteinase activity in cartilage degradation is supported by *in vitro* experiments with bovine cartilage explants treated with interleukin-1, in which aggrecan degradation occurs in the first week of culture and type II collagen breakdown thereafter.^8−10^ Additionally, in both murine and human osteoarthritic cartilage explants, loss of aggrecan staining is observed earlier than loss of type II collagen staining.^11,12^

X-ray radiography is currently used to monitor the degradation of cartilage in OA.^16,17^ Due to the radiolucency of cartilage, this degradation is inferred as a narrowing of the joint space between the two ends of bone, making radiography an indirect and insensitive measure of cartilage loss.^16,17^ As radiography is performed in 2D, there are also limitations associated with understanding the 3D pattern of wear across the entire joint surface. Consequently, there is an urgent need to develop more sensitive biomarkers to detect early osteoarthritis and monitor disease progression, either before or after therapeutic interventions.

**Figure 1:**
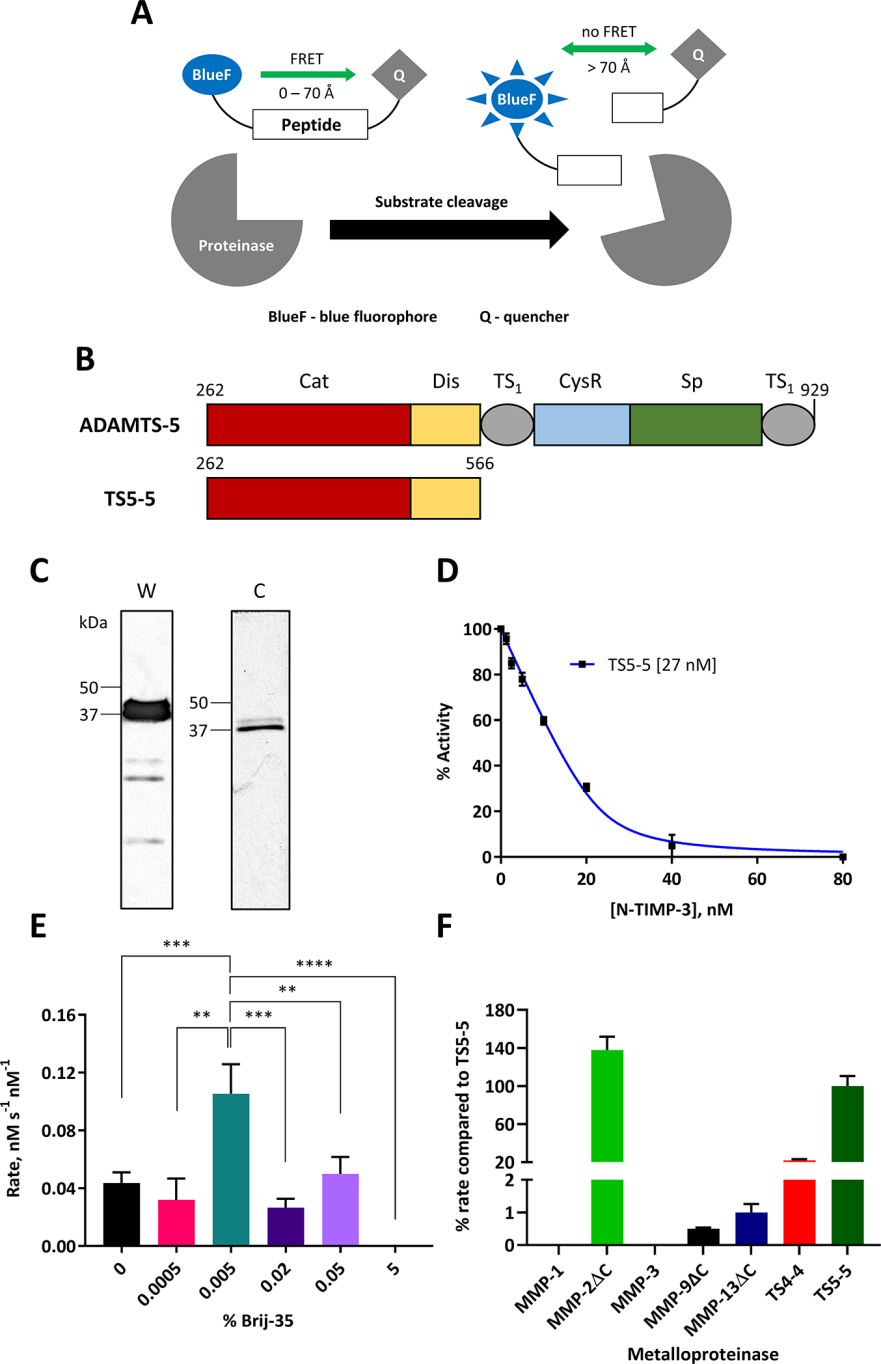
The current ADAMTS-5 FRET peptide substrate lacks selectivity. (A) Proteinase activity can be measured by the increase in signal intensity resulting from cleavage of FRET peptide substrates. (B) Schematic representation of the domain organization of full-length ADAMTS-5 and the TS5-5 domain deletion mutant used here. Domains and cutoffs are defined according to Gendron et al.^28^ (C) Representative Western blot and Coomassie stain of purified TS5-5. (D) Determination of TS5-5 proteinase concentration through active site titration with different concentrations of N-TIMP-3. The current FRET substrate Abz-TESE↓SRGAIY-Dpa-KK-NH_2_ (20 μM) was used in a single experiment in triplicate at 37 °C. Rates are expressed as mean percentage activity ± SEM as a function of percentage activity minus inhibitor. (E) Effect of different Brij-35 surfactant concentrations on the cleavage of the current substrate by TS5-5. Rate data are expressed in nM s^–1^ nM^–1^ of proteinase as the mean ± SD for 3–7 experiments performed in triplicate or quadruplicate. Statistical significance was determined between 0.005% (v/v) Brij-35 and all other surfactant concentrations using a parametric two-tailed unpaired *t* test with Welch’s correction; *p < 0.05, **p < 0.01, ***p < 0.001, ****p < 0.0001. (F) Selectivity of the current substrate for ADAMTS-5 compared to different metalloproteinases. Rate data are expressed as the percentage mean ± SEM of TS5-5 cleavage of the current substrate from 2 to 8 independent experiments in triplicate. Only a single triplicate experiment was performed for MMP-1 as no cleavage was observed up to 72 h at 92 nM. ΔC = catalytic domain, MMP-1 and MMP-3 = full length. For (E) and (F), the substrate concentration and conditions were the same as those for (D).

Förster resonance energy transfer (FRET) substrates are a useful tool for measuring proteinase activity longitudinally due to their capacity to amplify signal intensity through continuous substrate cleavage and potential for high target selectivity.^18^ Composed of a peptide sequence with a blue fluorophore (BlueF) and quencher (Q) on either side of the scissile bond,^19^ the FRET probe does not fluoresce prior to cleavage as the excitation of the BlueF (the donor) is tunneled to the quencher (the acceptor) over distances between 0 and 70 Å (Figure 1A). Upon substrate cleavage, an increase in fluorescence is observed due to an increase in separation between donor and acceptor (>70 Å), resulting in disruption of the FRET process.^19^ Selective *in vivo* FRET substrates have been developed for MMP-13 to monitor disease progression and response to therapy in rodent models of OA.^20−22^ However, these FRET substrates were limited by only being able to distinguish between osteoarthritic joints and healthy joints once damage was already clearly visible by histology. Furthermore, current FRET substrates for ADAMTSs are limited by low rates of cleavage and/or poor target selectivity.^23−26^ Developing a selective ADAMTS-5 FRET substrate would enable earlier diagnostic imaging of osteoarthritic processes and provide more effective tracking of disease progression.

Herein, we use computational docking and combinatorial chemistry to develop two novel FRET peptide substrates with enhanced selectivity and sensitivity for ADAMTS-5 which can be used in *in vitro* systems for monitoring ADAMTS-5 activity. We discuss their potential application for *in vivo* imaging in osteoarthritis and more generally in diseases characterized by elevated expression of ADAMTS-5.

## Results

### Purification and Characterization of ADAMTS-5

The combined catalytic and disintegrin domain of ADAMTS-5 (TS5-5) was selected as the target proteinase because it possesses a molecular weight compatible with combinatorial library beads. These beads will be used to identify hit substrates by screening the combinatorial library against ADAMTS-5 on-resin. The compatibility refers to the molecular weight limit of the proteinase that can diffuse into the poly(ethylene glycol)-polydimethyl acrylamide (PEGA_1900_) resin bead to cleave the substrate on the solid support, and has been reported as 60 kDa.^27^ In addition, purification of TS5-5 delivers the highest yield among all domain deletion mutants.^28^ TS5-5 (Figure 1B) was expressed in HEK293 cells and purified by anti-FLAG affinity chromatography based on a previously published protocol.^29^Figure 1C shows a representative Western blot and Coomassie stain of purified TS5-5, which was obtained in high yield compared to a previous report (15 mg/L vs 0.25 mg/L), at a high level of purity, and at the expected molecular weight (∼37 kDa/∼41 kDa).^28^ The second band at ∼41 kDa is an N-glycosylated form.^28^ To determine the concentration of active TS5-5, active-site titration with the N-terminal domain of the endogenous inhibitor tissue inhibitor of metalloproteinases-3 (N-TIMP-3) was performed (Figure 1D).^30^ Extrapolation of the linear portion of the curve to the *x*-axis gave the concentration of active proteinase (27 nM).

At this point, it was essential to optimize FRET substrate cleavage by TS5-5, as this would maximize signal intensity from hit beads following proteolytic cleavage during combinatorial library screening. Six concentrations of Brij-35 surfactant ranging from 0% (v/v) to 5% (v/v) were tested. Initially, calibration curves for the Abz fluorophore were generated at each Brij-35 concentration to correct for the effect of surfactant concentration on the fluorescence of the released fluorophore. The rates of cleavage of the current substrate *ortho*-aminobenzoyl(Abz)-TESE↓SRGAIY-*N*-3-[2,4-dinitrophenyl]-l-2,3-diaminopropionyl(Dpa)-KK-NH_2_ by TS5-5 were then determined (Figure 1E). These were expressed as nanomolar substrate cleaved per second per nanomolar proteinase, as a variety of different proteinase concentrations were used. A concentration of 0.005% (v/v) Brij-35 gave the highest rate of substrate cleavage (0.11 nM s^–1^ nM^–1^), which was significantly higher than all other surfactant concentrations. Consequently, 0.005% (v/v) Brij-35 was used for all further assays in this study.

Next, the level of selectivity of the current ADAMS-5 FRET substrate was assessed against a panel of MMPs (MMP-1, MMP-2, MMP-3, MMP-9, and MMP-13) and a closely related ADAMTS family member (ADAMTS-4) (Figure 1F). This panel was chosen to measure selectivity across different metalloproteinase families and subfamilies. Rates were expressed as a fraction (percentage) of TS5-5 cleavage of the current substrate, which showed poor overall selectivity for TS5-5, exhibiting >10% cleavage by TS4-4 (22%) and >100% cleavage by the catalytic domain of MMP-2, *i*.*e*., MMP-2ΔC (138%). The activity of each proteinase was confirmed through FRET substrate cleavage (Figure S1), and the active concentration determined *via* titration with either N-TIMP-3 or the small molecule hydroxamate inhibitor CT-1746.^31^ The lack of selectivity of the current ADAMTS-5 substrate Abz-TESE↓SRGAIY-Dpa-KK-NH_2_ further validated our initial strategy to develop a selective FRET peptide substrate for monitoring the activity of this proteinase *in vitro*.

### The Design of Selective ADAMTS-5 FRET Peptide Substrates through Computational Modeling

**Figure 2:**
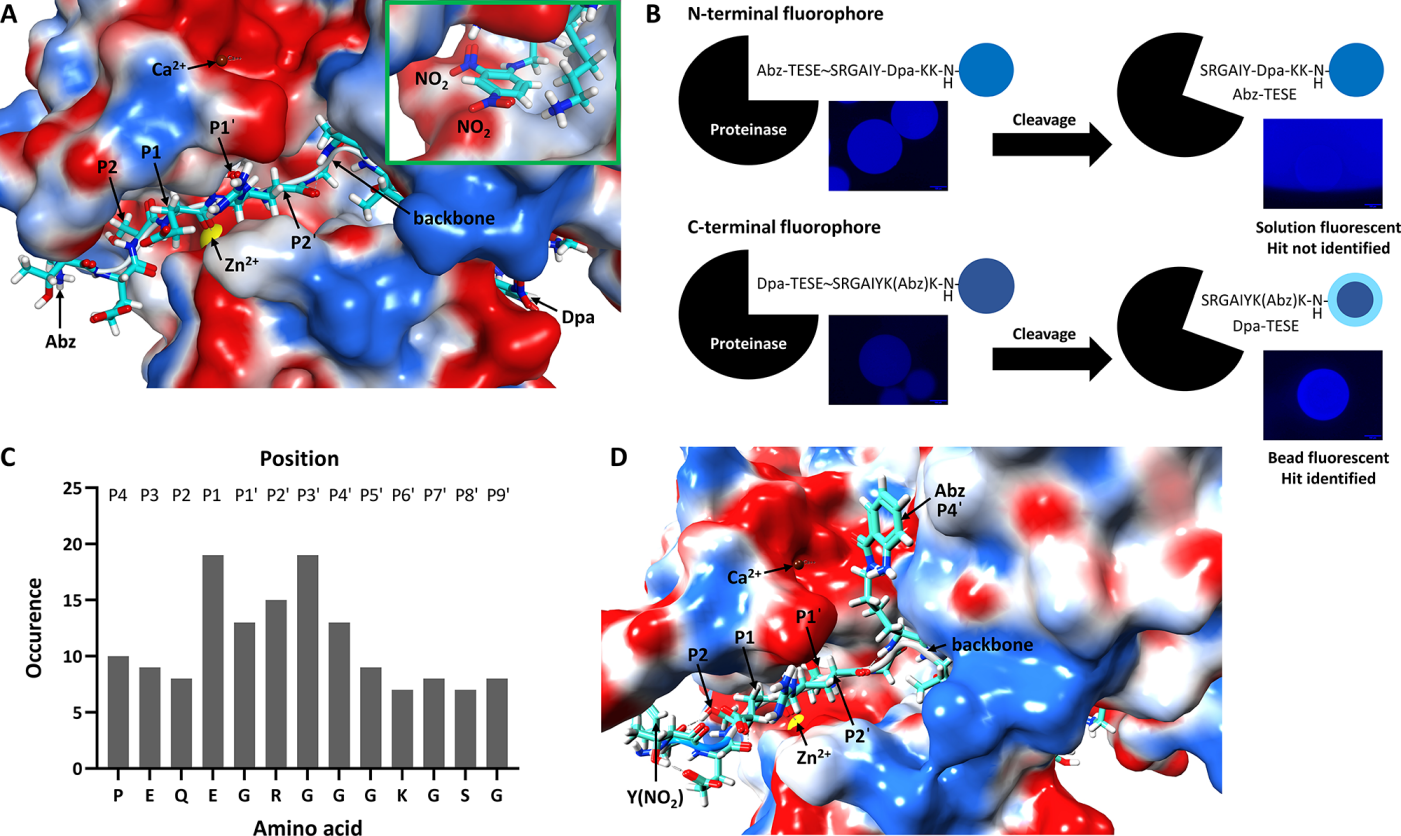
Design of selective ADAMTS-5 FRET peptide substrates through computational docking. (A, D) Docking of Abz-TESE↓SRGAIY-Dpa-KK-NH_2_ (A) and Y(NO_2_)TESESRGK(Abz)IYYKKG (D) into the active site of the crystal structure of TS5-5 (2RJQ, Protein Data Bank) utilizing Molecular Operating Environment software. The green inset in (A) shows magnification of the Dpa quencher and its environment. The negatively and positively charged electrostatic energy surfaces are colored red and blue, respectively. The zinc (Zn^2+^) and calcium (Ca^2+^) ions are represented by yellow and dark red balls, respectively. The backbone of the peptide is depicted as white tubing, with noncovalent interactions denoted by dotted lines. Carbon, hydrogen, oxygen and nitrogen atoms are colored cyan, white, red and blue, respectively. (B) Schematic comparison of N-terminal (*e*.*g*., Abz-TESE↓SRGAIY-Dpa-KK-NH_2_, top) and C-terminal (*e*.*g*., Dpa-TESE↓SRGAIYK(Abz)K-NH_2_, bottom) fluorophore designs for FRET peptide substrates screened against ADAMTS-5 on-resin. The images shown are real examples of beads with these designs but which have different sequences (scale bar = 100 μm). (C) Most commonly occurring residues in peptide positions P4–P9′ of 57 known ADAMTS-5 substrates compiled from CutDB, BRENDA, and MEROPS databases.

Initially, the existing substrate Abz-TESE↓SRGAIY-Dpa-KK-NH_2_ was docked into the active site of TS5-5 (Figure 2A). This model showed an unfavorable location for the Dpa quencher, as there is electrostatic repulsion between the two aromatic nitro groups and the negatively charged electrostatic energy surface (green inset). Moreover, the position of the Abz fluorophore relative to the cleavage site was found to be incompatible with substrate screening against ADAMTS-5 on-resin (Figure 2B). This is because cleavage of test substrates with an N-terminal fluorophore resulted in diffusion of the fluorophore fragment into the surrounding solution. This in turn resulted in increased solution fluorescence and an inability to identify hit beads (Figure 2B, top). Therefore, to guarantee identification of hits during on-resin screening against ADAMTS-5, a C-terminal Abz fluorophore design was chosen for all modeled substrates (Figure 2B, bottom).

Potential novel substrates were modeled *via* optimization of amino acids in each peptide position (P) through sequence alignment of ADAMTS-5 substrates obtained from CutDB, BRENDA and MEROPS databases (Table S1). This led to the identification of the most frequently occurring residues at several P sites (*e*.*g*., E in P1, G in P1′, R in P2′, and G in P3′) (Figure 2C) and thus a potential preference for particular side-chains in the different subsites of ADAMTS-5. To design selective substrates, single, conservative amino acid modifications were made to reduce disruption to ADAMTS-5 cleavage by several nonconservative changes. Docking was carried out with solvation of the substrate-ADAMTS-5 complex in water followed by the introduction of amino acids based on (i) the amino acid alignments in Table S1; (ii) the preference for amino acids in specific positions in Figure 2C; (iii) the presence of favorable or unfavorable electrostatic interactions with the active site; and (iv) steric hindrance between amino acid side chains and residues within the active site. In addition, un-natural amino acids were introduced to further explore binding pockets in the active site. To generate the optimal substrate pose, energy minimization and molecular dynamics simulations were performed after introduction of an amino acid. A modification was retained only if the pose of the resultant substrate was considered favorable based on criteria (iii) and (iv).

To generate the first series of substrates, the Abz fluorophore was moved onto the side chain of lysine (P4′) as there are favorable electrostatic interactions between the aromatic amine and the negatively charged pocket of the proteinase (Figure 2D). In addition, the Dpa quencher was exchanged for 3-nitro-l-tyrosine, Y(NO_2_), as this is a smaller amino acid that is less likely to disrupt binding to the active site. A C-terminal Gly was introduced to facilitate synthesis and fill out the limited space at the C-terminus, while Tyr was added to P7′ as this closely resembles the Dpa quencher in terms of structure and reduces electrostatic repulsion at that position (*cf*. green inset, Figure 2A). The first member of this novel series, namely Y(NO_2_)TESESRGK(Abz)IYYKKG, is shown in Figure 2D and Table S2 (**1**). Further derivatives were designed based on this compound (Table S2) but maintaining a Ser residue in P1′ as this was abundant at this position (11%, Table S1), and can be found in ADAMTS cleavage sites in endogenous substrates such as brevican^32,33^ and versican.^34^ As a P2 Gly is present in the main aggrecan cleavage site, it replaced Ser to give **2**.^35^ A Lys residue was introduced at the N-terminus to increase the solubility of the overall substrate (**3**). The un-natural amino acid β-cyclopropyl-alanine (J) was inserted instead of Ile at P5′ to improve selectivity at that position while maintaining a conservative change (**4**). As multiple substrates in Figure 2C contained a proline residue at P4 (18%), it and 4-hydroxyproline (X) were added to this position (**5** and **6**, respectively). The parent compound **1** was also extended with Thr and Gly residues (**7**) to determine whether longer peptides were necessary for successful cleavage, given the size of many natural substrates of ADAMTS-5 (*e*.*g*., aggrecan).

To optimize placement of the Abz fluorophore, two further series of substrates were designed (Tables S3 and S4). In Table S1, three substrates contain a quencher at the P7′ position, including the current ADAMTS-5 substrate Abz-TESE↓SRGAIY-Dpa-KK-NH_2_. In addition, one of the substrates incorporates a 2,4-dinitrophenyl quencher into the side chain of Lys.^36,37^ This showed that the lysine side chain was open to modification, and thus the K(Abz) fluorophore was moved to P7′. An alanine was used to replace it at P4′ so the initial substrate within the P7′ series, Y(NO_2_)TESESRGAIYK(Abz)KKG (Table S3, **8**), was consistent with the current ADAMTS-5 substrate. Replacement of Lys at P8′ with the shorter 2,4-diaminobutyric acid (B) to give **9** was carried out because interaction of the longer lysine side chain with the “roof” of the binding pocket was considered sterically unfavorable during modeling. A 4-thiazolyl-alanine (Z) was inserted instead of Ala at P4′, so the sulfur atom of the thiazole would have a favorable noncovalent interaction with the positively charged molecular energy surface on the right-hand side (**10**). The insertion of a Pro at P4 (**11**) and a Gly at P2 (**12**) were performed for the same reasons as mentioned in the P4′ fluorophore series.

For the last series of substrates (P9′, Table S4), a shift of K(Abz) to the P9′ position with the concomitant introduction of Ala at P4′ gave the initial compound Y(NO_2_)TESESRGAIYYKK(Abz)G (**13**). Ala was introduced at P4′ to closely resemble the current ADAMTS-5 substrate and the K(Abz) was moved to P9′ as Lys residues were already present and sufficient space was available to accommodate this fluorophore. Further modifications to **13** followed a similar pattern to the P4′ and P7′ series; a Pro at P4 (**14**) and a Gly at P2 (**17**). Leu was introduced as a conservative change at P7′ (**15**), and J was also used at that position (**16**).

A summary of the modeled P4′, P7′ and P9′ FRET substrate series is shown in Table 1, with a snapshot of each amino acid modification in Tables S2–S4. All modeled substrates were synthesized by Fmoc-based solid-phase peptide synthesis (Fmoc-SPPS), and the bulk of the material was left on resin for screening. A small sample of each peptide was cleaved from the resin and characterized by HRMS to confirm successful synthesis of the desired peptide substrates (Table S5).

**Table 1:**
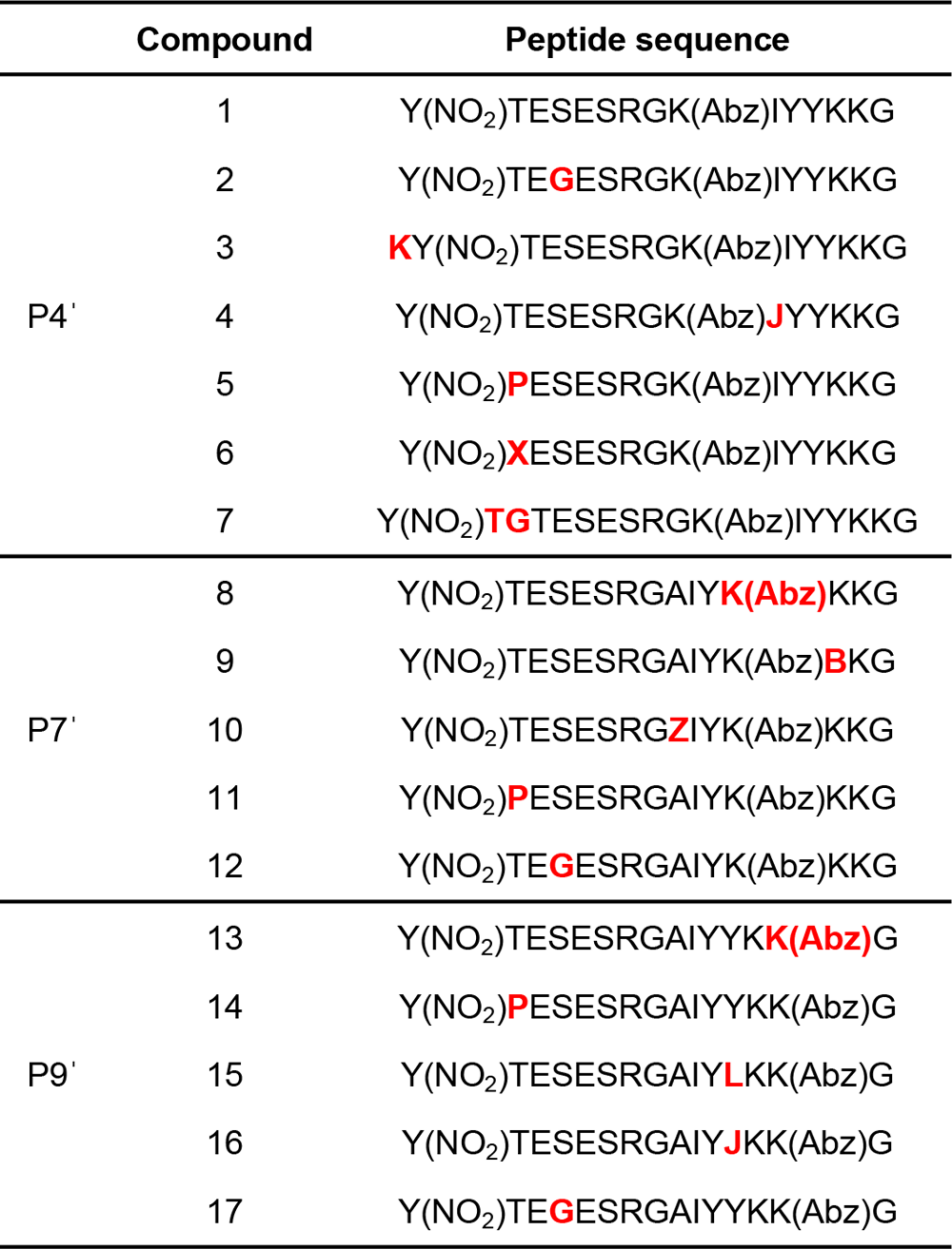
Summary of Modeled ADAMTS-5 FRET Peptide Substrates Synthesized by Manual Fmoc-SPPS^a^

a Key: J = β-cyclopropyl-alanine; X = 4-hydroxyproline; B = 2,4-diaminobutyric acid; Z = 4-thiazolyl-alanine. Residues altered within each fluorophore series are colored in red.

### Screening of Modeled Substrates to Identify a Foundation for an ADAMTS-5 Combinatorial Library

**Figure 3:**
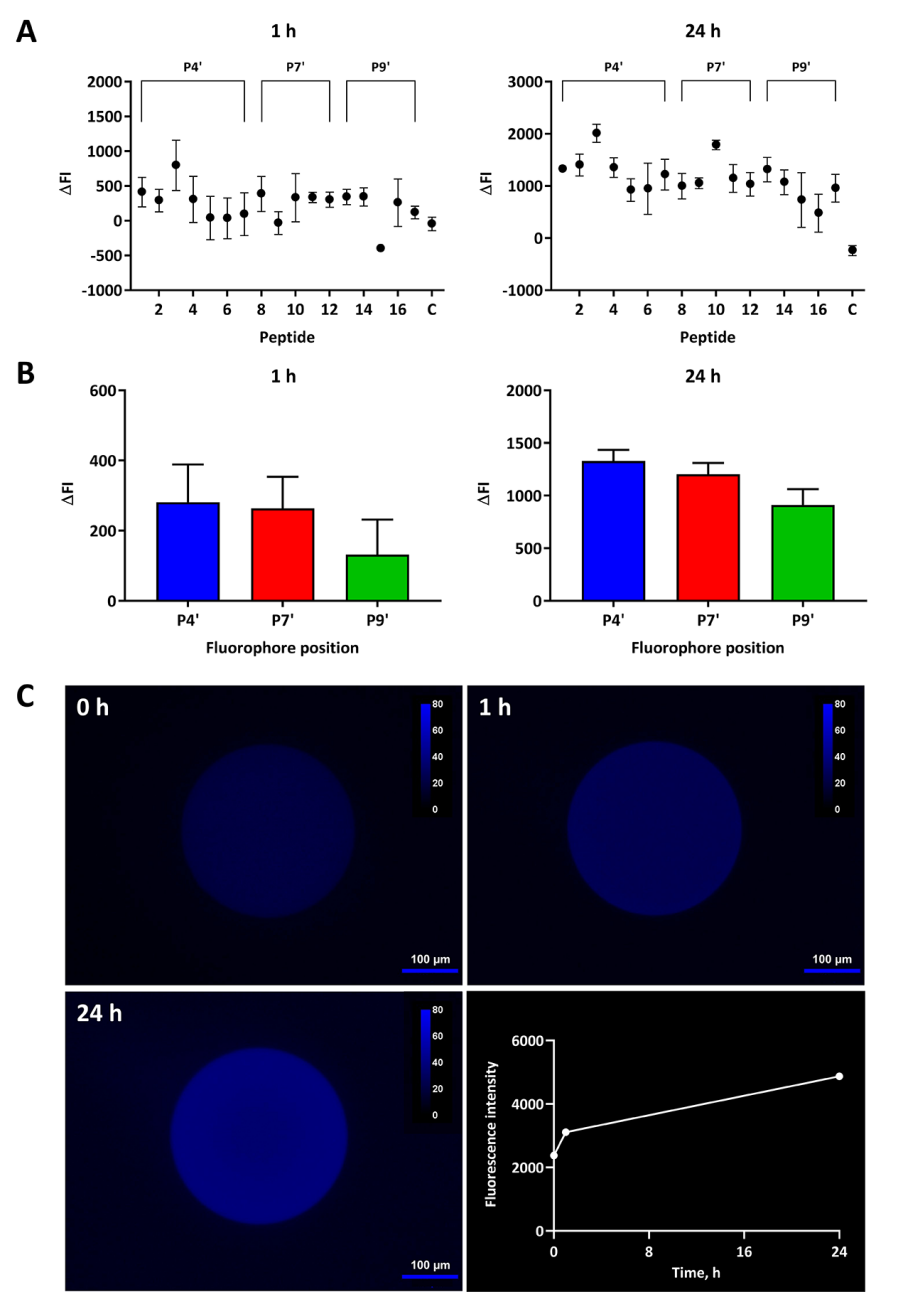
Screening modeled FRET peptide substrates on-resin to discover starting points for an ADAMTS-5 combinatorial library. (A, B) Cleavage of on-resin FRET peptide substrates by TS5-5. (A) Variation in mean fluorescence intensity (ΔFI) ± SEM of substrate beads incubated with TS5-5 (ΔFI_TS5-5_) subtracted from the fluorescence intensity of beads incubated without TS5-5 (ΔFI_control_) after 1 h and 24 h for three independent experiments performed in single replicate. For each measurement, ΔFI = background-subtracted FI at time t–background-subtracted FI at time 0. (B) ΔFI ± SEM summed by fluorophore position (P4′, P7′, and P9′) after 1 h and 24 h for data in (A). For all substrates, cleavage was measured in TNC buffer with 0.005% (v/v) Brij-35 under a fluorescence microscope after incubation at 37 °C (λ_ex_ = 350 nm, λ_em_ = 438 nm). C = control, bead without substrate. (C) Fluorescent images of a bead containing KY(NO_2_)TESESRGK(Abz)IYYKKG (**3**) and quantitative assessment of the change in total fluorescence intensity of the imaged bead after 0 h, 1 h and 24 h incubation with TS5-5 (0.5 μM) in TNC buffer with 0.005% (v/v) Brij-35. The bead was imaged under a fluorescent microscope (λ_ex_ = 350 nm, λ_em_ = 438 nm) and images processed and false colored with Fiji software. The change in total fluorescence intensity over time was measured using a three-point circle (*r* = 50 μm) in cellSens Dimension software and corrected through subtraction of background fluorescence.

Modeled substrates bound to PEGA_1900_ resin were incubated with soluble TS5-5 to determine whether any cleavage could be observed (Figure 3A). After 1 h, no substantial increase in mean fluorescence intensity (ΔFI) from cleavage by TS5-5 was observed for individual substrates (*i*.*e*. ΔFI < 1000), except for peptide **3** (796). The decrease in fluorescence intensity for peptide **15** (−402) resulted from an unusually high measurement for the control bead, which can occur due to natural variations in FI between individual substrate beads. Summing ΔFI by fluorophore position revealed a higher fluorescence increase for P4′ and P7′ compared to P9′ substrates (Figure 3B, *cf*. 281 and 264 to 132). However, this was not significant, thereby revealing no clear preference for fluorophore position 1 h after incubation with TS5-5.

After 24 h, a higher increase in ΔFI from cleavage by TS5-5 (*i*.*e*., >1000) was found for most peptide substrates. The slight decrease in ΔFI for control beads (−240) was due to differences in FI between individual beads during measurement. Looking at the position of the fluorophore, a stepwise decrease in mean ΔFI was observed when it was moved from P4′ (1329) to P7′ (1202) to P9′ (911). As the P4′ series had the highest ΔFI after 24 h (1329), the P4′ fluorophore position was found to be optimal for substrate cleavage. Within this series, peptide **3** had the highest ΔFI after 24 h (2010) and was therefore selected as the lead candidate for the development of a combinatorial library. Images of the change in fluorescence intensity of a peptide **3** bead following incubation with TS5-5 clearly show the expanding “ring” of fluorescence as the substrate is cleaved gradually over 24 h (Figure 3C, numbered panels). Quantitation of fluorescence intensity during this time indicated a plateau prior to the 24 h time point (Figure 3C, bottom-right panel).

### The Design, Synthesis, and Screening of an ADAMTS-5 Combinatorial Library

**Figure 4:**
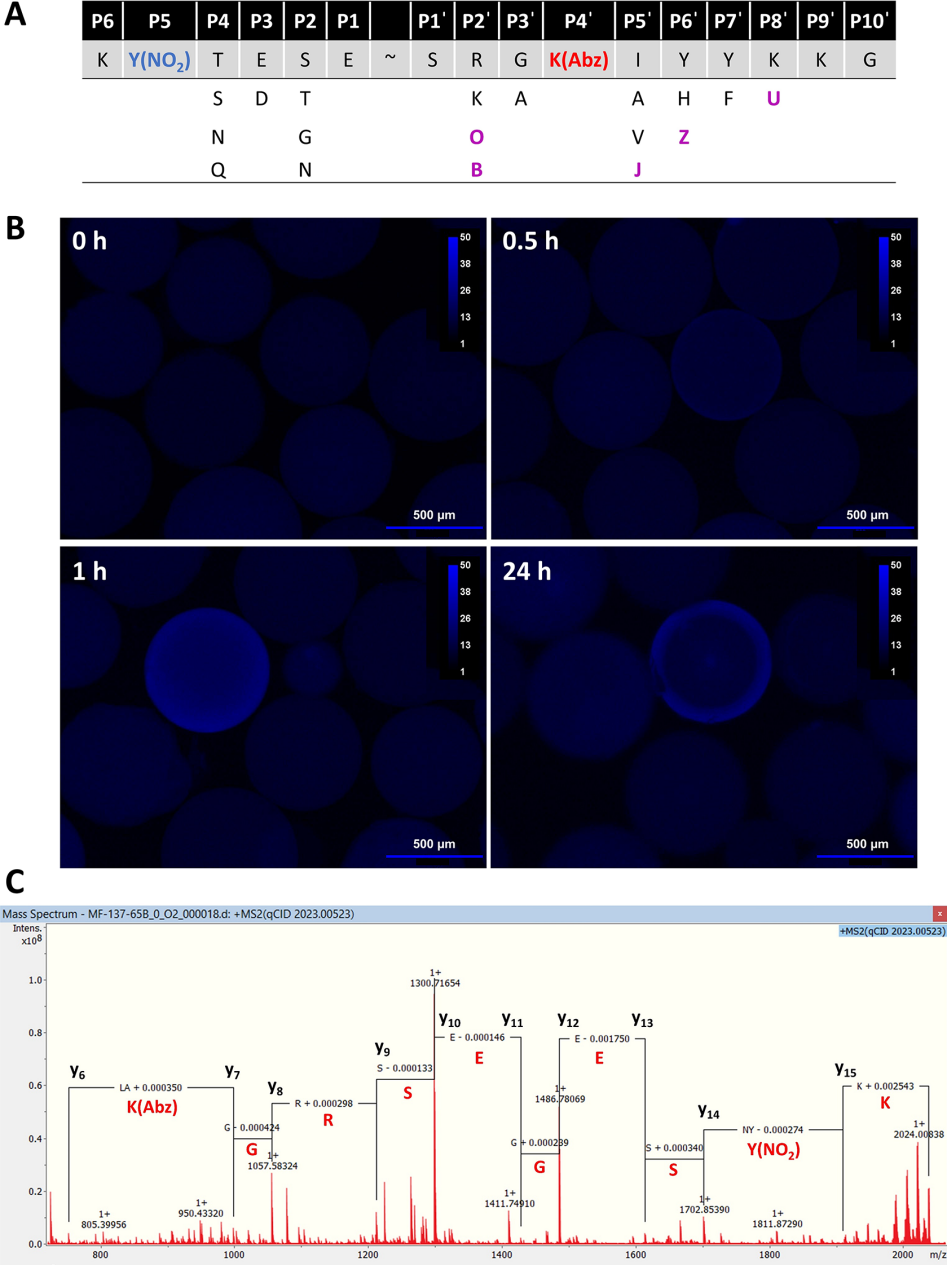
Design and screening of an ADAMTS-5 combinatorial substrate library and identification of peptide hits. (A) Design of an ADAMTS-5 combinatorial library. The FRET peptide substrate KY(NO_2_)TESESRGK(Abz)IYYKKG (**3**) was used as the basis for the library, with fluorophore and quencher positions denoted in red and blue, respectively. Amino acid changes at specific positions are shown by their single letter code, except unnatural amino acids (shaded in purple) which are abbreviated as follows: O = ornithine, B = 2,4-diaminobutyric acid, J = β-cyclopropyl-alanine, Z = 4-thiazolyl-alanine, U = 2,3-diaminopropionic acid. (B) Fluorescent images of library beads prior to screening with TS5-5 (0 h), after screening with TS5-5 (0.5 μM, 30 min, 1 h) and after deactivation with TFA (24 h) in TNC buffer with 0.005% (v/v) Brij-35. Beads were imaged under a fluorescent microscope (λ_ex_ = 350 nm and λ_em_ = 438 nm) and images processed and false colored using Fiji software. (C) Example mass spectrum of a peptide hit identified from ADAMTS-5 combinatorial library screening. Tandem mass spectrum following fragmentation of [M – O + H]^+^ (2023.00523 u), where M = mass of the quasi-molecular ion. For clarity, only selected residue losses are shown and only fragment ion peaks that could be identified as b ions or y ions have been labeled. The spectrum was analyzed and processed using Bruker Compass DataAnalysis software.

The structure of peptide **3** formed the foundation of the combinatorial library, which consisted of both fixed and unfixed residue positions (Figure 4A). Fixed positions were those considered optimized through docking and amino acid alignments. Specifically, K in P6 and P9′ were retained to improve solubility, E in P1 and S in P1′ occur frequently in known ADAMTS-5 substrates (Figure 2C and Table S1) and G in P10′ facilitated syntheses and reduced steric hindrance at the C-terminus. Unfixed positions were modified conservatively to improve substrate cleavage by ADAMTS-5 without complete abrogation of cleavage. For example, A in P3′ was introduced alongside G, as it is not functionally different. Unnatural amino acids were added to improve selectivity at specific positions. For example, shorter side chain derivatives of K were incorporated *via* O (ornithine) and B (2,4-diaminobutyric acid) at P2′.

A combinatorial library of FRET peptide substrates was synthesized using standard Fmoc-SPPS combined with the “split-mix” method^38^ to give a theoretical number of 12288 unique peptide sequences represented on ∼100 000 beads. The total number of unique sequences was intentionally kept below 50 000 to facilitate hit identification by MALDI and tandem mass spectrometry. Library screening was then carried out by incubating beads with TS5-5 (0.5 μM) for 1 h with monitoring of fluorescence every 30 min. Representative images of library beads can be seen in Figure 4B. At 0 h, the fluorescence of all beads was quenched due to the presence of Y(NO)_2_, indicating successful synthesis of peptides. After 30 min, a faint “ring” of fluorescence could be seen on the edges of some beads. After 1 h, substrate cleavage in beads was clearly observed. The TS5-5 proteinase was then deactivated with 2% (v/v) TFA to ensure successful identification of hit beads using mass spectrometry (by retaining a sufficient number of intact sequences) and to easily isolate these hits from the rest of the library by reducing cleavage of other substrates. After several washing steps the library was stored at 4 °C overnight following which hit beads were manually removed under a fluorescent microscope. A total of 20 beads were isolated. FRET peptide substrates were cleaved from beads using an aqueous solution of triethylamine prior to identification *via* MALDI and tandem mass spectrometry.

### Identification of ADAMTS-5 Peptide Hits through MALDI and Tandem Mass Spectrometry

An initial MALDI was carried out to determine the mass of the quasi-molecular ion, which was then subjected to collision-induced dissociation to generate fragment ions. The fragmentation pattern was used to elucidate the final sequence of each hit by *de novo* peptide sequencing using commercially available Bruker software. An example spectrum obtained for the peptide hit KY(NO_2_)SEGESRGK(Abz)JYFKKG is shown in Figure 4C and reveals how the peptide sequence was determined from the y-ion series. The in-house software LibMSCalc^39^ was used to score how well the experimental fragmentation pattern of each hit sequence matched the predicted fragmentation pattern. The higher the score, the better the match and the greater the probability that the initially determined sequence was the actual sequence. Nine hits from the initial 20 were conclusively identified using this approach. Peptide hits were synthesized commercially by Fmoc-SPPS, cleaved from resin, and purified by HPLC (≥95% purity). Table 2 shows a summary of the hit peptide substrates, their characterization by HRMS and their LibMSCalc scores. The tandem mass spectra used to identify the sequence of each peptide hit are shown in Figures S2–S10 with the HPLC UV and mass traces of the pure peptides thereafter.

**Table 2:**
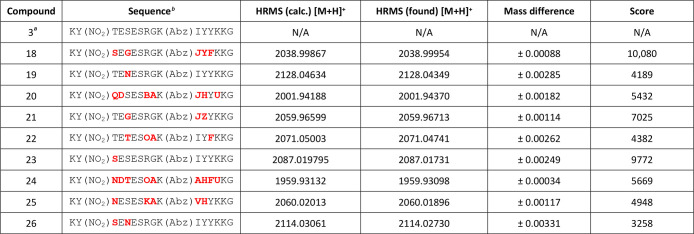
Summary of Hit ADAMTS-5 FRET Peptide Substrates Identified from Library Screening and Characterized by Mass Spectrometry

a Parent compound for combinatorial library synthesis added for sequence comparison purposes.

b Residues that differ from the parent compound in that position are bolded in red. Key: O = ornithine, B = 2,4-diaminobutyric acid, J = β-cyclopropyl-alanine, Z = 4-thiazolyl-alanine, U = 2,3-diaminopropionic acid.

### Determination of Cleavage Kinetics, Selectivity, and Sensitivity of ADAMTS-5 Hit Peptide Substrates

**Figure 5:**
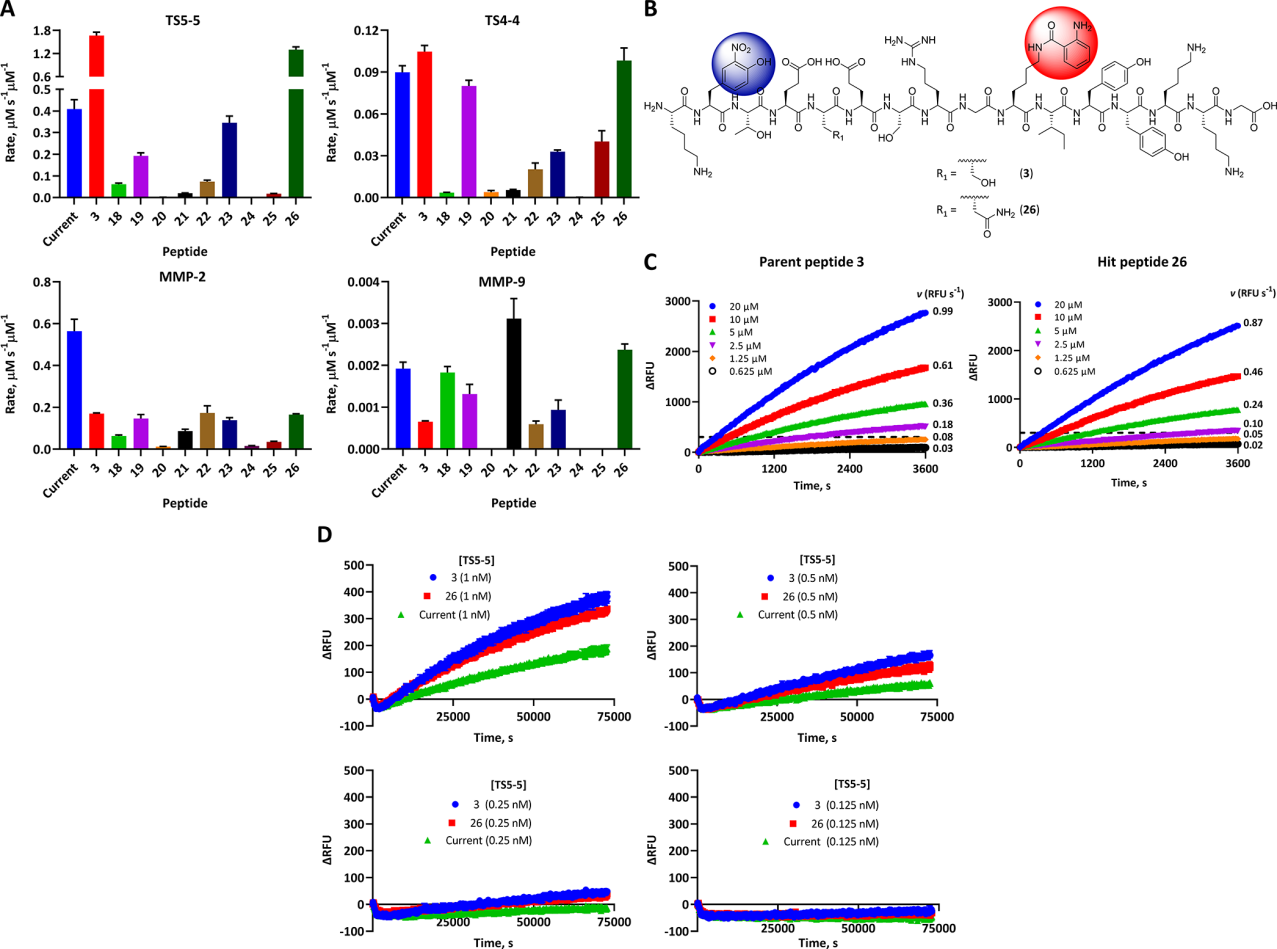
The novel FRET peptide substrates are selective and sensitive for detecting ADAMTS-5 activity *in vitro*. (A) Selectivity of novel FRET peptide substrates against ADAMTS and MMP metalloproteinases. Rate data for cleavage of the current substrate, library parent peptide **3** and hit peptides **18**–**26** (all 20 μM) by TS5-5, TS4-4, MMP-2ΔC, and MMP-9ΔC. The rates for each metalloproteinase are expressed in μM s^–1^ μM^–1^ of proteinase as the mean ± SEM from 3–4 independent experiments performed in triplicate at 37 °C. ΔC = catalytic domain. (B) Structure of the most rapidly cleaved and selective peptide substrates **3** and **26**, with fluorophore and quencher shown in red and blue, respectively. (C) Comparison between the rates of cleavage of **3** and **26** at varying substrate concentrations (20–0.63 μM) but at a fixed concentration of TS5-5 (57 nM). The rate (*v*) is expressed in relative fluorescence units (RFU) s^–1^ from fitting only the linear portion of the curve to a linear regression, with each time point expressed as ΔRFU (RFU at time *t* – RFU at time 0) ± SEM from a single experiment conducted in triplicate at 37 °C. The dotted line denotes ΔRFU = 300. (D) Comparison between the current substrate, parent compound **3** and hit peptide **26** in terms of the lowest substrate concentration that can detect ADAMTS-5 activity. Rates were measured at a fixed concentration of substrate (5 μM) with 1 nM, 0.5 nM, 0.25 nM and 0.125 nM of TS5-5 over 20 h. Each time point is expressed as ΔRFU ± SEM from a single experiment conducted in triplicate at 37 °C.

To confirm cleavage by ADAMTS-5, compound **3** and hit peptides **18**–**26** were incubated with TS5-5 in solution and the rates expressed as micromolar substrate cleaved per second per micromolar proteinase, as several different proteinase concentrations were used (Figure 5A, top left).

Compared to the current substrate Abz-TESE↓SRGAIY-Dpa-KK-NH_2_, compound **3** showed an ∼4-fold improvement in cleavage by TS5-5, implying accurate and successful docking of **3** into the active site. For hit peptides, compound **26** showed an ∼3-fold improvement in cleavage by TS5-5 compared to the current substrate. Peptide hits that differed by 4–5 residues from parent compound **3** had considerably lower rates of cleavage by TS5-5: 7.38 × 10^–2^ μM s^–1^ μM^–1^ for **22** and 1.78 × 10^–2^ μM s^–1^ μM^–1^ for **25**, for example. In cases where peptides possessed more than one unnatural amino acid, *e*.*g*., **24** (O and U) and **20** (B, J and U), the lowest rates of cleavage rates were recorded: 1.41 × 10^–4^ and 1.22 × 10^–3^ μM s^–1^ μM^–1^, respectively. These results show that the introduction of unnatural amino acids into peptide substrates was unfavorable and led to lower rates of cleavage by ADAMTS-5.

To investigate this further, catalytic rate constants (*k*_cat_ values) and Michaelis constants (*K*_m_ values) were determined for the best cleaved substrates **19**, **23**, and **26** as well as parent compound **3** and the current substrate. These data were corrected for the inner filter effect^40^ and are shown in Table 3. This effect is most pronounced at substrate concentrations >20 μM.^41^ At these concentrations, upon cleavage of the FRET substrate and separation of the fluorophore and quencher, the fragment containing the “free” fluorophore is substantially quenched by uncleaved intact peptides or cleaved quencher fragments, reducing the amount of detected fluorescence. This decrease in detected fluorescence affects the cleavage rate, which in turn results in less accurate kinetic parameters after fitting to the Michaelis–Menten equation.^40,42^ Correction factors and both uncorrected and corrected curves for the current substrate, **3**, **19**, **23**, and **26** can be found in Tables S6–S10 and Figures S11–S15, respectively. The higher values obtained for both *k*_cat_ and *K*_m_ for all substrates highlight the importance of inner-filter correction for determining accurate kinetic parameters for proteinase substrates.

**Table 3:** Kinetic Parameters for Cleavage of the Current ADAMTS-5 Substrate, Library Parent Peptide, and Selected Hit Peptides^a^

peptide	sequence	*k*_cat_, **s**^-1^	*K*_m_, μM	*k*_cat_**/***K*_m_, M^-1^ s^-1^
current	Abz-TESE↓SRGAIY-Dpa-KK-NH_2_	3.61 ± 0.31	125.2 ± 5.0	2.91 × 10^4^ ± 3.09 × 10^3^
**3**	KY(NO_2_)TESESRGK(Abz)IYYKKG	1.87 ± 0.15	29.5 ± 1.7	6.32 × 10^4^ ± 1.84 × 10^3^
**19**	KY(NO_2_)TENESRGK(Abz)IYYKKG	0.53 ± 0.014	30.5 ± 2.0	1.75 × 10^4^ ± 1.08 × 10^3^
**23**	KY(NO_2_)SESESRGK(Abz)IYYKKG	1.14 ± 0.11	64.6 ± 4.6	1.82 × 10^4^ ± 2.94 × 10^3^
**26**	KY(NO_2_)SENESRGK(Abz)IYYKKG	3.32 ± 0.31	76.9 ± 6.4	4.44 × 10^4^ ± 6.69 × 10^3^

a N.B. All values represent mean ± SEM from 3–4 independent experiments performed in triplicate.

Compared to the current substrate, parent compound **3** and hit peptides **19** and **23** had ∼2-fold, ∼7-fold, and ∼3-fold lower catalytic rate constants, respectively. In contrast, the *k*_cat_ value for **26** (3.32 s^–1^) was similar to that reported for the current substrate.^23^ Nevertheless, none of these peptides had improved *k*_cat_ values relative to the current substrate, indicating that after formation of the ADAMTS-5-substrate complex, conversion of substrate to product was slower. Conversely, the *K*_m_ value was found to decrease for **3** and all hit peptide substrates compared to the current substrate. For **3** and **19**, this value fell by ∼4-fold to 29.5 μM and 30.5 μM, respectively. For **23** and **26**, a smaller ∼2-fold decrease was observed. The lower *K*_m_ values for **3** and **19** indicate stronger substrate binding to ADAMTS-5 and greater stability of the subsequent complex. Finally, the highest catalytic efficiencies (*k*_cat_/*K*_m_ values) were recorded for parent compound **3** (6.32 × 10^4^ M^–1^ s^–1^) and hit peptide **26** (4.44 × 10^4^ M^–1^ s^–1^) (Figure 5B). Both showed more effective cleavage by TS5-5 than the current substrate (*k*_cat_/*K*_m_ = 2.91 × 10^4^ M^–1^ s^–1^). Overall, these kinetic values are in a similar range to those determined for other fluorogenic ADAMTS substrates, such as 5-carboxyfluorescein (5-FAM)-AE↓LQGRPISIAK-*N*,*N*,*N′*,*N′*-tetramethyl-6-carboxyrhodamine (TAMRA) cleavage by ADAMTS-4.^24^ In addition, *n-*octanol/water partition coefficients (cLog*P* values) revealed these substrates to have high aqueous solubility (Table S11), and solubility issues were not encountered during the determination of kinetic parameters.

We next assessed the selectivity of parent compound **3** and all hit peptides for ADAMTS-5 by comparing their rate of cleavage by TS5-5 to a panel of metalloproteinases. This panel comprised TS4-4 and MMP-2ΔC (as both cleaved the current substrate most rapidly) and MMP-9ΔC as it belongs to the gelatinase subfamily with MMP-2ΔC (Figure 5). In the case of TS4-4 (Figure 5A, top right), **3** and hit peptides **19** and **26** showed similar rates of cleavage to the current substrate (*cf*. 0.08–0.10 to 0.090 μM s^–1^ μM^–1^) revealing no change in selectivity for TS4-4. All other peptides showed reduced rates of cleavage by TS4-4 compared to the current substrate. For MMP-2ΔC (Figure 5A, bottom left), the rate of substrate cleavage was not only reduced for parent compound **3**, but also for all hit peptides. More specifically, the rate decreased from 0.56 μM s^–1^ μM^–1^ to between 0.17 and 0.012 μM s^–1^ μM^–1^ for the highest and lowest rates of cleavage, respectively. For MMP-9ΔC (Figure 5A, bottom right), all substrates were cleaved poorly (<4.0 × 10^–3^ μM s^–1^ μM^–1^), with hit peptides **20**, **24**, and **25** showing no signs of cleavage after 48 h at an MMP-9 concentration of 30 nM. Overall, the most selective substrates for ADAMTS-5 were found to be parent compound **3** and hit peptide **26**. The former showed an ∼16-fold preference for TS5-5 over TS4-4, an ∼10-fold preference over MMP-2ΔC and an ∼2561-fold preference over MMP-9ΔC. The latter displayed selectivity preferences of ∼13-fold, ∼8-fold, and ∼548-fold over TS4-4, MMP-2ΔC, and MMP-9ΔC, respectively. In contrast, the current substrate exhibited an ∼5-fold preference for TS5-5 over TS4-4, an ∼0.7-fold preference over MMP-2ΔC, and an ∼213-fold preference over MMP-9ΔC.

The sensitivity of **3** and **26** for detection of ADAMTS-5 activity was then evaluated by determining the lowest concentration of substrate that was detectibly cleaved by TS5-5 above the background change in fluorescence intensity of substrate (*i*.*e*., ΔRFU > 300, Figure 5C). At a TS5-5 concentration of 57 nM, 2.5 μM, and 5 μM concentrations of **3** and **26** could be used to detect cleavage rates with ΔRFU ≥ 300 after 1 h. To investigate substrate sensitivity to detecting TS5-5 activity, a low nanomolar concentration of TS5-5 was combined with a 5 μM concentration of **3** and **26** and compared to the current substrate (Figure 5D). Substrates **3**, **26**, and current were all able to detect TS5-5 activity above background at a 1 nM concentration of TS5-5 after 20 h (ΔRFU > 100, top-left panel). When the concentration was decreased to 0.5 nM TS5-5, only substrates **3** and **26** were able to maintain a ΔRFU > 100 after 20 h, with ΔRFU = 59 for the current substrate (Figure 5D, top-right panel). The poor sensitivity of the current substrate became apparent at 0.25 nM TS5-5, where it was no longer able to detect TS5-5 activity above background fluorescence after 20 h, while **3** and **26** gave ΔRFU > 30 (bottom-left panel). None of the three substrates was able to detect the activity of 0.125 nM TS5-5 above background fluorescence after 20 h (bottom-right panel). The library parent peptide **3** and hit peptide **26** are therefore able to detect ADAMTS-5 activity at concentrations as low as 0.25 nM using 5 μM substrate for experiments performed for ∼20 h. In summary, these substrates display improved detection sensitivity for ADAMTS-5 over the current substrate, in addition to the enhanced cleavage rate and better overall selectivity described earlier.

## Discussion and Conclusions

We have developed two FRET peptide substrates for selectively and sensitively monitoring ADAMTS-5 activity. Substrate **3** was identified through computational docking and amino acid replacements of the current ADAMTS-5 substrate Abz-TESE↓SRGAIY-Dpa-KK-NH_2_. Substrate **26** was discovered through the screening of a combinatorial library designed through amino acid replacements of substrate **3**. Compared to the current ADAMTS-5 substrate, **3** and **26** showed ∼4-fold and ∼3-fold higher overall cleavage rates by ADAMTS-5 and ∼2-fold and ∼1.5-fold higher catalytic efficiencies, respectively. In addition, they exhibited ∼16-fold and ∼13-fold selectivity for ADAMTS-5 over ADAMTS-4 (∼5-fold for the current substrate); ∼10-fold and ∼8-fold selectivity over MMP-2 (∼0.7-fold for the current substrate); and ∼2561-fold and ∼548-fold selectivity over MMP-9, respectively (∼213-fold for the current substrate). Both substrates had improved sensitivity compared to the current substrate and were able to detect very low nanomolar concentrations of ADAMTS-5.

Although hit peptides **3** and **26** showed improvements in overall catalytic efficiencies compared to the best current ADAMTS-5 substrate, these parameters primarily resulted from an improvement in peptide *K*_m_ values (*cf*. 29.5 μM for **3** and 76.9 μM for **26** to 125.2 μM for current). In contrast, the *k*_cat_ values were either similar (**26**, 3.32 s^–1^) or slightly lower (**3**, 1.87 s^–1^) compared to the current substrate (3.61 s^–1^). This indicates that modeling primarily strengthened interactions between the substrate and the active site of ADAMTS-5, reducing *K*_m_ and improving substrate recognition. Improved *k*_cat_ values could be obtained by optimizing interactions between the transition state of the substrate and the enzyme active site based on detailed structural analysis of reaction intermediates. Alternatively, *in silico* modeling of the transition state and analysis of key interactions could optimize both *k*_cat_ and *K*_m_, potentially delivering more rapidly cleaved and selective ADAMTS-5 substrates.

This work has highlighted the need to identify selective ADAMTS-5 substrates due to the poor selectivity and sensitivity of the best currently available ADAMTS-5 FRET probe. The combination of computational docking and combinatorial library screening was effective at identifying more than one selective ADAMTS-5 substrate, despite follow-up library screening not delivering a more rapidly cleaved substrate than parent compound **3**. The identification of multiple selective substrates is especially important for their potential utilization in studies *in vivo*, as poor solubility, renal toxicity, and rapid clearance times typically result in high attrition rates for many imaging probes. A higher success rate for *in vivo* use is thus more likely with more substrates to test and use as a starting point for the development of further derivatives.

To the best of our knowledge, no FRET substrates currently exist for measuring ADAMTS-5 activity *in vivo*. The absence of these substrates is likely due to a combination of factors: the difficulty in sensitively and selectively detecting ADAMTS activity in the joint *in vivo*, rapid clearance from the joint and the high expense associated with introducing *in vivo* fluorophore/quencher pairs into existing *in vitro* FRET substrates.^43^ As a result of these factors, our efforts were initially focused on developing a highly selective and sensitive *in vitro* ADAMTS-5 probe. Nevertheless, there is no reason to believe that **3** and **26** could not be adapted to *in vivo* substrates through the introduction of an *in vivo* compatible fluorophore/quencher pair. This is especially the case given the space available for these larger moieties in the docked substrate model of **3** (Figure 2D) and is something that we are currently pursuing.

The development of the ADAMTS-5 FRET peptide substrates **3** and **26** represents a significant advance in the ability to selectively measure the activity of ADAMTS-5 in the context of osteoarthritis. This is because ADAMTS-5 is the major aggrecanase responsible for degradation of articular cartilage in surgical mouse models of osteoarthritis,^3,4^ and also appears to play a key role in human disease.^5−7^ ADAMTS-5 activity could thus be used as a potential clinical biomarker for human osteoarthritis, with FRET probes used for diagnostic and prognostic purposes, especially since aggrecan degradation occurs early in disease and prior to the degradation of type II collagen.^8−12^ Furthermore, their high level of sensitivity for detecting ADAMTS-5 activity lends their use to effectively measuring responses to therapeutic treatments. Further work will be necessary to develop and optimize these substrates for use in biological studies.

To conclude, both **3** and **26** are currently the most selective ADAMTS-5 FRET peptide substrates described to date and may be used to detect ADAMTS-5 activity *in vitro*. Following adaptation to *in vivo* probes, they could be used to measure ADAMTS-5 activity in osteoarthritis as well as other diseases characterized by elevated expression of ADAMTS-5, such as colorectal cancer.^44^

## Experimental Section

### Expression of Metalloproteinases

The metalloproteinases MMP-1, MMP-3, and MMP-9ΔC were gifts from Professor Gillian Murphy (Cambridge University, UK) and MMP-13Δ*C* was purchased from Enzo Life Sciences (Exeter, UK). The metalloproteinase domain deletion mutants TS4-4, TS5-5, and proMMP-2ΔC were cultured from previously transfected stocks of HEK293 cells.^23,28,45^ Tissue culture of TS4-4 and TS5-5 has been described in a previous publication.^29^ In brief, HEK293 cells were cultured for at least 1 week in Dulbecco’s modified Eagle’s medium (DMEM) containing 10% (v/v) fetal bovine serum (FBS), 1% (v/v) penicillin-streptomycin (PS), and hygromycin B (100 μg/mL for TS4-4 and 800 μg/mL for TS5-5 and proMMP-2ΔC). Wells were seeded for expansion to intermediate (20 mL) and then large (50 mL) tissue culture dishes. Once complete confluence had been achieved in large culture dishes, media were changed to serum-free DMEM containing sterile-filtered 0.2% (w/v) lactalbumin enzymatic hydrolysate in distilled water and 1% (v/v) PS. Media (∼500 mL) were collected every 3–5 days (TS4-4, proMMP-2ΔC) or every 7 days (TS5-5) and centrifuged (1146 *x* g, 10 min, 4 °C). The supernatant was decanted and filtered using a 0.22 μm vacuum filter and the resultant solution purified by affinity chromatography.

### Purification of TS4-4 and TS5-5 by FLAG Affinity Chromatography

This purification has been described previously.^29^ In brief, the following buffers were used: equilibration buffer consisting of 50 mM Tris-HCl, pH 7.5, and 500 mM NaCl; wash buffers of 0.2 M glycine-HCl pH 6.0 and pH 5.0; an elution buffer of 0.2 M glycine-HCl pH 3.0 and a neutralization buffer of 2 M Tris-HCl, pH 7.5. Glycine buffers were filtered through a 0.22 μm vacuum filter and stored at 4 °C. A slurry of anti-FLAG M2 affinity gel (2 column volumes, CVs, where 1 CV = 1.5 mL) was packed into a protein purification column using equilibration buffer (2.5 mL/min) on an ÄKTA prime 2.0 system (Cytiva, Little Chalfont, UK). After washing with equilibration buffer (13 CVs, 2.0 mL/min), collected medium (∼500 mL) was loaded onto the column while on ice (2.0 mL/min). The column was sequentially washed with 0.2 M glycine-HCl pH 6.0 and pH 5.0 (13 CVs each, 2.0 mL/min) and bound proteinase eluted with 0.2 M glycine-HCl pH 3.0. Fractions were collected (1 mL) and immediately neutralized with 2 M Tris-HCl, pH 7.5 (100 μL) before storage at −20 °C prior to analysis by SDS-PAGE and Western blotting. A final column wash was performed with equilibration buffer at the end of the purification (13 CVs, 2.0 mL/min).

### Purification of proMMP-2Δ*C* by Gelatin-Sepharose Affinity Chromatography

Purification was performed in the cold room (4 °C). The following buffers were used: equilibration buffer consisting of 50 mM Tris-HCl, pH 7.5, 150 mM NaCl, 10 mM CaCl_2_ and 0.02% (w/v) NaN_3_ (TNC); dilution buffer comprising 100 mM Tris-HCl, pH 8.0, with 0.02% (w/v) NaN_3_; and 1% (v/v) DMSO and 5% (v/v) DMSO TNC, pH 7.5, wash and elution buffers, respectively. A slurry of gelatin-Sepharose 4B affinity resin (1 CV, where 1 CV = 25 mL) was packed into an XK 26/20 column (Cytiva, Little Chalfont, UK) using deionized water (5 CVs, 7.0 mL/min) on an ÄKTA prime 2.0 system (Cytiva, Little Chalfont, UK). The column was washed with 5% (v/v) DMSO TNC buffer, pH 7.5 (2 CVs, 2.0 mL/min) and equilibrated with TNC buffer (4 CVs, 2.0 mL/min). Medium containing proMMP-2Δ*C* was diluted 1:1 with the dilution buffer and loaded onto the column (2.0 mL/min). The column was washed with 1% (v/v) DMSO TNC (2 CVs, 2.0 mL/min). Bound proMMP-2Δ*C* proteinase was eluted with 5% (v/v) DMSO TNC buffer, pH 7.5 and fractions (5 mL) collected and frozen at −20 °C. A final column wash was performed with equilibration buffer at the end of the purification (2 CVs, 2.0 mL/min).

### Removal of DMSO from proMMP-2Δ*C* Fractions by Native Sephacryl Affinity Chromatography

A slurry of Sephacryl S-200 high resolution resin (1.5 CV, where 1 CV = 500 mL) was packed into an XK 26/100 column (Cytiva, Little Chalfont, UK) using equilibration buffer (5 mL/min) on an ÄKTA fast protein liquid chromatography (FPLC) machine (UPC-900 monitor and P-90 pump; Cytiva, Little Chalfont, UK). The proteinase fractions were thawed from −20 °C, pooled and concentrated to ∼5 mL in a Vivaspin 20 protein spin concentrator (1146 × *g*, 4 °C) before being loaded onto the column (2.0 mL/min). The purification was performed with equilibration buffer (2.0 mL/min) and progress monitored at a wavelength of 280 nm using Unicorn 5.2 software (Cytiva, Little Chalfont, UK). Fractions (10 mL) were collected and frozen at −20 °C for further analysis by gelatin zymography when UV readings were recorded at 0.29 CVs (2.0 mL/min) and 0.53 CVs (2.0 mL/min). After 1.2 CVs had passed through, no further UV signals were recorded, and the purification was stopped.

### SDS-PAGE

This was carried out using the Laemmli method.^46^ Polyacrylamide gels (containing 4% total acrylamide for the stacking gel and 15% total acrylamide for the resolving gel) were cast using an SE215 multigel caster (Hoefer, Thermo Fisher Scientific, Loughborough, UK). Loading buffer (Laemmli, 2×), consisting of 0.5 M Tris-HCl, pH 6.8, 4% (v/v) SDS, 0.005% (v/v) bromophenol blue, 20% (v/v) glycerol and 5% (v/v) β-mercaptoethanol was added to an equal volume of sample and the resultant mixture boiled at 90 °C (10 min). After centrifugation (12 519 × *g*, 10 min, RT) samples were loaded onto the gel in running buffer (1×), comprising 0.02 M Tris base, pH 8.3, 0.2 M glycine, and 3.5 mM SDS. The gel was initially run at 100 V (20 min) and then at 150 V until the tracking dye (bromophenol blue) had reached the bottom of the gel.

### Coomassie Staining

Gels were stained using a solution of 0.1% (w/v) Coomassie Brilliant Blue, 50% (v/v) methanol, 20% (v/v) glacial acetic acid, and 30% (v/v) deionized water for 20 min. Destaining was performed using 30% (v/v) methanol, 1% (v/v) formic acid and 69% (v/v) deionized water until a clear background was obtained. Images were acquired under manual capture settings using a G-box XX6 gel imager (Syngene, Cambridge, UK) and gels were dried overnight and stored long-term in gel drying film (Promega, Southampton, UK).

### Silver Staining

Gels were stained as reported by Shevchenko *et al*.^47^ All solutions were made up in deionized water unless otherwise stated. Initially, gels were fixed in 50% (v/v) methanol and 5% (v/v) AcOH at 4 °C (20 min or overnight). Sequential washing with 50% (v/v) methanol (10 min) and deionized water (10 min) was followed by the addition of 0.02% (w/v) sodium thiosulfate sensitizer (1 min). Further washing (2 × 1 min, deionized water) and incubation in cold 0.1% (w/v) silver nitrate (4 °C, 20 min or overnight) ensued. After washing with deionized water (2 × 1 min), development was performed with 2% (v/v) sodium carbonate containing 0.4% (v/v) formalin (2 × 5–7 min). After removal of this solution, development was stopped by the addition of 5% (v/v) AcOH (5 min) followed by washing with deionized water (2 × 30 min). Images were acquired and gels dried and stored as described for Coomassie staining.

### Western Blotting

Proteins on SDS-PAGE gels were transferred onto methanol-activated polyvinylidene difluoride membranes at 70 V (90 min) in a transfer tank from Peqlab Biotechnologie GmbH (VWR International, Lutterworth, UK) containing transfer buffer comprising 25 mM Tris base, 192 mM glycine and 20% (v/v) methanol in deionized water. The membranes were blocked with 5% (w/v) skimmed milk in TBST buffer consisting of 19 mM Tris base, 137 mM NaCl, 2.7 mM KCl and 0.05% (v/v) Tween 20 at room temperature (60 min) or at 4 °C overnight. This was followed by two rinses (10 s) and two washes (5 min) with TBST buffer, and incubation with a 1:1000 dilution of mouse monoclonal anti-FLAG M2 alkaline phosphatase antibody (Merck, Gillingham, UK) in 5% (w/v) skimmed milk TBST (60 min). After five washes with TBST buffer (5 min), membranes were developed with Western Blue substrate (Promega, Southampton, UK) (5–10 mL), rinsed with deionized water (5 × 10 s) and imaged under automatic capture settings using a G-box XX6 gel imager (Syngene, Cambridge, UK).

### Gelatin Zymography

This was performed according to an Abcam protocol.^48^ Polyacrylamide gels (containing 4% total acrylamide for the stacking gel and 15% total acrylamide for the resolving gel) were cast using a SE215 multigel caster (Hoefer, Thermo Fisher Scientific, Loughborough, UK) with 4 mg/mL (w/v) gelatin. SDS-PAGE was run as described earlier but under nonreducing conditions and without boiling of samples. Gels were sequentially washed with washing buffer (1 × 15 min, 1 × 45 min) comprising 50 mM Tris-HCl, pH 7.5, 5 mM CaCl_2_, 1 μM ZnCl_2_, and 2.5% (v/v) Triton X-100 in water; and incubation buffer (5 min, room temperature) containing 50 mM Tris-HCl, pH 7.5, 5 mM CaCl_2_, 1 μM ZnCl_2_, and 1% (v/v) Triton X-100 in water. After removal of the latter solution, fresh incubation buffer was added and the gels allowed to stand at room temperature to enable gelatin hydrolysis to proceed (20 h). The gels were then analyzed by Coomassie staining as described previously.

### Concentration and Long-Term Storage of Metalloproteinases

The membranes of Vivaspin 20 protein spin concentrators with a molecular weight cutoff of 3 kDa (Cytiva, Little Chalfont, UK) were wetted with TNC buffer (10 mL) and centrifuged (1146 × *g*, 10 min, RT). Fractions containing purified metalloproteinases (TS4-4, TS5-5 or MMP-2ΔC) were pooled and concentrated to lower volumes (2–20 mL, depending on the amount of purified proteinase obtained) in these Vivaspin 20 tubes (1146 × *g*, RT). The absorbance at 280 nm (*A*_280_) of each sample was measured using a Nanodrop 1000 spectrophotometer (Thermo Fisher Scientific, Loughborough, UK) and was used to determine the initial concentration of each proteinase from the equation for the Beer–Lambert−Bouguer law^49^ using molar extinction coefficients computed from the ExPASy ProtParam tool (https://web.expasy.org/protparam/) assuming unreduced cysteine residues. Each proteinase was frozen down to −80 °C for long-term storage in TNC buffer containing 10% (v/v) glycerol and 0.05% (v/v) Brij-35.

### Fluorophore Calibration Curves

Calibration curves were generated for the *ortho*-aminobenzoic acid (Abz) fluorophore in TNC buffer under six different concentrations of Brij-35 surfactant: 0%, 0.0005%, 0.005%, 0.02%, 0.05% and 5% (v/v). For 5-FAM and 7-methoxycoumarin-4-acetic acid (Mca) fluorophores, only 0.005% (v/v) Brij-35 was used. The procedure for Abz was as follows: a stock solution of Abz in DMSO (1 M) was serially diluted to 1 mM in DMSO, before further dilution to 20 μM using TNC buffer containing Brij-35. This solution was diluted in 1.5-fold increments to 0.10 μM in TNC Brij-35 buffer. Each concentration in the 20 μM–0.10 μM range (inclusive) was added to wells (50 μL) in a 96-well black microplate (Greiner bio-one, Stonehouse, UK) in triplicate. A background control consisting of TNC buffer with Brij-35 (50 μL) was also added to wells in triplicate. The relative fluorescence intensity was measured at 37 °C on a SpectraMax M5 spectrophotometer (Molecular Devices, San Jose, CA, USA) using an excitation wavelength (λ_ex_) = 300 nm and emission wavelength (λ_em_) = 430 nm. A plot of relative fluorescence units (RFU) against concentration (μM) followed by linear regression analysis with Prism 9 software (GraphPad, La Jolla, CA, USA) gave the gradient in RFU μM^–1^, which was used to convert units from RFU s^–1^ to μM s^–1^ or nM s^–1^. For 5-FAM and Mca, the fluorophore dilution series were prepared as described for Abz, except that only 0.005% (v/v) Brij-35 was used. The concentration ranges and wavelengths were: 4 μM–9.1 nM (inclusive) with λ_ex_ = 485 nm and λ_em_ = 538 nm (5-FAM) and 20 μM–0.10 μM (inclusive) with λ_ex_ = 328 nm and λ_em_ = 420 nm (Mca).

### General Information on Kinetic Studies with ADAMTSs and MMPs

All proteinase assays were performed at 37 °C in a SpectraMax M5 spectrophotometer (Molecular Devices, San Jose, USA) using a 96-well black microplate (Greiner bio-one, Stonehouse, UK) unless otherwise stated. The following substrates and parameters were used for ADAMTS and MMP proteinases: Abz-TESE↓SRGAIY-Dpa-KK-NH_2_ (20 μM final except for *k*_cat_/*K*_m_ and sensitivity studies) from GL Biochem (Shanghai, China) for TS5-5, with λ_ex_ = 300 nm and λ_em_ = 430 nm; 5-FAM-AE↓LQGRPISIAK-TAMRA (0.5 μM final) from Bachem (Bubendorf, Switzerland) for TS4-4, with λ_ex_ = 485 nm and λ_em_ = 538 nm; Mca-PLG↓L-Dpa-KK-NH_2_ (1.5 μM final) from Bachem (Bubendorf, Switzerland) for all MMPs, with λ_ex_ = 328 nm and λ_em_ = 420 nm; and library parent peptide **3** with hit substrates **18**–**26** for TS5-5, TS4-4, MMP-2Δ*C* and MMP-9ΔC, with λ_ex_ = 300 nm and λ_em_ = 430 nm. N-TIMP-3 was purified as described previously^30^ and CT-1746 was a gift from Dr. Yoshifumi Itoh (Kennedy Institute of Rheumatology, University of Oxford). All analyses were carried out using Prism 9 software (GraphPad, La Jolla, CA, USA).

### Activity Studies of ADAMTSs

To test whether active TS5-5 had been purified, three concentrations of proteinase were assayed. A solution of Abz-TESE↓SRGAIY-Dpa-KK-NH_2_ (22.22 μM) in freshly prepared TNC buffer with 0.02% (v/v) Brij-35 (180 μL) was added to a 96-well black microplate in triplicate for each proteinase concentration. A substrate-only background control was also added in triplicate (180 μL). The resultant solutions were incubated at 37 °C (30 min). TS5-5 proteinase in TNC buffer with 0.02% (v/v) Brij-35 (20 μL) was added to wells to give final proteinase concentrations of 80 nM, 40 nM and 20 nM. TNC buffer with 0.02% (v/v) Brij-35 (20 μL) was also added to background control wells. The change in RFU was measured at 37 °C for 1 h, with readings every 20 s. Following subtraction of background fluorescence intensity, linear regression analysis of the linear part of each curve was used to determine the cleavage rate, which was expressed as the mean ± SEM in RFU s^–1^. For cases where a single concentration of TS5-5 or TS4-4 was assayed, the same procedure was used but with 0.005% (v/v) Brij-35 and using the 5-FAM-AE↓LQGRPISIAK-TAMRA substrate for TS4-4 (0.56 μM). To convert cleavage rates to nM s^–1^ nM^–1^, the rate in RFU s^–1^ was divided by the final concentration of proteinase (nM), then divided by the gradient of the calibration curve for Abz or 5-FAM (expressed in RFU nM^–1^).

### Activity Studies of MMPs

A solution of Mca-PLG↓L-Dpa-KK-NH_2_ (1.67 μM) in freshly prepared TNC buffer with 0.005% (v/v) Brij-35 (180 μL) was added to a 96-well black microplate in triplicate for each MMP to be assayed. A substrate-only background control was also added in triplicate (180 μL). The resultant solutions were incubated at 37 °C (30 min). A matrix metalloproteinase (MMP-1, MMP-2ΔC, MMP-3, MMP-9ΔC, or MMP-13ΔC) was added to wells (20 μL) to give a final concentration in the 0.5 nM–50 nM range. TNC buffer with 0.005% (v/v) Brij-35 (20 μL) was also added to background control wells. The change in RFU was measured at 37 °C for 1 h, with readings every 20 s. Following subtraction of background fluorescence intensity, linear regression analysis of the linear part of each curve was used to determine the cleavage rate, which was expressed as the mean ± SEM in RFU s^–1^. To convert cleavage rates to nM s^–1^ nM^–1^, the rate was divided by the final concentration of proteinase (nM), then divided by the gradient of the calibration curve for Mca (expressed in RFU nM^–1^).

### Surfactant Activity Studies with TS5-5

A 5% (v/v) Brij-35 solution in TNC buffer was diluted with TNC to 0.05%, 0.02%, 0.005%, and 0.0005% (v/v) Brij-35 surfactant. For 5% and 0% (v/v) Brij-35, buffer and TNC alone were used, respectively. Activity assays were performed for each surfactant concentration using the method described for ADAMTSs with a single concentration of TS5-5 (20 nM). For each surfactant concentration, experiments were repeated 3–7 times for TS5-5 in triplicate or quadruplicate, with final rates expressed as the mean ± SD in nM s^−1^ nM^−1^ of proteinase.

### Active Site Titration with N-TIMP-3

A stock solution of N-TIMP-3 (5.6 μM) was thawed from −20 °C and serially diluted to 320, 160, 80, 40, 20, 10, 5, and 2.5 nM in freshly prepared TNC with 0.02% (v/v) Brij-35. Each concentration (50 μL) was added to a 96-well black microplate in triplicate. Proteinases were thawed from −20 °C or −80 °C, prepared at an initial concentration in TNC buffer with 0.02% (v/v) Brij-35, and added to each well (50 μL). The initial concentrations for TS4-4, TS5-5, and MMP-2ΔC were 10, 27, and 40 nM, respectively, and were based on *A*_280_ readings. Separate wells containing TS4-4/TS5-5/MMP-2ΔC (50 μL) with TNC containing 0.02% (v/v) Brij-35 (50 μL)—as well as those with TNC containing 0.02% (v/v) Brij-35 alone (100 μL)—were set up as positive and negative controls, respectively. The mixtures were incubated at 37 °C for 1 h. A solution (100 μL) of 5-FAM-AE↓LQGRPISIAK-TAMRA substrate for TS4-4 (1 μM) or Abz-TESE↓SRGAIY-Dpa-KK-NH_2_ substrate for TS5-5 (40 μM) or Mca-PLG↓L-Dpa-KK-NH_2_ substrate for MMP-2ΔC (3 μM) in TNC with 0.02% (v/v) Brij-35 was added to wells and the relative fluorescence intensity measured at 37 °C for 1 h with readings every 20 s. The rates from the different concentrations of N-TIMP-3 were determined by linear regression and expressed in terms of the mean percentage activity ± SEM by dividing by the rate in the absence of inhibitor. These data were then fitted to the equation for tight-binding inhibition:^50^
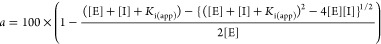
1where *a* is the percentage activity (proteolysis with inhibitor divided by proteolysis without inhibitor), [E] is the concentration of active proteinase, [I] is the concentration of the inhibitor, and *K*_i(app)_ the apparent inhibition constant of the inhibitor. Extrapolation of the linear section of the curve to the abscissa provided the concentration of active proteinase. If the initial concentration of TS4-4/TS5-5/MMP-2ΔC determined by *A*_280_ differed from that calculated by active-site titration, the assay was repeated until a consistent proteinase concentration was obtained.

### Active Site Titration with CT-1746

For titrations using CT-1746, the same procedure as for N-TIMP-3 was employed, except that the following initial proteinase concentrations were used: TS4-4/TS5-5 (480 nM), MMP-1 and MMP-9ΔC (40 nM), and MMP-3 (4 nM). For the inhibitor dilution series, either identical concentrations were used as for N-TIMP-3 or eight concentrations from the following series were used: 4 μM, 2 μM, 1 μM, 500 nM, 250 nM, 125 nM, 63 nM, 31 nM, 16 nM, 8 nM, 2 nM, and 1 nM. The choice of the inhibitor dilution series depended on the initial concentration of the ADAMTS or MMP proteinase. Titration was not performed for MMP-13ΔC as this was purchased commercially and insufficient quantities were available.

### Selectivity Studies with ADAMTSs and MMPs

Selectivity studies are described for Abz-TESE↓SRGAIY-Dpa-KK-NH_2_, the library parent peptide **3**, and hit peptides **18**–**26**. A solution of Abz-TESE↓SRGAIY-Dpa-KK-NH_2_ (22.22 μM) in freshly prepared TNC buffer with 0.005% (v/v) Brij-35 (90 μL) was added to a 96-well clear bottom microplate (Greiner bio-one, Stonehouse, UK) in triplicate. A substrate-only background control was also added in triplicate (90 μL). The resultant solutions were incubated at 37 °C (30 min). An ADAMTS or MMP proteinase was added to substrate wells (10 μL) to give a final proteinase concentration of either 50–90 nM (TS4-4), 25–200 nM (TS5-5), 5–10 nM (MMP-2ΔC), 30 nM (MMP-9ΔC), 92 nM (MMP-1), or 3 μM (MMP-3). TNC buffer with 0.005% (v/v) Brij-35 was added to substrate-only control wells (10 μL). The relative fluorescence intensity was measured at 37 °C for 1 h with readings every 20 s. Activity was read from underneath the microplate, which was covered with a low evaporation lid. However, if no cleavage was observed after 1 h, fluorescence intensity was read continuously at 37 °C for 23 h every 5 min. For each ADAMTS and MMP proteinase, the assay was performed 2–4 times. The only exception was MMP-1, where no cleavage was observed from a single experiment at a final concentration of 92 nM after 72 h. Final rates were expressed as a percentage of substrate cleavage by TS5-5 (mean ± SEM) or in μM s^–1^ μM^–1^ of proteinase.

### Determination of Kinetic Parameters (*k*_cat_ and *K*_m_) for Substrate Cleavage by TS5-5

A solution of Abz-TESE↓SRGAIY-Dpa-KK-NH_2_/**3**/**26** (5 mM) or **19**/**23** (1 mM) in DMSO underwent a 2-fold serial dilution with freshly prepared TNC buffer containing 0.005% (v/v) Brij-35. This gave initial concentrations in the following range: 222.22 to 0.87 μM (Abz-TESE↓SRGAIY-Dpa-KK-NH_2_/**3**/**23**/**26**) and 177.78 to 1.39 μM (**19**). Eight substrate concentrations (180 μL) within these ranges (including outer numbers) were added to a 96-well black microplate in triplicate and incubated at 37 °C (20 min). TNC buffer with 0.005% (v/v) Brij-35 was added to substrate-only control wells in a single replicate (180 μL). TS5-5 proteinase was thawed from −80 or −20 °C, made up in TNC buffer with 0.005% (v/v) Brij-35 (300 nM) and added to substrate wells (20 μL). TNC buffer was added to substrate-only control wells (20 μL). Fluorescence intensity was measured at 37 °C for 1 h with readings every 20 s. The rate of substrate cleavage was determined at each substrate concentration and expressed as the mean ± SEM in μM s^–1^. The subsequent data was fitted to the Michaelis–Menten eq 2:
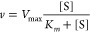
2where *v* is the rate of the reaction, *V*_max_ is the maximal rate of the reaction, *K*_m_ is the Michaelis constant, and [S] is the substrate concentration. This equation enabled the *K*_m_ value to be determined. The catalytic rate constant *k*_cat_ was determined from eq 3:
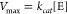
3where [E] is the concentration of proteinase, and *k*_cat_ and *V*_max_ are as defined previously. These initial *K*_m_ and *k*_cat_ parameters were determined from *n* = 3–4 independent experiments with final values for these parameters reported after a correction for the inner-filter effect.

### Correcting Kinetic Parameters for the Inner-Filter Effect

This was performed based on the protocol described in Liu *et al.*(40) to correct for quenching of the released Abz fluorophore by uncleaved substrate. Two sets of initial substrate concentrations were prepared as described in the previous section. Both sets of substrate concentrations (180 μL) were added to a 96-well black microplate in triplicate. Two buffer-alone controls with TNC buffer containing 0.005% (v/v) Brij-35 (180 μL) were also added in triplicate. All solutions were incubated at 37 °C (20 min). Abz fluorophore (1 M) in DMSO was serially diluted to 1 mM in DMSO, before further dilution to 100 μM using TNC buffer containing 0.005% (v/v) Brij-35. TNC buffer containing 0.005% (v/v) Brij-35 (20 μL) was added to the first set of substrate concentrations as well as to the first buffer-alone control. Abz fluorophore (20 μL, 100 μM) was added to the second set of substrate concentrations as well as the second buffer-alone control. The end-point fluorescence intensity was measured at 37 °C using λ_ex_ = 300 nm and λ_em_ = 430 nm. At each substrate concentration, the RFU with Abz fluorophore (RFU + Abz) was subtracted from the RFU in the absence of Abz fluorophore (RFU) to give the actual fluorescence of the Abz fluorophore (RFU_Abz_). These values were divided by RFU_Abz_ at a substrate concentration of 0 μM to give the correction factor for each concentration. The rates for each substrate concentration described in the previous section were divided by their respective correction factors (Tables S6–S10) to deliver the inner-filter corrected *k*_cat_ and *K*_m_ values.

### Evaluating the Rate of Cleavage of Parent Peptide **3** and Hit Peptide **26** by TS5-5 at Different Substrate Concentrations

This was carried out according to the procedure described for activity studies of ADAMTSs with a single concentration of proteinase, except that a range of substrate concentrations was used for **3** and **26** (20 to 0.63 μM). Additionally, control wells for each substrate concentration were used in single replicate. The final concentration of TS5-5 was 57 nM.

### Sensitivity Studies with TS5-5

Abz-TESE↓SRGAIY-Dpa-KK-NH_2_, library parent peptide **3** and hit peptide **26** (5 mM) were each diluted to two concentrations (5.56 μM and 2.78 μM) in freshly prepared TNC buffer with 0.005% (v/v) Brij-35. The two concentrations for each substrate (90 μL) were added to a 96-well black microplate in triplicate. A substrate-only background control was also added in triplicate (90 μL). The resultant solutions were incubated at 37 °C (20 min). TS5-5 proteinase was thawed from −20 °C and made up at the following concentrations in TNC buffer with 0.005% (v/v) Brij-35:10, 5, 2.5, and 1.25 nM. Each concentration of proteinase was added to substrate wells (10 μL), with buffer added to substrate-only control wells (10 μL). The relative fluorescence intensity was measured at 37 °C for 24 h, with readings every 2 min.

### Modeling Peptide Substrates into the Active Site of ADAMTS-5

The crystal structure of the combined catalytic and disintegrin domain of ADAMTS-5 (*i*.*e*. TS5-5, residues 264–555)^51^ was retrieved from the RSC Protein Data Bank (2RJQ, www.rcsb.org) and analyzed by Molecular Operating Environment software version 2018.12 (Chemical Computing Group, Montreal, Canada) on a workstation equipped with an intel i7 processor. Initially, the current substrate Abz-TESE↓SRGAIY-Dpa-KK-NH_2_ was constructed using the builder tool, partial charges corrected using the Protonate 3D tool (<5 min) and the lowest energy conformation obtained through energy minimization using the Amber12:EHT force field with a gradient of 0.1 (30 min). The sequence was docked into the active site of TS5-5 and anchored in position by restraining the distance between the carbonyl oxygen of Glu in P1 and the catalytic Zn^2+^ ion with a lower penalty boundary of 2 pm and an upper penalty boundary of 3 pm. The substrate-TS5-5 complex was then solvated using a spherical droplet solvation shell of water with a margin of 4.0. The overall structure was minimized as before (30 min) and molecular dynamics simulations performed with rigid water molecules (24 h, 300 K, 2 fs step-length, 2–3 ns calculation length). These occurred without constraints on the substrate but with fixed enzyme (except for residues in direct contact with the substrate) and with constraints on the distance between Zn^2+^ and the three His residues of TS5-5. This gave the starting point for the design process. From here, the initial P4′ fluorophore substrate Y(NO_2_)TESESRGK(Abz)IYYKKG was modeled with a single change made at a time. For each change, a charge correction was performed (<5 min), followed by an energy minimization (30 min) and molecular dynamics simulations using the same parameters as before. Amino acids at specific positions were mutated directly to the desired residue using the protein builder tool. The NO_2_ and Abz moieties were introduced into the substrate by generating the structure through the builder tool and “stitching” this on through bond formation. Both parent compounds for the P7′ and P9′ fluorophore substrates were modeled from the parent P4′ structure. For the P4′, P7′ and P9′ fluorophore series shown in Tables S2–S4, substrates were modeled starting from the relevant parent compound each time using the same general procedure described for the parent P4′ substrate.

### General Information for Combinatorial Chemistry and Chemical Synthesis

No unexpected, new, or significant safety hazards or risks were associated with this work, which was performed at the Center for Evolutionary Chemical Biology (CECB) at the University of Copenhagen, Denmark. All reagents were obtained from commercial suppliers and used as received. The only exception was PEGA_1900_ resin, which came from the now defunct VersaMatrix A/S. Peptides were synthesized in a custom 20-well Teflon synthesis block^52^ using Fmoc-SPPS or split-mix combinatorial synthesis coupled with Fmoc-SPPS. DMF and 20% (v/v) piperidine/DMF were dispensed into the Teflon synthesis block using a bottle-top dispenser from Socorex Isba S.A. Shaking was carried out using an IKA-Vibrax-VXR or IKA KS 130 basic and vortexing with an IKA MS2 minishaker. A strip heater was used to heat the IKA KS 130 basic shaker during the Kaiser test. TELOS SPE filtration columns with a polyethylene frit (20 μm pore size) were used for amino acid couplings outside of the Teflon synthesis block. Buffers were adjusted to the correct pH using a VWR pHenomenal pH 1100L pH meter with distilled water from a Siemens Ultra-Clear TWF water purification system. Thin layer chromatography was performed using 60 F_254_ silica plates from Merck and analyzed under a UV lamp (254 nm) from Konrad Benda. Analytical liquid chromatography runs (13 min) were performed on an Agilent 1100 series HPLC using a Waters X-Bridge C18 5 μm column (4.6 × 100 mm) and a solvent system of 90% acetonitrile (MeCN)/water + 0.1% TFA and 100% water + 0.1% TFA. After 10-fold sample dilution, peptide mass was confirmed by an analytical run (5 min) on a Waters Acquity H-Class UPLC with a XEVO G2-S QTof Zspray mass spectrometer (ESI-MS) using a BEH C-18 1.7 μm column (2.1 × 50 mm) and a solvent system of water + 0.1% formic acid (FA) and MeCN + 0.1% FA. MALDI and tandem mass spectrometry were carried out on a Bruker solariX XR instrument, with data analyzed using Bruker Compass DataAnalysis 5.1. ^1^H NMR spectroscopy was performed on a Bruker BioSpin at 400 MHz. Chemical shifts are referenced to residual solvent and reported in ppm with coupling constants listed in hertz (Hz).

### Synthesis of Fmoc-Lys(*retro*-Abz-Boc)-OH

To a solution of *tert*-butyloxycarbonyl (Boc)-Abz-OH (3.1 g, 13 mmol., 1.2 equiv), (1-cyano-2-ethoxy-2-oxoethylidenaminooxy)dimethylamino-morpholino-carbenium hexafluorophosphate (COMU, 5.6 g, 13 mmol., 1.2 equiv) in DMF (94 mL) was added *N*,*N*-diisopropylethylamine (DIPEA, 4.6 mL, 27 mmol., 2.5 equiv), and the subsequent solution stirred at room temperature (30 min). Fmoc-l-lysine (4.0 g, 11 mmol., 1.0 equiv) was added, and the subsequent cloudy orange solution stirred overnight (21 h). HPLC and UHPLC-MS of the reaction mixture revealed the presence of product mass. Gravity filtration of the reaction mixture was followed by the addition of ethyl acetate (EtOAc, 100 mL) and brine (200 mL). The aqueous layer was extracted with EtOAc (3 × 100 mL), and the combined organic layers washed sequentially with saturated NaCl, 1 M H_2_SO_4_, 5% (w/v) sodium hydrogen carbonate (NaHCO_3_), 1 M sulfuric acid, and water (2 × 100 mL each). Drying with sodium sulfate, filtration, and concentration *in vacuo* afforded a pale yellow solid. This was purified using silica gel column chromatography using a gradient system of 1–3% MeOH/dichloromethane (DCM) and dried under high vacuum to deliver the title compound as an off-white solid with a purity ≥ 99% by HPLC (1.21 g, 19%), which was used in the synthesis of ADAMTS-5 substrates. *R*_f_ = 0.2 (9:1 DCM/MeOH); ^1^H NMR (400 MHz, CDCl_3_): δ 10.00 (s, 1H, OH), 8.16 (d, *J* = 8.4 Hz, 1H, ArH), 8.10–7.72 (m, 1H, NHCOO), 7.62 (d, *J* = 7.5 Hz, 2H, ArH), 7.43 (dd, *J* = 7.7, 3.9 Hz, 2H, ArH), 7.33–7.20 (m, 4H, ArH), 7.16 (t, *J* = 7.8 Hz, 2H, ArH), 6.75 (t, *J* = 7.6 Hz, 1H, ArH), 6.73–6.63 (m, 1H, CH_2_NHCO), 5.70–5.61 (m, 1H, OCONH), 4.27–4.15 (m, 3H, CH_2_O, Lys H_α_), 4.04 (t, *J* = 7.2 Hz, 1H, CHCH_2_O), 3.30–3.16 (m, 2H, Lys H_ε_), 1.82–1.69 (m, 1H, Lys H_β_), 1.65–1.53 (m, 1H, Lys H_β_), 1.52–1.45 (m, 2H, Lys H_δ_), 1.40 (s, 9H, CH_3_), 1.35–1.23 (m, 2H, Lys H_γ_) ppm. LRMS: LC-MS (ESI) *m*/*z* 588.1 [M + H]^+^.

### Linear Synthesis of Modeled Peptide Substrates on PEGA_1900_ Resin

PEGA_1900_ resin (8.4 g, 1.0 mmol., 0.12 mmol/g loading) was swelled in DMF (14 mL) at room temperature (10 min) in a TELOS column, and solvent removed. Treatment with 20% (v/v) piperidine/DMF (14 mL, 20 min) was followed by washing with DMF (5 × 14 mL). To Fmoc-Gly-OH (1.2 g, 4.0 mmol., 4.0 equiv) in DMF (14 mL) was added 4-ethylmorpholine (NEM, 497 μL, 3.9 mmol., 3.9 equiv), and the subsequent solution allowed to stand (≥1 min) at room temperature. After the addition of *N*,*N*,*N′*,*N′*-tetramethyl-*O*-(benzotriazol-1-yl)uronium tetrafluoroborate (TBTU, 1.3 g, 4.0 mmol., 4.0 equiv), the mixture was added to the resin, shaken, and allowed to stand at room temperature (1.5 h). Resin was washed (DMF, 4 × 14 mL) and deprotection performed with 20% (v/v) piperidine/DMF (14 mL, 20 min). Coupling of the 4-hydroxymethylbenzoic acid linker (HMBA, 0.61 g, 4.0 mmol., 4.0 equiv) was then carried out as described for Fmoc-Gly-OH (2 h). After washing with DMF (4 × 14 mL), the resin was sequentially washed with dry MeCN and dry DCM (both 5 × 14 mL). The first amino acid of the peptide sequence, Fmoc-Gly-OH (1.2 g, 4.0 mmol., 4.0 equiv), was coupled in dry DCM (14 mL) using 1-methylimidazole (1-MeIm, 370 μL, 4.6 mmol., 4.6 equiv) and 1-(mesitylene-2-sulfonyl)-3-nitro-1*H*-1,2,4-triazole (MSNT, 1.2 g, 4.0 mmol., 4.0 equiv). After 1 h, resin was washed with dry DCM (4 × 14 mL) and the coupling step repeated a second time. This was followed by deprotection with 20% (v/v) piperidine/DMF (5 min) and washing with DMF (4 × 14 mL). The resin was transferred to the custom 20-well Teflon synthesis block and evenly redistributed across wells with DMF (14 mL). Amino acids were then coupled with TBTU for 1 h up to the first six residues, after which couplings were performed for 2 h using the same conditions and equivalents as for Fmoc-Gly-OH (0.05 mmol. scale/well). The only exceptions were noncanonical amino acids (which were all coupled for 3 h) and Fmoc-Lys(*retro*-Abz-Boc)–OH, which was coupled using 3.0 equiv of amino acid, 3.0 equiv of TBTU and 3.9 equiv of NEM. Completion of coupling reactions and successful deprotections were confirmed using the Kaiser test^53^ with unmodified PEGA_1900_ resin acting as a positive control. Final Fmoc deprotection was carried out twice using 20% (v/v) piperidine/DMF (14 mL, 1 × 2 min and 1 × 20 min) followed by washing with DMF (6 × 14 mL) and drying under low vacuum (0.5 h). Protecting groups were removed using a cleavage cocktail consisting of 95% TFA: 2.5% triisopropylsilane: 2.5% water (14 mL). After initial cleavage (10 min), solvent was removed and a second cleavage performed under the same conditions (14 mL, 3 h). After removal of the second cleavage cocktail, the resin was sequentially washed with DCM (2 × 14 mL), DMF (2 × 14 mL), 5% (v/v) DIPEA/DMF (5 × 14 mL), DMF (5 × 14 mL), DCM (5 × 14 mL), Milli-Q water (3 × 14 mL), and TNC buffer (3 × 14 mL), dried under low vacuum (1 h), and air-dried overnight. The resultant resin-bound peptides were taken forward to test for cleavage by TS5-5.

### Kaiser Test

To a small number of resin beads (<1 mg) was sequentially added 2–3 drops of phenol: ethanol (80:20, w/v), 1 mM potassium cyanide: pyridine (2:98, v/v) and 5% (w/v) ninhydrin in ethanol. The beads were heated to 110–120 °C and the color change monitored visually. The presence of free amine groups was indicated by dark blue beads, whereas clear beads indicated that successful coupling had occurred.^53^

### Cleavage of Modeled Peptide Substrates from PEGA_1900_ Resin

To release peptide substrates from PEGA_1900_ resin (∼1 mg), the base-labile HMBA linker was cleaved using 0.1 M NaOH (1 mL, 1 h). This solution was transferred to a TELOS column containing 0.1 M HCl (1 mL). The subsequent neutralized solution was diluted 65-fold with Milli-Q water or MeCN and an analytical UHPLC run performed to determine peptide purity. Accurate peptide mass was then ascertained *via* MALDI-Time-Of-Flight Mass Spectrometry (MALDI-TOF-MS).

### MALDI-TOF-MS of Modeled Peptide Substrates

A solution of α-cyano-4-hydroxycinnamic acid (1 μL, 10 mg/mL) in 70% (v/v) MeCN/water was spotted onto a MTP 384 polished steel target plate (Bruker, Coventry, UK). Peptide dissolved in water/MeCN (1 μL) from the UHPLC analytical sample was also added. After sample crystallization, the plate was read in a Bruker solariX XR instrument and data analyzed using Bruker Compass DataAnalysis 5.1.

### On-Resin Screening of Modeled Peptide Substrate against TS5-5

For each peptide substrate to be assayed, TNC buffer containing 0.005% (v/v) Brij-35 (50 μL) was added to wells in a 96-well glass-bottomed black microplate (PerkinElmer, Seer Green, UK) in single replicate. A background control well with the same volume was also prepared. PEGA_1900_ beads containing a bound peptide substrate were added to wells, with unbound PEGA_1900_ starting material beads added to the control well (between 10–30 beads were used in each case). All wells were incubated at 37 °C (15 min). The initial fluorescence intensity of one bead from each substrate was measured using an IX73 inverted fluorescence microscope with an XC10 magnifier (Olympus, Southend-on-Sea, UK) under UPlanSAPO 10× magnification (30 ms exposure) and subtracted from the fluorescence intensity of the background solution. The beads were then imaged using cellSens dimension 1.13 software (Olympus, Southend-on-Sea, UK). After further incubation (37 °C, 15–30 min), TS5-5 (50 μL, 1 μM) in TNC buffer with 0.005% (v/v) Brij-35 was dispensed into all wells to give a final concentration of 500 nM. The microplate was incubated again at 37 °C and the fluorescence intensity of the beads measured at 1 h and 24 h time points as before.

### Synthesis of a Combinatorial Library on PEGA_1900_ Resin

To enable the rapid synthesis of a combinatorial library of peptide substrates using the split-mix method, a custom-made 20-well Teflon synthesis block was used.^52^ Initially, however, uniformly sized PEGA_1900_ resin beads in water (18 mL)^54^ were swollen in a TELOS column and solvent removed. The resin beads were weighed (8.28 g, 1.0 mmol., 0.1 mmol/g loading), washed (4 × 20 mL) and swelled in DMF (10 min, 20 mL). After removal of solvent, the resin was deprotected using 20% (v/v) piperidine/DMF (20 mL, 20 min) and washed with DMF (5 × 20 mL). Synthesis was then performed using the same method as described for linear synthesis of the modeled peptide substrates but with the following differences: (i) the library was mixed through vigorous manual shaking (2 × 20 s) after the completion of each coupling step (and prior to Fmoc-deprotection) to ensure that each well contained a mixture of all coupled amino acids; and (ii) the coupling of Fmoc-Lys(*retro*-Abz-Boc)-OH was performed overnight using 3.0 equiv of amino acid, 3.0 equiv of TBTU and 2.93 equiv of NEM in DMF (14 mL). Removal of N-terminal and side chain protecting groups and subsequent washing steps were performed as described for the linear synthesis. The library was dried under low vacuum (2 h), transferred out of the 20-well Teflon synthesis block into a TELOS column using water and stored at 4 °C overnight.

### Incubation of the Combinatorial Library with TS5-5 and Isolation of Hits

The combinatorial library was suspended in TNC Brij-35 0.005% (v/v) (31 mL) and beads dispersed through gentle mixing and manual shaking. Library beads (1 mL) were then evenly distributed throughout 31 individual wells across Corning 6-well tissue culture plates (Thermo Fisher Scientific, Loughborough, UK) to give a single layer of ∼3500 beads/well. A well containing unbound PEGA_1900_ starting material beads (1 mL) was used as a negative control. After incubation of the library at 37 °C (30 min), TS5-5 proteinase in TNC Brij-35 0.005% (v/v) was added to each well (1 mL, 1 μM) to give a final concentration of 500 nM and the plates incubated at 37 °C. Library beads were analyzed for signs of fluorescence every 30 min. After 1 h, fluorescence was observed for ∼80 beads across all wells. Library beads were therefore transferred back into a TELOS column and washed with water (2 × 20 mL). TS5-5 proteinase was deactivated with 2% (v/v) TFA in water (2 × 20 mL), the beads washed (water, 2 × 20 mL) and basified with 2% (w/v) NaHCO_3_ (2 × 20 mL). The beads were then washed a final time with water (3 × 20 mL) prior to being dried under low vacuum (30 min) and left overnight at 4 °C. The library was resuspended in water (20 mL) and transferred back to 6-well tissue culture plates (1 mL/well). Beads were analyzed under a fluorescent microscope using the same settings as described for screening of the modeled peptide substrates. Twenty fluorescent beads were removed manually using a pipet tip (1 mL) and peptides were cleaved from PEGA_1900_ resin as described in the next section.

### Cleavage of Hit Peptide Substrates from PEGA_1900_ Resin

Twenty fluorescent beads were transferred to individual Kinesis LC-MS vials (Cole-Palmer, St. Neots, UK), and each bead treated with a cleavage solution of 5% (v/v) triethylamine (TEA) in water (50 μL, 2.5 h). To evaporate off the cleavage solution, vials were left at room temperature overnight in a fumehood set to an increased flow rate. The beads were then washed with 70% (v/v) MeCN/water (3 × 20 μL). An aliquot of each solution was taken (1 μL) and the sample prepared for MALDI as described for the modeled peptide substrates.

### MALDI and Tandem Mass Spectrometry of Hit Peptide Substrates

The MTP 384 polished steel target plate containing hit substrate samples from library screening was read on a Bruker solariX XR instrument. An initial MALDI spectrum was obtained in the 150–2500 *m*/*z* range using the following parameters: a laser frequency = 200 Hz and collision energy = 14.0 V combined with a raster smart walk pattern. This provided the mass of the quasi-molecular ion; namely the singly protonated intact peptide [M + H]^+^. Using collision-induced dissociation (CID), the quasi-molecular ion was fragmented with a laser frequency = 200 Hz, collision cell radiofrequency (RF) = 1.6 Vpp and collision energy = 80.0 V using a raster smart walk pattern. This generated a tandem mass spectrum also in the 150–2500 *m*/*z* range. The data for each hit peptide was collected and analyzed using Bruker Compass DataAnalysis 5.1.

### Analysis of Mass Spectra with Computational Software

Bruker Compass DataAnalysis was used to analyze both MALDI and tandem mass spectra, while LibMSCalc was used to score each peptide hit.^39^ Bruker Compass DataAnalysis established the mass of the quasi-molecular ion from the initial MALDI mass spectrum; [M + H]^+^. A subsequent tandem mass spectrum of the quasi-molecular ion (or a closely related high molecular mass ion) generated a y-ion or b-ion series. To identify the ion fragments within each series, a database of amino acids was generated. Natural amino acids were already present, but un-natural amino acids (*e*.*g*., β-cyclopropyl-alanine) had to be entered manually by residue mass. Amino acids that were not used in combinatorial library synthesis were removed from the database. A tool was then used to identify these fragment ions by assigning residues to the difference in mass between fragment ion peaks. As the amino acids used in the combinatorial library at each position were known, the residues lost from the fragment ion peaks were pieced together to generate an overall fragmentation pattern, which enabled the determination of the final peptide sequence.

Thereon, LibMSCalc was used to “score” each of the determined sequences. The amino acid building blocks of the combinatorial library were entered into the software with the mass of the quasi-molecular ion. The output from the software was an exhaustive list of all possible peptide sequences that matched the mass of the quasi-molecular ion. In addition, a computed mass list of fragment ions with masses and sequences for each of the possible peptides was also listed. An experimental mass list of fragment ions compiled from the tandem mass spectrum of each peptide hit was imported into LibMSCalc. The software matched the experimental mass list to the computed mass list and provided a score for each peptide based on the number of matching fragment ion peaks and their intensity. The higher the score, the higher the likelihood that there was only one peptide sequence that could match the data. Out of the hits that could be conclusively identified, the sequences listed with the highest score were synthesized by Fmoc-SPPS, and purified and characterized by LC-MS. The synthesis, purification and characterization were carried out by GL Biochem (Shanghai, China). All peptides were ≥95% purity by HPLC.

### Determination of *n*-Octanol/Water Partition Coefficients (cLog*P* Values)

These were calculated using ACD/ChemSketch 2022.1.0 (Advanced Chemistry Development, Toronto, Canada).

## Supporting Information Available

Peptide SMILES strings and kinetic data (CSV)
Additional kinetic data, a table of known ADAMTS-5 substrates, tables of FRET substrates with modeled snapshots, peptide characterization data, *n*-octanol/water partition coefficients and tandem mass spectra (PDF)

## Abbreviations

λ_em_: emission wavelength
λ_ex_: excitation wavelength
ΔC: catalytic domain
ΔFI: mean fluorescence intensity
5-FAM: 5-carboxyfluorescein
*A*_280_: absorbance at 280 nm
Abz: *ortho*-aminobenzoyl/*ortho*-aminobenzoic acid
ADAMTS-4/5: a disintegrin and metalloproteinase with thrombospondin type I motifs-4/5
B: 2,4-diaminobutyric acid
BlueF: blue fluorophore
cLog*P*: calculated Log*P*
CV: column volume
DIPEA: *N*,*N*-diisopropylethylamine
DMEM: Dulbecco’s modified Eagle’s medium
Dpa: *N*-3-[2,4-dinitrophenyl]-l-2,3-diaminopropionyl
EtOAc: ethyl acetate
FBS: fetal bovine serum
Fmoc-SPPS: Fmoc-based solid-phase peptide synthesis
FRET: Förster resonance energy transfer
HMBA: 4-hydroxymethylbenzoic acid
J: β-cyclopropyl-alanine
*k*_cat_: catalytic rate constant
Mca: 7-methoxycoumarin-4-acetic acid/7-methoxycoumarin-4-yl-acetyl
MeCN: acetonitrile
NaHCO_3_: sodium hydrogen carbonate
NEM: 4-Ethylmorpholine
N-TIMP-3: N-terminal domain of the endogenous inhibitor tissue inhibitor of metalloproteinases-3
O: ornithine
OA: osteoarthritis
PEGA: poly(ethylene glycol)-polydimethyl acrylamide
PS: penicillin-streptomycin
Q: quencher
RFU: relative fluorescence units
SD: standard deviation
SEM: standard error of the mean
TAMRA: *N*,*N*,*N′*,*N′*-tetramethyl-6-carboxyrhodamine
TBTU: *N*,*N*,*N′*,*N′*-tetramethyl-*O*-(benzotriazol-1-yl)uronium tetrafluoroborate
TNC: 50 mM Tris-HCl, pH 7.5, 150 mM NaCl, 10 mM CaCl_2_ and 0.02% (w/v) NaN_3_
U: 2,3-diaminopropionic acid
X: 4-hydroxyproline
Y(NO_2_): 3-nitro-l-tyrosine
Z: 4-thiazolyl-alanine
