# Supplementary material for: Development of Selective ADAMTS-5 Peptide Substrates to Monitor Proteinase Activity
Source: J Med Chem. 2023 Feb 22;66(5):3522–39. doi: 10.1021/acs.jmedchem.2c02090 (PMC10009750; doi:10.1021/acs.jmedchem.2c02090)
Supplement: Supplementary file 2 — jm2c02090_si_002.pdf [file jm2c02090_si_002.pdf]

Supporting information

Development of Selective ADAMTS-5 Peptide Substrates to Monitor Proteinase Activity

Milan M. Fowkes,[1]* Linda Troeberg,[2] Paul E. Brennan,[3] Tonia L. Vincent,[1] Morten Meldal[4] and Ngee H. Lim[1]

[1] Centre for OA Pathogenesis Versus Arthritis, Kennedy Institute of Rheumatology, University of Oxford, Roosevelt Drive, Headington, Oxford, OX3 7FY, United Kingdom

[2] Norwich Medical School, Bob Champion Research and Education Building, Rosalind Franklin Road, University of East Anglia, Norwich, NR4 7UQ, United Kingdom

[3] Alzheimer's Research UK Oxford Drug Discovery Institute, Centre for Medicines Discovery, Nuffield Department of Medicine Research Building, University of Oxford, Old Road Campus, Headington, Oxford, OX3 7FZ, United Kingdom

[4] Department of Chemistry, University of Copenhagen, Universitetsparken 5, Building B304, Copenhagen, DK-2100, Denmark.

* **Email:** milan.fowkes@cmd.ox.ac.uk

Table of Contents

Figures and Tables **2**

HPLC characterisation data of commercially synthesised ADAMTS-5 FRET peptide substrates **19**

Figures and Tables continued **30**

Figures and Tables

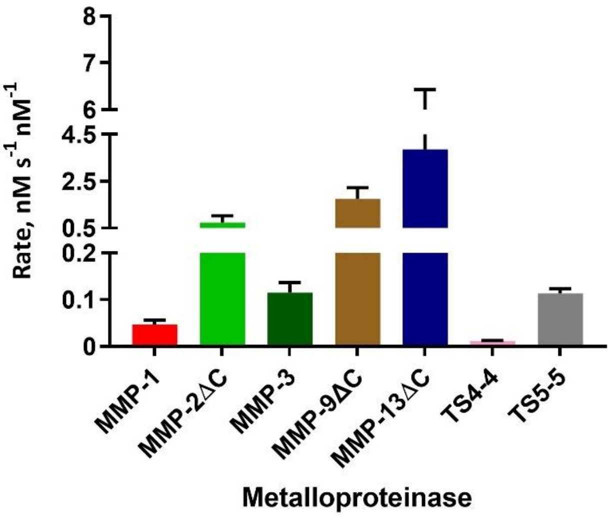

Figure S1. Activity of metalloproteinases from ADAMTS-5 selectivity studies. (*A*) Cleavage rate of 7-Methoxycoumarin-4-yl-acetyl (Mca)-PLG↓L-Dpa-AR-NH_2 (1.5 µM) by MMPs, 5-carboxyfluorescein (5-FAM)-AE↓LQGRPISIAK-*N,N,N',N'*-tetramethyl-6-carboxyrhodamine (TAMRA) (0.5 µM) by TS4-4 and Abz-TESE↓SRGAIY-Dpa-KK-NH_2 (20 µM) by TS5-5. Each rate is expressed as the mean ± SEM for 2-8 independent experiments performed in triplicate at 37 °C. Rates were determined in nM s^{-1} nM^{-1} using calibration curves for Mca, 5-FAM and Abz fluorophores at 0.005% (v/v) Brij-35 surfactant. ΔC = catalytic domain, TS4-4, TS5-5 = catalytic and disintegrin domains, MMP-1 and MMP-3 = full length.

Table S1. Known peptide substrates cleaved by ADAMTS-5 compiled from CutDB, BRENDA and MEROPS online databases.

P4	P3	P2	P1	P1'†	P2'	P3'	P4'	P5'	P6'	P7'	P8'	P9'
R	I	P	E	G	A	T	H	I	K	V	R	Q
T	E	G	E	A	R	G	S	V	I	Dpa	K	K
G	E	L	E	G	R	G	T	I	D	I	S	G
S	E	L	E	G	R	G	T	I	G	I	S	G
K	E	E	E	G	L	G	S	V	E	L	S	G
I	S	Q	E	L	G	Q	R	P	P	V	T	H
T	A	Q	E	A	G	E	G	P	S	G	I	L
T	E	G	E	A	R	G	S	V	I	L	T	V
S	D	V	M	G	R	G	H	A	R	L	V	H
D	V	Q	E	F	R	G	V	T	A	V	I	R
T	K(Mca)	G	E	L	E	G	R	G	T	K(Dnp)	G	I
K(Mca)	G	E	L	E	G	R	G	T	K(Dnp)	G	I	S
G	E	L	E	G	R	G	T	K(Dnp)	G	I	S	G
L	E	G	R	G	T	K(Dnp)	G	I	S	G	P	Hyp
K(Mca)	K	Q	E	F	R	G	Q	T	K(Dnp)-NH_2	-	-	-
R	M	N	M	C	I	E	M	G	G	N	P	L
A	F	K	C	L	K	D	G	A	G	D	V	A
D	A	M	S	L	D	G	G	F	V	Y	I	A
A	F	R	C	L	V	E	K	G	D	V	A	K

P4	P3	P2	P1	P1'†	P2'	P3'	P4'	P5'	P6'	P7'	P8'	P9'
S	V	A	C	V	K	K	A	S	Y	L	D	C
P	L	G	E	V	V	G	E	D	P	M	A	E
P	S	R	S	F	Y	R	Q	N	G	E	P	Y
L	G	E	V	V	G	E	D	P	M	A	E	L
V	K	L	W	S	L	N	P	D	T	G	L	W
F	K	F	E	N	Q	R	R	N	K	R	E	D
K	F	E	N	Q	R	R	N	K	R	E	D	R
F	E	N	Q	R	R	N	K	R	E	D	R	T
Q	R	R	N	K	R	E	D	R	T	F	L	V
L	V	T	A	T	L	G	G	E	E	L	E	P
P	L	P	A	T	V	G	V	T	Q	P	Y	L
T	A	T	L	G	G	E	E	L	E	P	A	P
P	A	T	V	G	V	T	Q	P	Y	L	D	R
A	L	V	T	A	T	L	G	G	E	E	L	E
Q	V	L	G	D	Q	T	V	S	D	N	E	L
K	Y	V	N	K	E	I	Q	N	A	V	N	G
P	G	A	A	G	R	V	G	P	P	G	S	N
P	P	G	P	S	G	K	D	G	P	K	G	A
P	G	P	S	G	K	D	G	P	K	G	A	R
G	A	A	G	V	K	D	G	R	G	E	T	G
S	P	G	P	A	G	P	T	G	K	Q	G	D

P4	P3	P2	P1	P1'†	P2'	P3'	P4'	P5'	P6'	P7'	P8'	P9'
P	G	P	Q	G	H	A	G	A	Q	G	P	P
P	G	I	N	G	S	P	G	G	K	G	E	M
G	L	D	K	V	P	K	D	L	P	P	D	T
D	T	T	L	L	D	L	Q	N	K	I	T	E
D	V	Q	E	F	R	G	V	T	A	V	I	R
G	P	A	Y	A	Y	G	S	P	P	Q	P	E
K	T	P	F	V	T	H	P	G	Y	D	T	G
G	Q	Q	P	S	V	G	Q	Q	M	I	F	E
A	A	W	D	L	P	Q	Q	A	H	Q	P	T
P	L	E	E	S	Q	D	Q	C	K	C	E	N
T	E	S	E	S	R	G	A	I	Y	Dpa	K	K
V	A	F	H	D	F	S	S	D	L	E	N	V
N	V	P	H	L	Y	R	L	R	L	D	G	N
G	S	Q	C	S	V	D	L	E	S	A	S	G
S	F	Q	E	H	T	V	D	G	E	N	Q	I
D	L	E	S	A	S	G	E	K	D	L	A	P
E	A	A	E	A	R	R	G	Q	F	E	S	V

C = carboxymethylated cysteine; Mca = 7-hydroxy-4-methylcoumarin; Dnp = 2,4-dinitrophenyl; Hyp = 4-hydroxyproline; Dpa = *N*-3-[2,4-dinitrophenyl]-2,3-diaminopropionyl.

†The cleavage site for each peptide sequence is located between the two residues coloured in red.

The order of the substrates in this table is arbitrary.

Table S2. Summary of FRET peptide substrates containing a P4ʹ fluorophore docked into the active site of the crystal structure of ADAMTS-5.

Compound	Peptide sequence	Snapshot of modification (MOE)
1	Y(NO$_2$)TESESRG**K(Abz)**IYYKKG	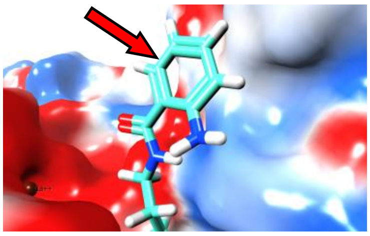
2	Y(NO$_2$)TE**G**ESRGK(Abz)IYYKKG	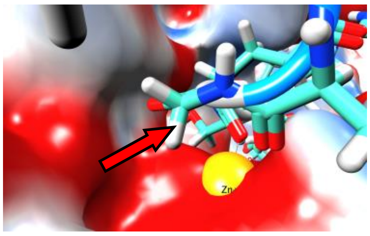
3	**K**Y(NO$_2$)TESESRGK(Abz)IYYKKG	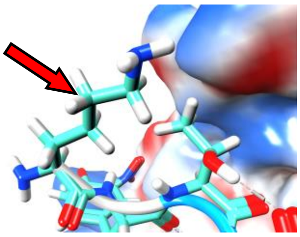
4	Y(NO$_2$)TESESRGK(Abz)**J**YYKKG	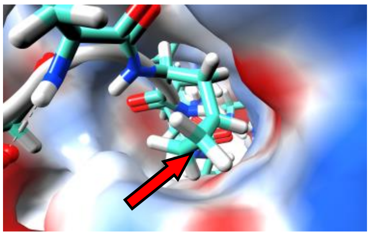
5	Y(NO$_2$)**P**ESESRGK(Abz)IYYKKG	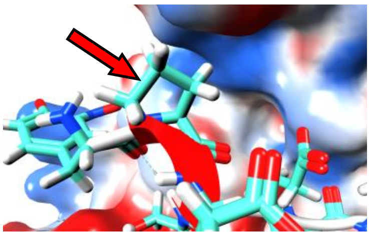
6	Y(NO$_2$)**X**ESESRGK(Abz) IYYKKG	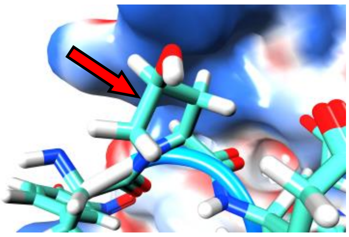
7	Y(NO$_2$)**TG**TESESRGK(Abz)IYYKKG	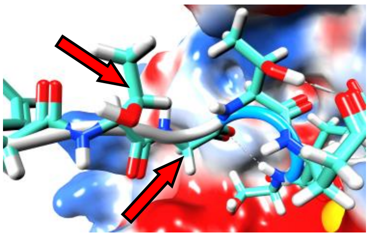

Key: J = β-cyclopropyl-alanine; X = 4-Hydroxyproline. Residues altered within each fluorophore series are coloured in red in the sequence and indicated by a red arrow in the snapshot.

Table S3. Summary of FRET peptide substrates containing a P7′ fluorophore docked into the active site of the crystal structure of ADAMTS-5.

Compound	Peptide sequence	Snapshot of change (MOE)
8	Y(NO_2)TESESRGAIY**K(Abz)**KKG	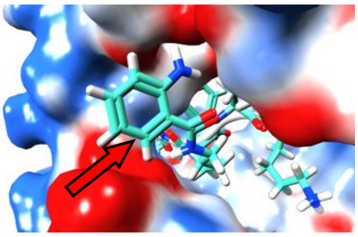
9	Y(NO_2)TESESRGAIYK(Abz)**B**KG	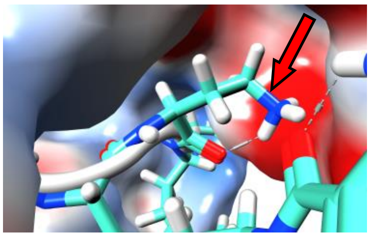
10	Y(NO_2)TESESRG**Z**IYK(Abz)KKG	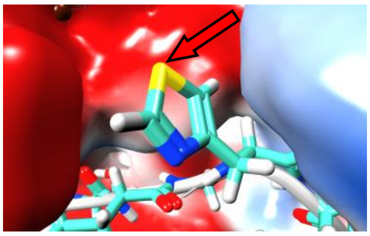
11	Y(NO_2)**P**ESESRGAIYK(Abz)KKG	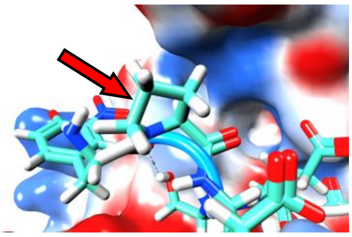
12	Y(NO_2)TE**G**ESRGAIYK(Abz)KKG	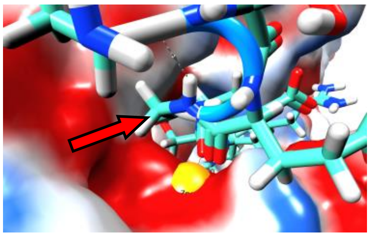

Key: B = 2,4-Diaminobutyric acid; Z = 4-Thiazolyl-alanine. Residues altered within each fluorophore series are coloured in red in the sequence and indicated by a red arrow in the snapshot.

Table S4. Summary of FRET peptide substrates containing a P9′ fluorophore docked into the active site of the crystal structure of ADAMTS-5.

Compound	Peptide sequence	Snapshot of change (MOE)
13	Y(NO$_2$)TESESRGAIYYK**K(Abz)**G	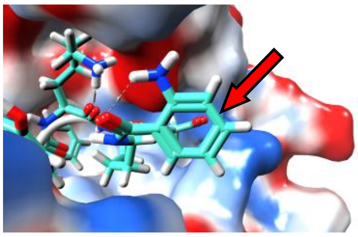
14	Y(NO$_2$)**P**ESESRGAIYYKK(Abz)G	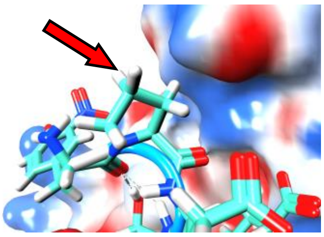
15	Y(NO$_2$)TESESRGAIY**L**KK(Abz)G	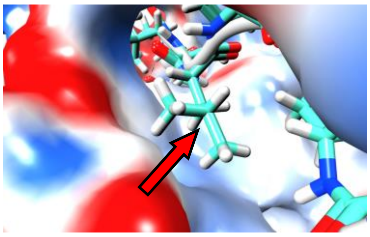
16	Y(NO$_2$)TESESRGAIY**J**KK(Abz)G	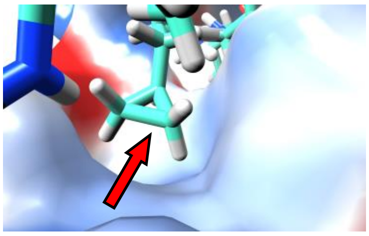
17	Y(NO$_2$)TE**G**ESRGAIYYKK(Abz)G	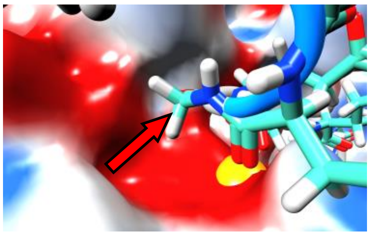

Key: J = β-cyclopropyl-alanine; Residues altered within each fluorophore series are coloured in red in the sequence and indicated by a red arrow in the snapshot.

Table S5. Summary of modelled ADAMTS-5 FRET peptide substrates synthesised by manual Fmoc-SPPS and characterised by mass spectrometry.

	Compound	Peptide sequence	Crude % Purity (by UHPLC)	HRMS (calculated) $[M+H]^+$	HRMS (found) $[M+H]^+$
P4'	1	Y(NO_2)TESESRGK(Abz)IYYKKG	≥ 81	1972.9405	1972.9428
	2	Y(NO_2)TE**G**ESRGK(Abz)IYYKKG	≥ 71	1942.9299	1942.9314
	3	**K**Y(NO_2)TESESRGK(Abz)IYYKKG	≥ 76	2101.0355	2101.0378
	4	Y(NO_2)TESESRGK(Abz)**J**YYKKG	≥ 77	1970.9248	1970.9262
	5	Y(NO_2)**P**ESESRGK(Abz)IYYKKG	≥ 64	1968.9456	1968.9464
	6	Y(NO_2)**X**ESESRGK(Abz)IYYKKG	≥ 65	1984.9405	1984.9382
	7	Y(NO_2)**TG**TESESRGK(Abz)IYYKKG	≥ 72	2131.0096	2131.0080
P7'	8	Y(NO_2)TESESRGAIY**K(Abz)**KKG	≥ 78	1880.9143	1880.9150
	9	Y(NO_2)TESESRGAIYK(Abz)**B**KG	≥ 80	1852.8830	1852.8813
	10	Y(NO_2)TESESRG**Z**IYK(Abz)KKG	≥ 75	1963.8972	1963.8980
	11	Y(NO_2)**P**ESESRGAIYK(Abz)KKG	≥ 72	1876.9194	1876.9202
	12	Y(NO_2)TE**G**ESRGAIYK(Abz)KKG	≥ 76	1850.9037	1850.9057
P9'	13	Y(NO_2)TESESRGAIYYK**K(Abz)**G	≥ 78	1915.8826	1915.8854
	14	Y(NO_2)**P**ESESRGAIYYKK(Abz)G	≥ 66	1911.8877	1911.8853
	15	Y(NO_2)TESESRGAIY**L**KK(Abz)G	≥ 91	1865.9034	1865.9061
	16	Y(NO_2)TESESRGAIY**J**KK(Abz)G	≥ 88	1863.8877	1863.8887
	17	Y(NO_2)TE**G**ESRGAIYYKK(Abz)G	≥ 82	1885.8721	1885.8747

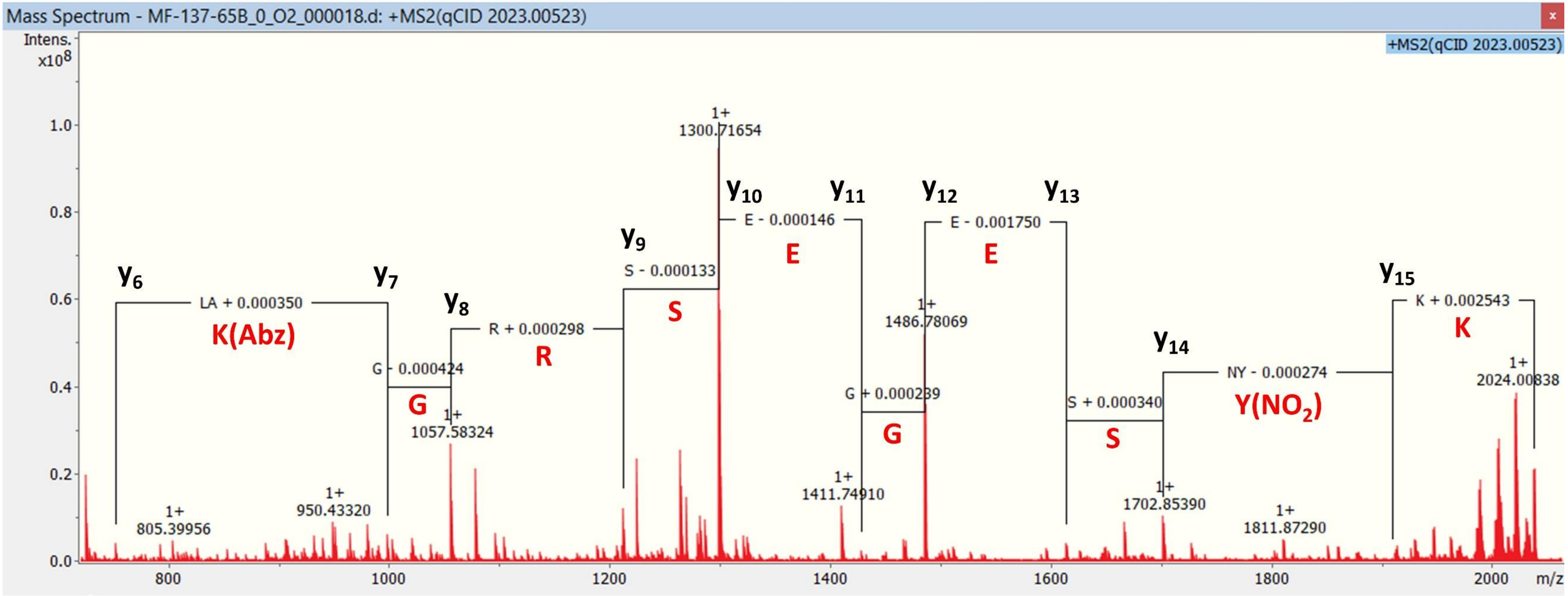

Figure S2. Tandem mass spectrum of the peptide hit KY(NO$_2$)SEGESRGK(Abz)JYFKKG (18) obtained from ADAMTS-5 library screening. Tandem mass spectrum following fragmentation of $[M – O + H]^+$ (2023.00523 u), where M = mass of the quasi-molecular ion. For clarity, only selected residue losses are shown and only fragment ion peaks that could be identified as b ions or y ions have been labelled. Spectra were analysed and processed using Bruker Compass DataAnalysis software.

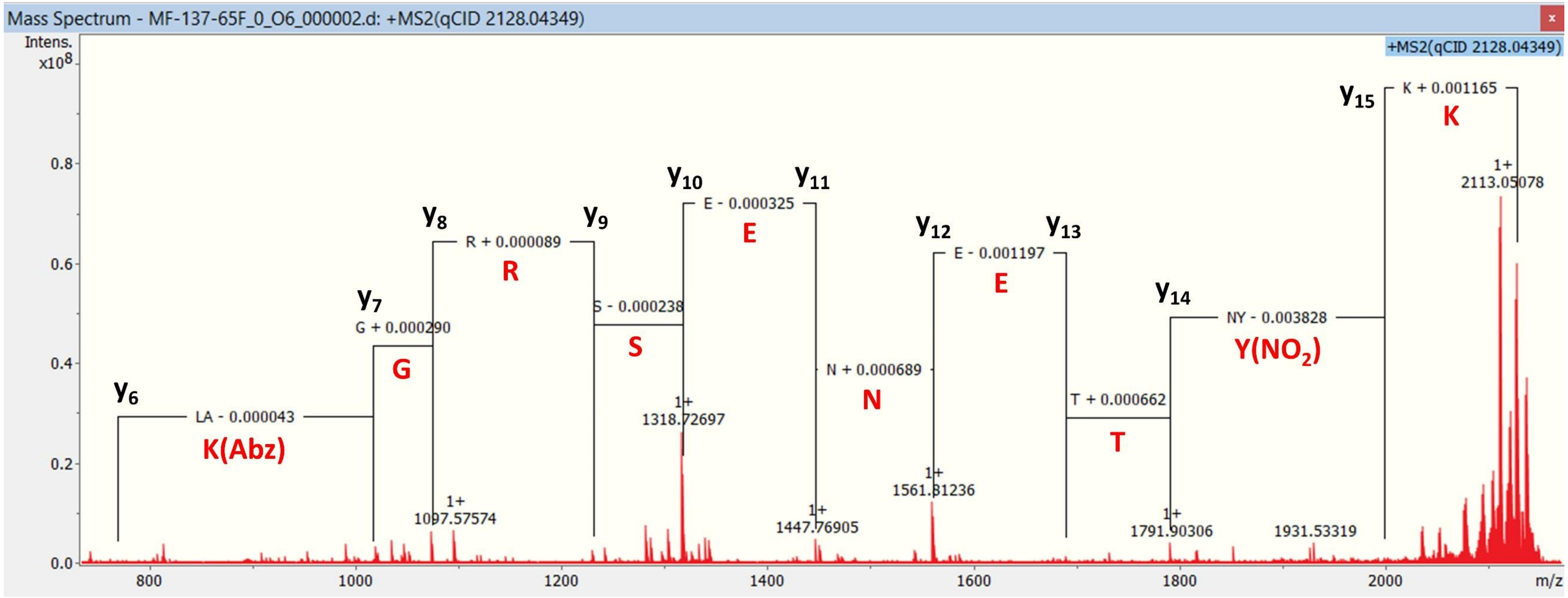

Figure S3. Tandem mass spectrum of the peptide hit KY(NO₂)TENESRGK(Abz)IYYKKG (19) obtained from ADAMTS-5 library screening. Tandem mass spectrum following fragmentation of the quasi-molecular ion $[M + H]^+$ (2128.04349 u), where M = mass of the quasi-molecular ion. For clarity, only selected residue losses are shown and only fragment ion peaks that could be identified as b ions or y ions have been labelled. Spectra were analysed and processed using Bruker Compass DataAnalysis software.

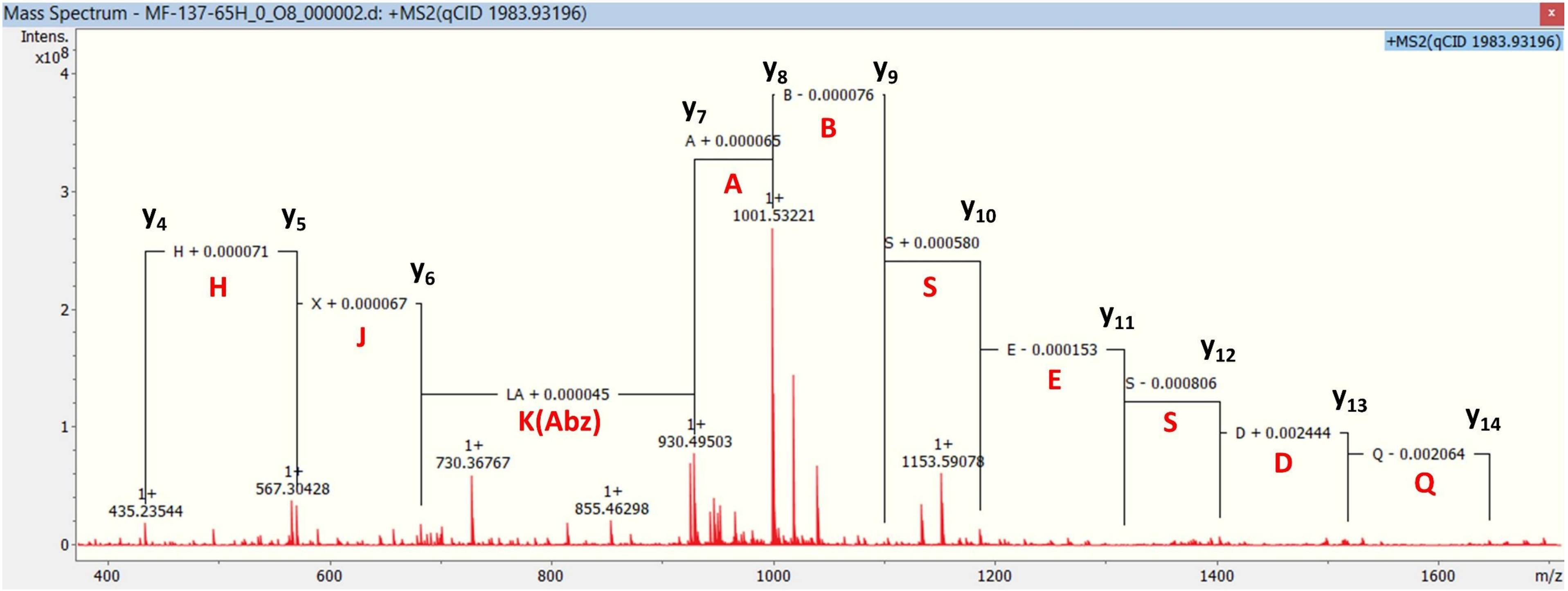

Figure S4. Tandem mass spectrum of the peptide hit KY(NO$_2$)QDSESBAK(Abz)JHYUKG (20) obtained from ADAMTS-5 library screening. Tandem mass spectrum following fragmentation of [M – OH_2 + H]$^+$ (1983.93196 u), where M = mass of the quasi-molecular ion. For clarity, only selected residue losses are shown and only fragment ion peaks that could be identified as b ions or y ions have been labelled. Spectra were analysed and processed using Bruker Compass DataAnalysis software.

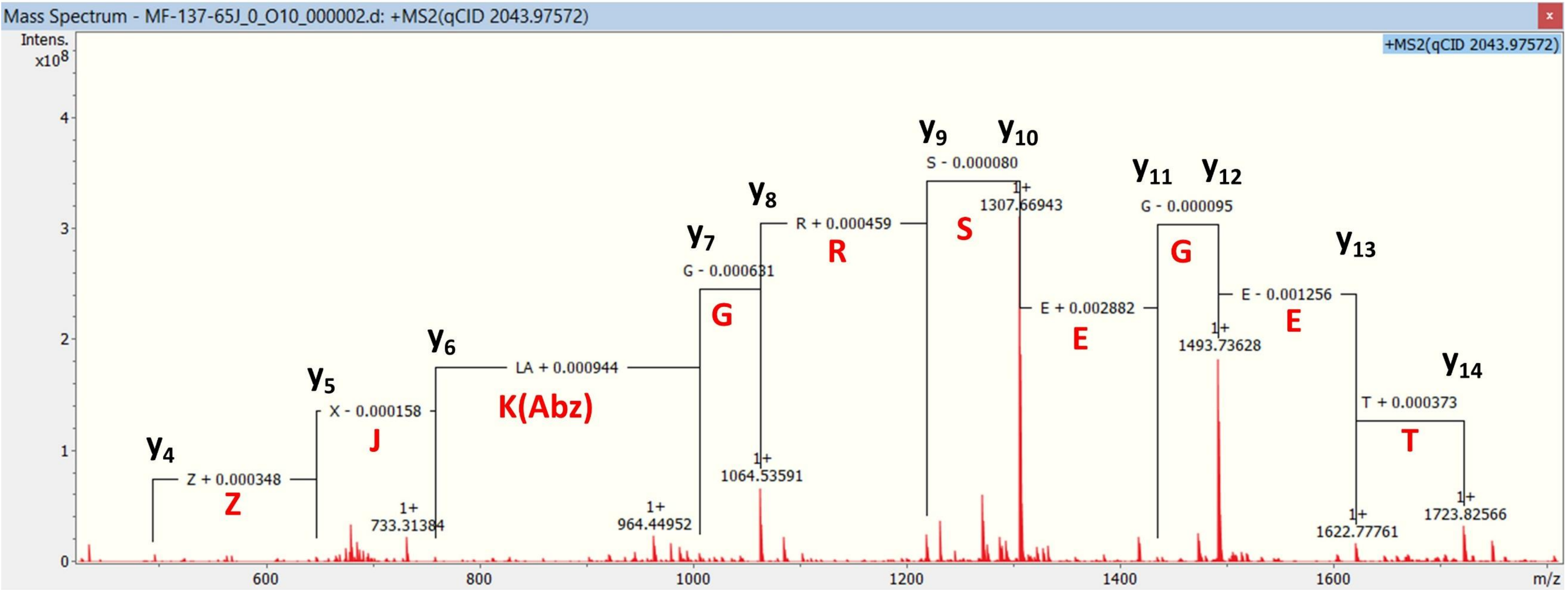

Figure S5. Tandem mass spectrum of the peptide hit KY(NO_2)TEGESRGK(Abz)JZYKKG (21) obtained from ADAMTS-5 library screening. Tandem mass spectrum following fragmentation of $[M – O + H]^+$ (2043.97572 u), where M = mass of the quasi-molecular ion. For clarity, only selected residue losses are shown and only fragment ion peaks that could be identified as b ions or y ions have been labelled. Spectra were analysed and processed using Bruker Compass DataAnalysis software.

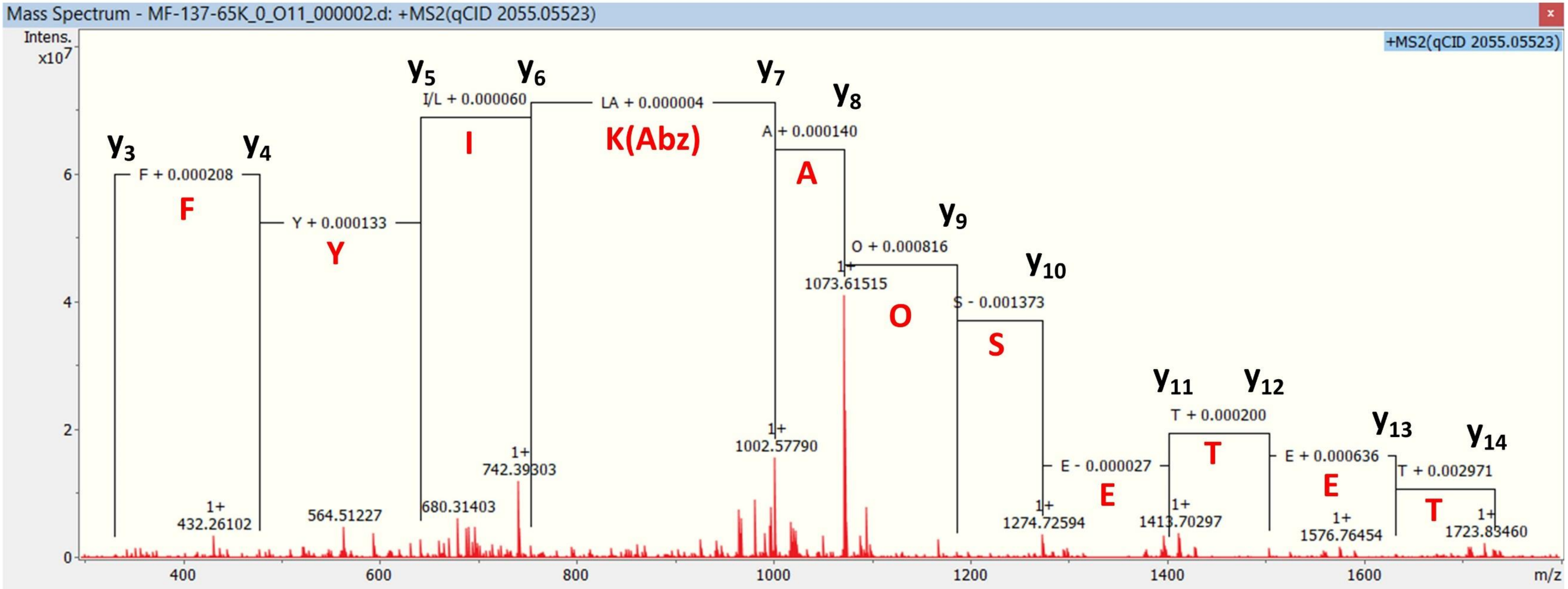

Figure S6. Tandem mass spectrum of the peptide hit KY(NO₂)TETESOAK(Abz)IYFKKG (22) obtained from ADAMTS-5 library screening. Tandem mass spectrum following fragmentation of [M – O + H]$^+$ (2055.05523 u), where M = mass of the quasi-molecular ion. For clarity, only selected residue losses are shown and only fragment ion peaks that could be identified as b ions or y ions have been labelled. Spectra were analysed and processed using Bruker Compass DataAnalysis software.

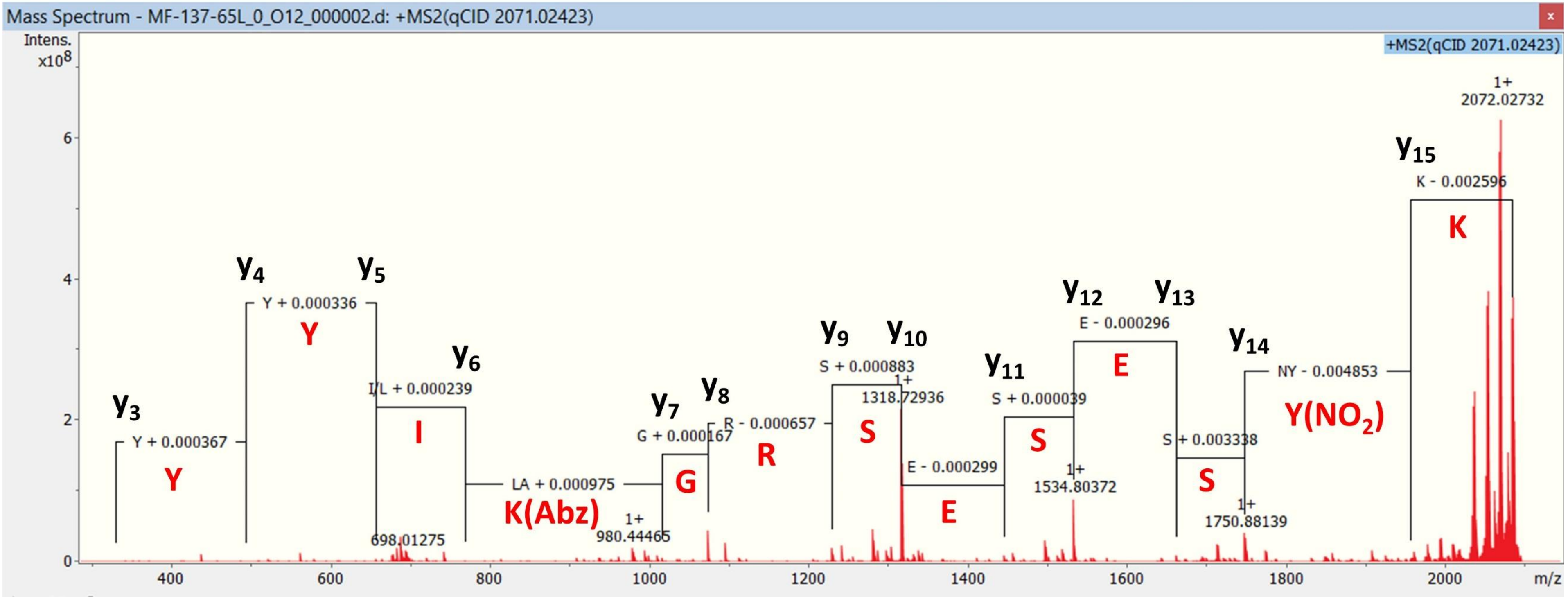

Figure S7. Tandem mass spectrum of the peptide hit KY(NO$_2$)SESESRGK(Abz)IYYKKG (23) obtained from ADAMTS-5 library screening. Tandem mass spectrum following fragmentation of [M – O + H]$^+$ (2071.02423 u), where M = mass of the quasi-molecular ion. For clarity, only selected residue losses are shown and only fragment ion peaks that could be identified as b ions or y ions have been labelled. Spectra were analysed and processed using Bruker Compass DataAnalysis software.

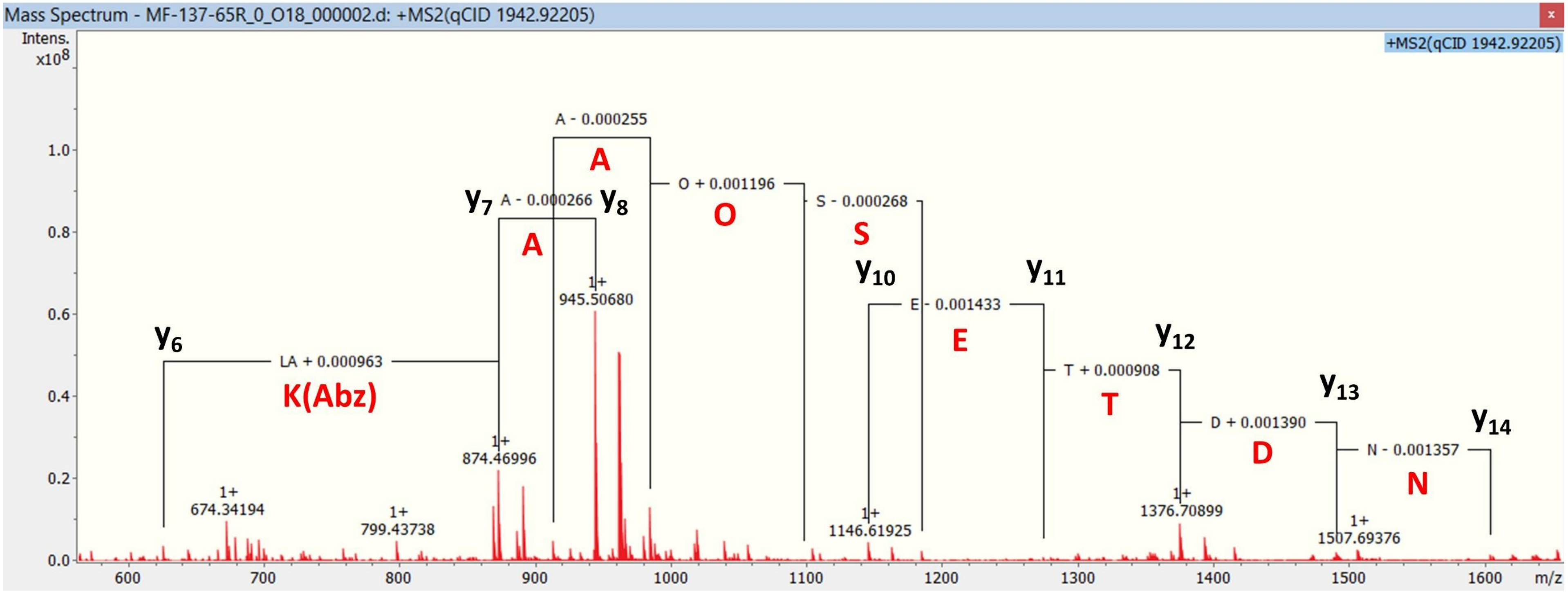

Figure S8. Tandem mass spectrum of the peptide hit KY(NO_2)NDTESOAK(Abz)AHFUKG (24) obtained from ADAMTS-5 library screening. Tandem mass spectrum following fragmentation of $[M – OH + H]^+$(1942.92205 u), where M = mass of the quasi-molecular ion. For clarity, only selected residue losses are shown and only fragment ion peaks that could be identified as b ions or y ions have been labelled. Spectra were analysed and processed using Bruker Compass DataAnalysis software.

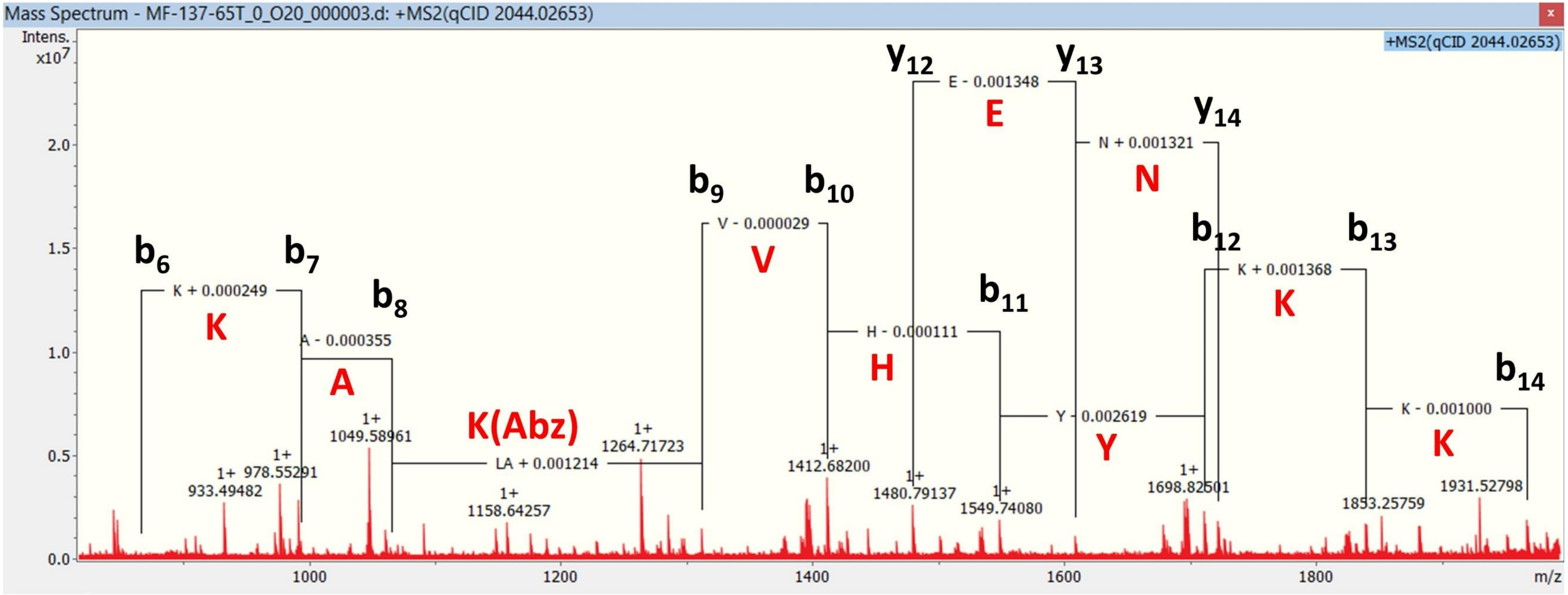

Figure S9. Tandem mass spectrum of the peptide hit KY(NO$_2$)NESESKAK(Abz)VHYKKG (25) obtained from ADAMTS-5 library screening. Tandem mass spectrum following fragmentation of [M – O + H]$^+$ (2044.02653 u), where M = mass of the quasi-molecular ion. For clarity, only selected residue losses are shown and only fragment ion peaks that could be identified as b ions or y ions have been labelled. Spectra were analysed and processed using Bruker Compass DataAnalysis software.

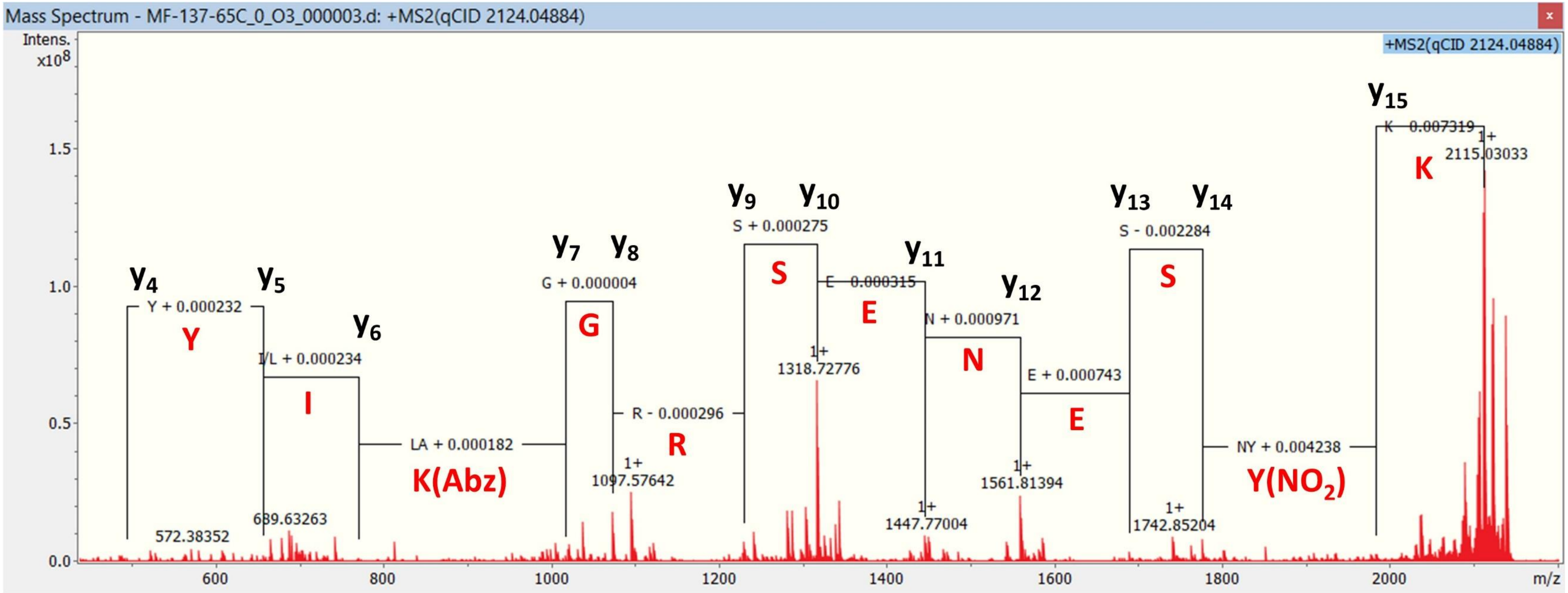

Figure S10. Tandem mass spectrum of the peptide hit KY(NO₂)SENESRGK(Abz)IYYKKG (26) obtained from ADAMTS-5 library screening. Tandem mass spectrum following fragmentation of $[M - O + H]^+$ (2124.04884 u), where M = mass of the quasi-molecular ion. For clarity, only selected residue losses are shown and only fragment ion peaks that could be identified as b ions or y ions have been labelled. Spectra were analysed and processed using Bruker Compass DataAnalysis software.

HPLC characterisation data of commercially synthesised ADAMTS-5 FRET peptide substrates

Abz-TESESRGAIY-Dpa-KK-NH_2

UV trace

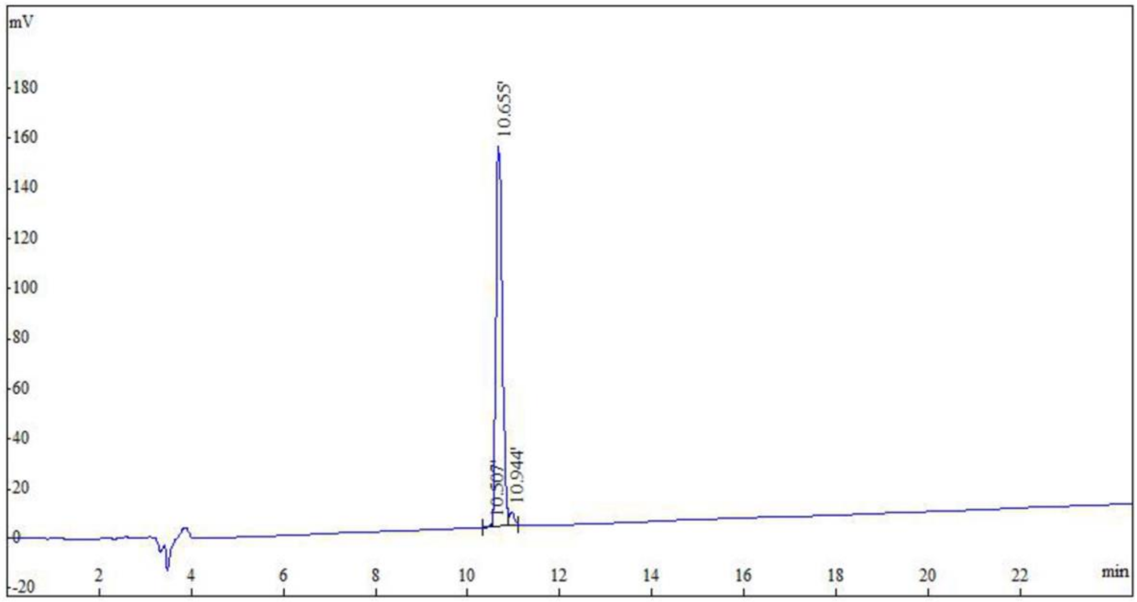

Rank	Time	Conc.	Area	Height
1	10.507	0.6450	8528	1007
2	10.655	96.8862	1281004	151430
3	10.944	2.4688	32642	4835
Total		100	1322174	157272

Mass trace

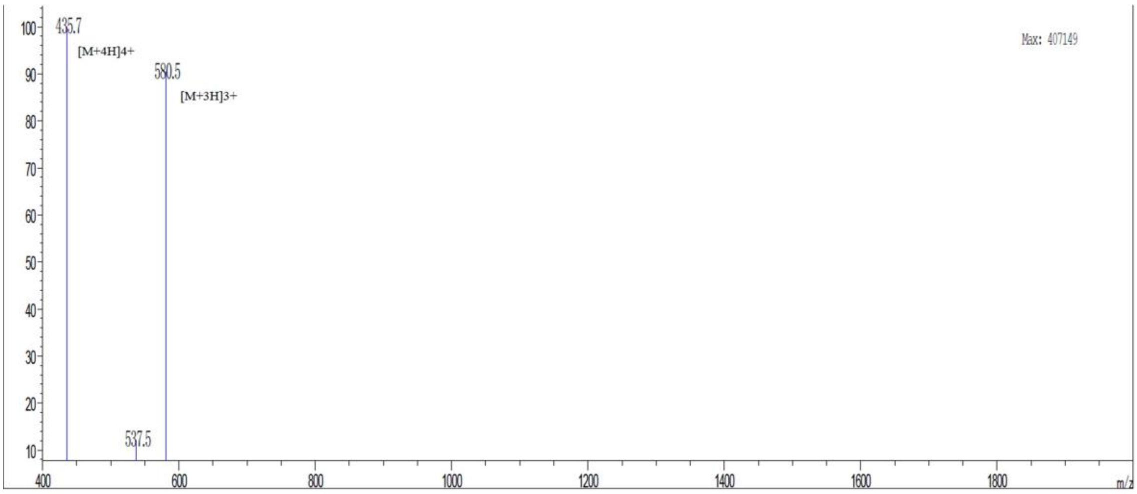

KY(NO$_2$)TESESRGK(Abz)IYYKKG (3)

UV trace

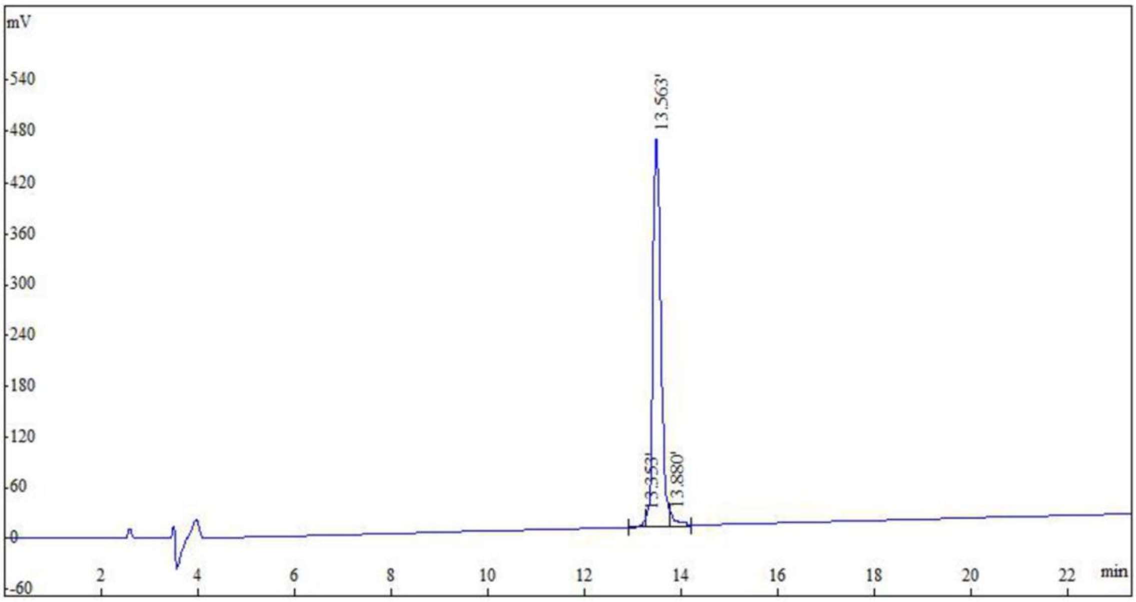

Rank	Time	Conc.	Area	Height
1	13.353	1.1360	55943	11168
2	13.563	96.3088	4742794	457251
3	13.880	2.5552	125833	12116
Total		100	4924570	480535

Mass trace

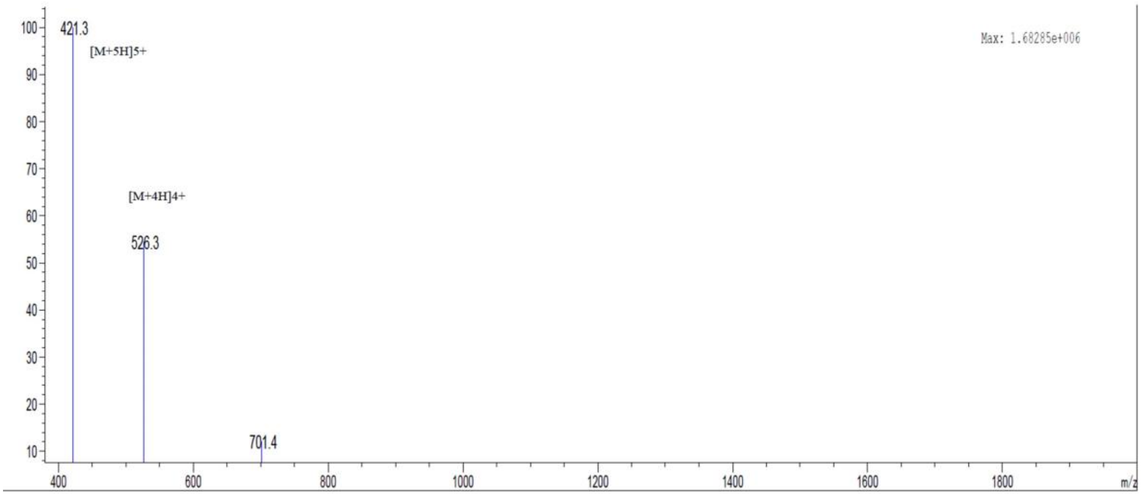

KY(NO$_2$)SEGESRGK(Abz)JYFKKG (18)

UV trace

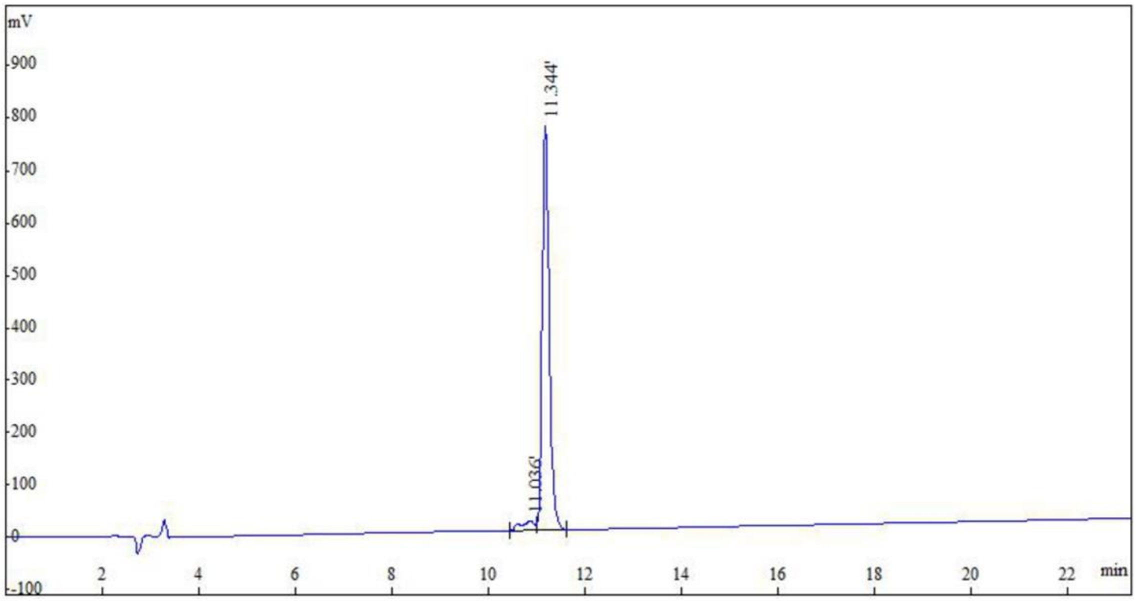

Rank	Time	Conc.	Area	Height
1	11.036	4.0557	306300	17721
2	11.344	95.9443	7245970	766772
Total		100	7552270	784493

Mass trace

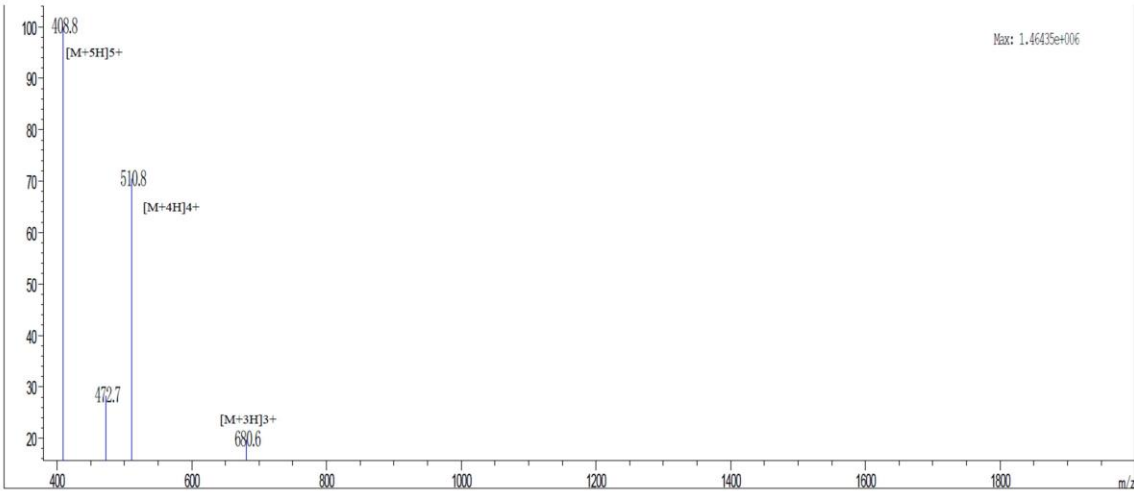

KY(NO_2)TENESRGK(Abz)IYYKKG (19)

UV trace

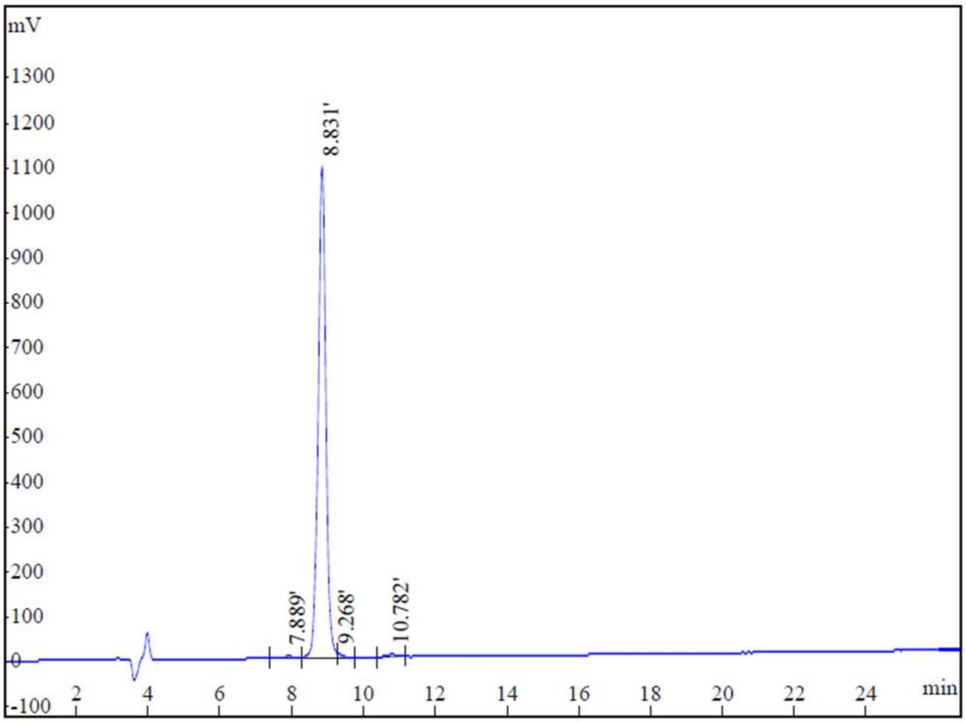

Rank	Time	Conc.	Area	Height
1	7.889	0.4987	75813	4937
2	8.831	98.57	14983773	1090223
3	9.268	0.392	59595	8713
4	10.782	0.5434	82603	5358
Total		100.0000		

Mass trace

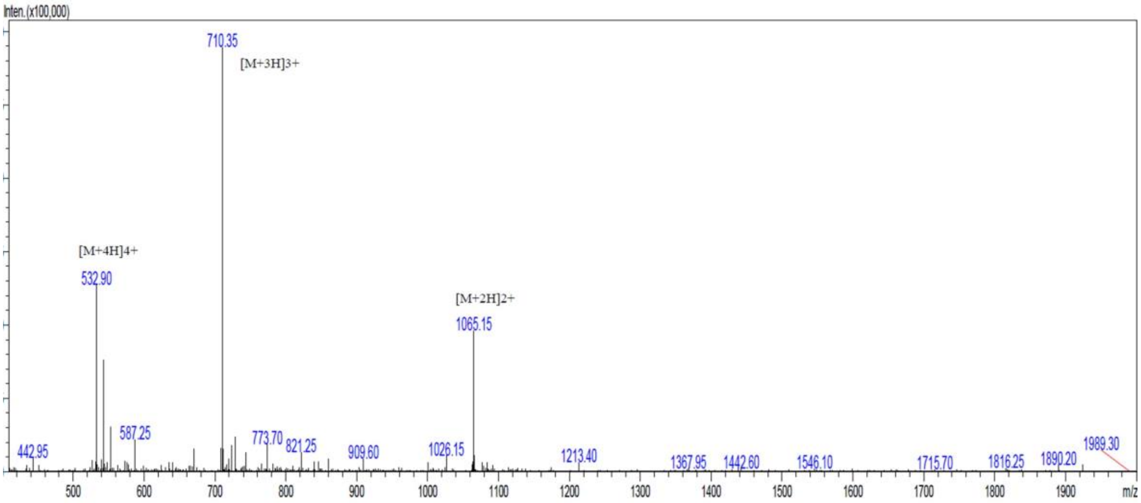

KY(NO_2)QDSESBAK(Abz)JHYUKG (20)

UV trace

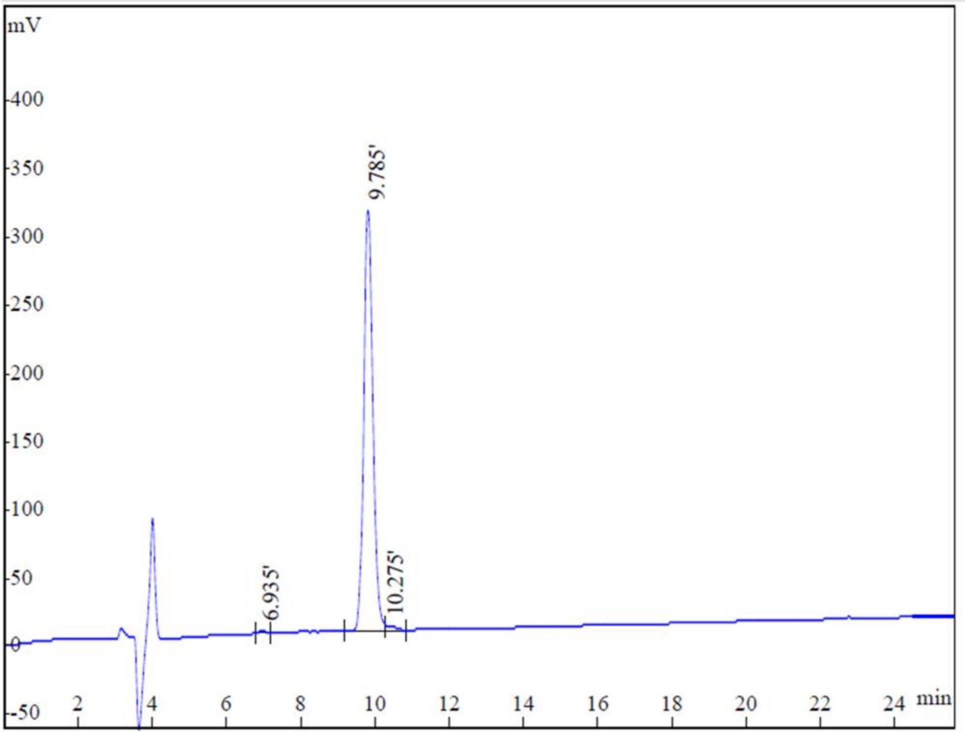

Rank	Time	Conc.	Area	Height
1	6.935	0.4779	23701	2445
2	9.785	98.66	4892636	307866
3	10.275	0.8668	42991	3187
Total		100.0000		

Mass trace

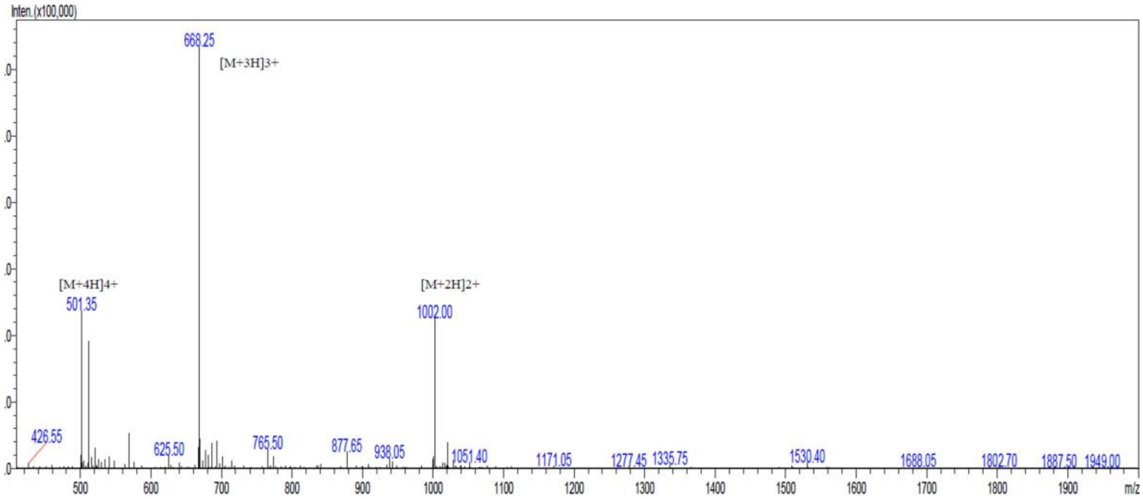

KY(NO_2)TEGESRGK(Abz)JZYKKG (21)

UV trace

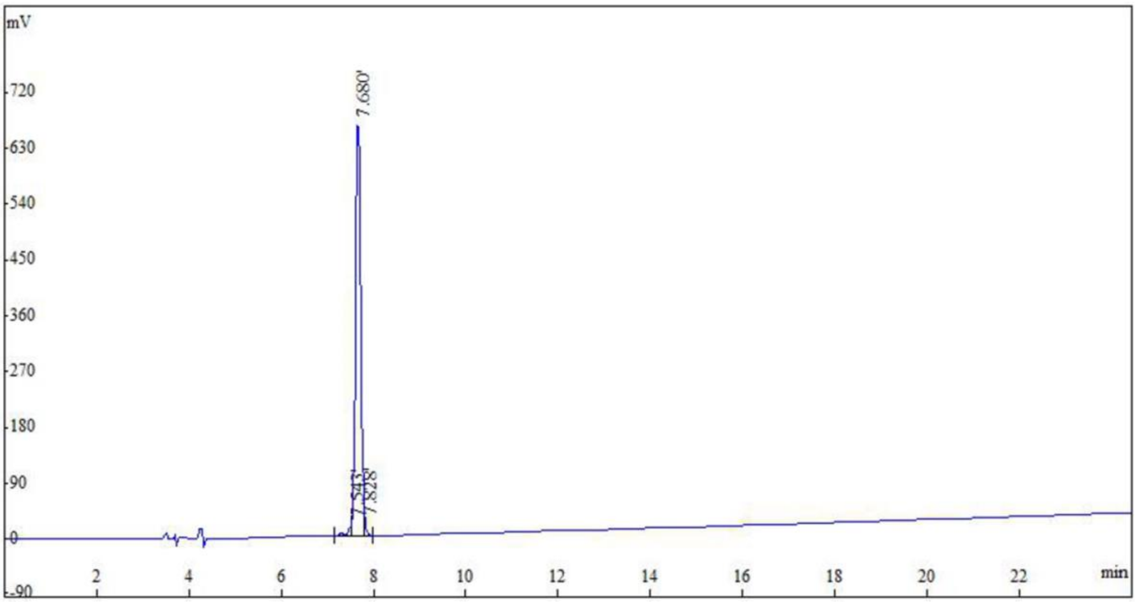

Rank	Time	Conc.	Area	Height
1	7.543	1.8241	87205	17936
2	7.680	97.0825	4641206	662396
3	7.828	1.0934	52272	22738
Total		100	4780683	703070

Mass trace

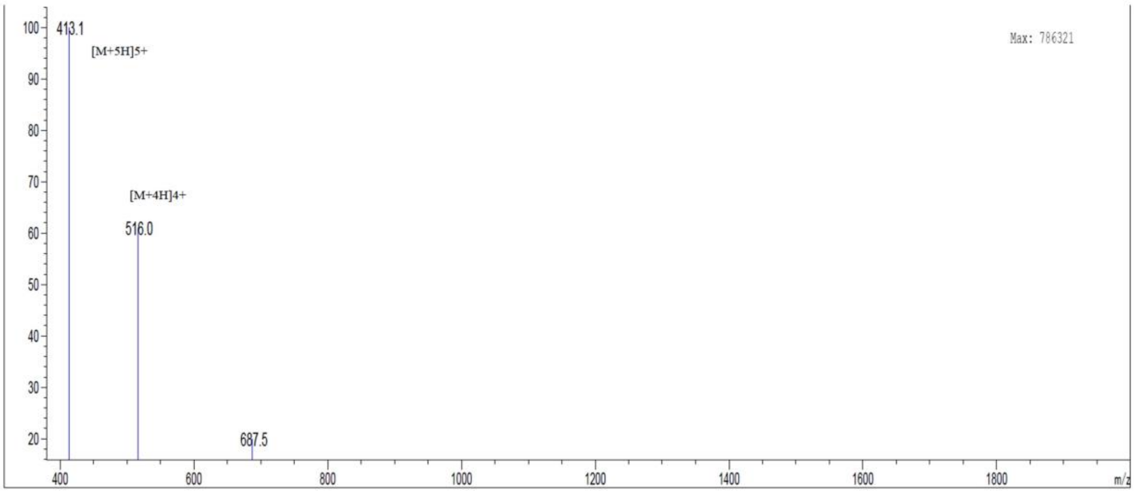

KY(NO_2)TETESOAK(Abz)IYFKKG (22)

UV trace

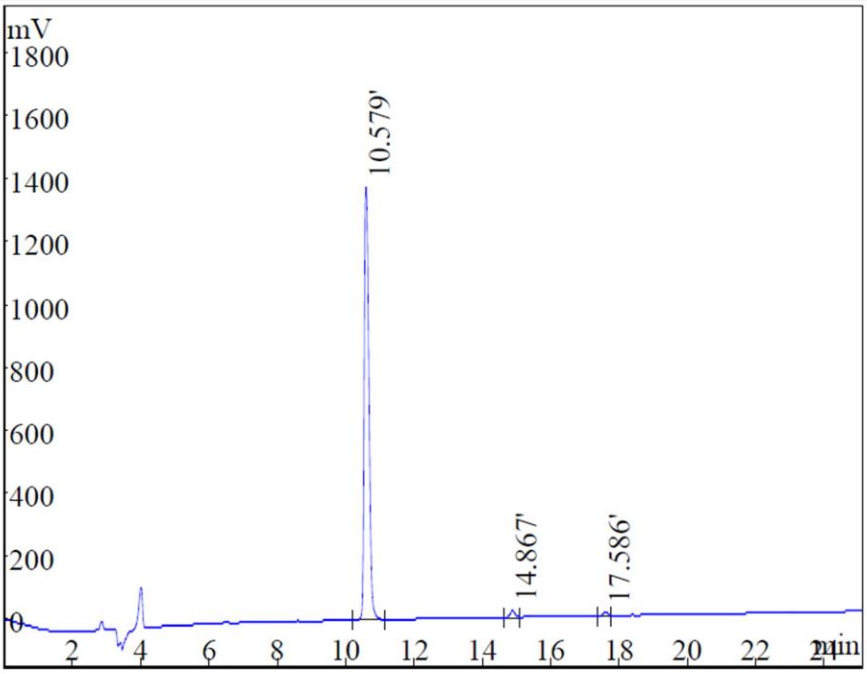

Rank	Time	Conc.	Area	Height
1	10.579	97.83	11728760	1375986
2	14.867	1.413	169350	20453
3	17.586	0.7528	90249	11981
Total		100.0000		

Mass trace

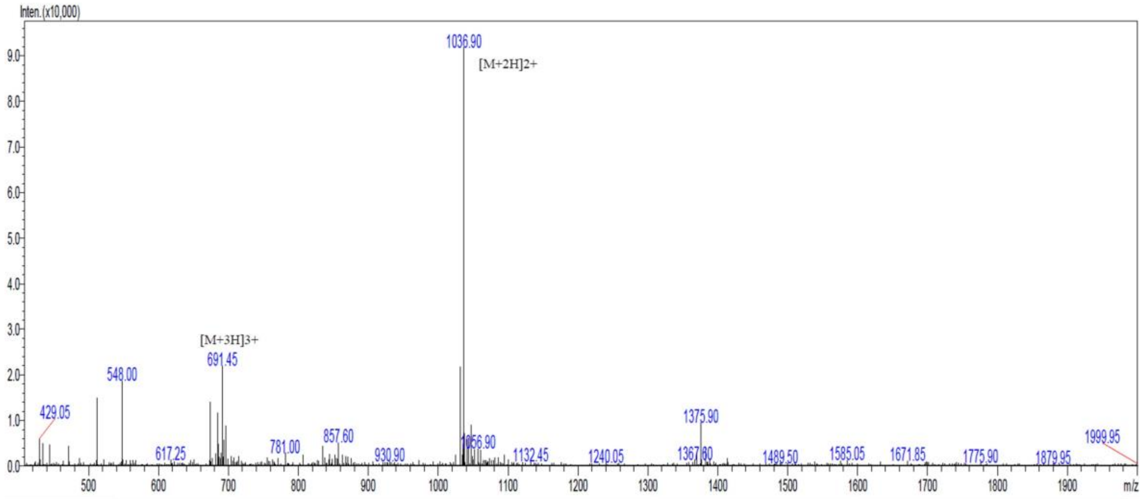

KY(NO_2)SESESRGK(Abz)IYYKKG (23)

UV trace

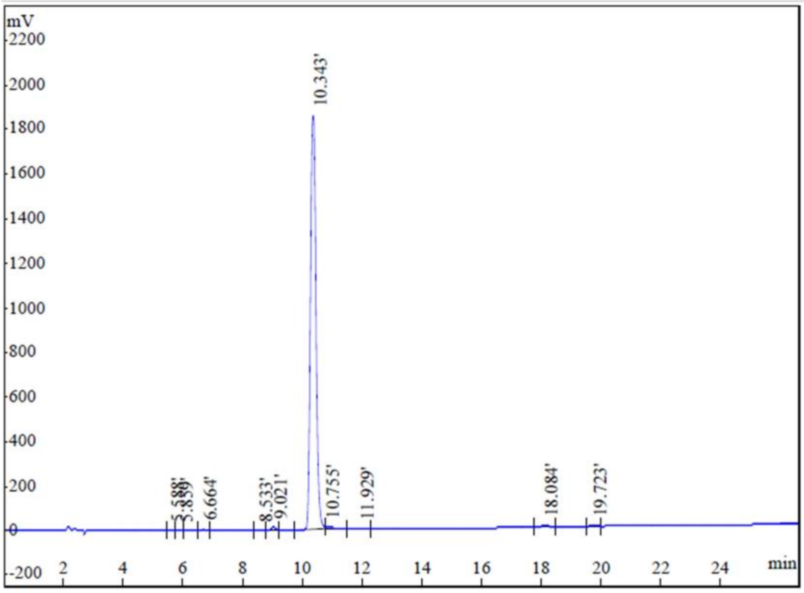

Rank	Time	Conc.	Area	Height
1	5.588	0.1759	38845	5453
2	5.859	0.1707	37709	4824
3	6.664	0.1825	40306	5166
4	8.533	0.06911	15264	2050
5	9.021	0.4412	97436	11531
6	10.343	97.14	21455898	1854737
7	10.755	0.9527	210418	9880
8	11.929	0.1152	25438	2476
9	18.084	0.463	102250	6570
10	19.723	0.287	63393	5551
Total		100.0000		

Mass trace

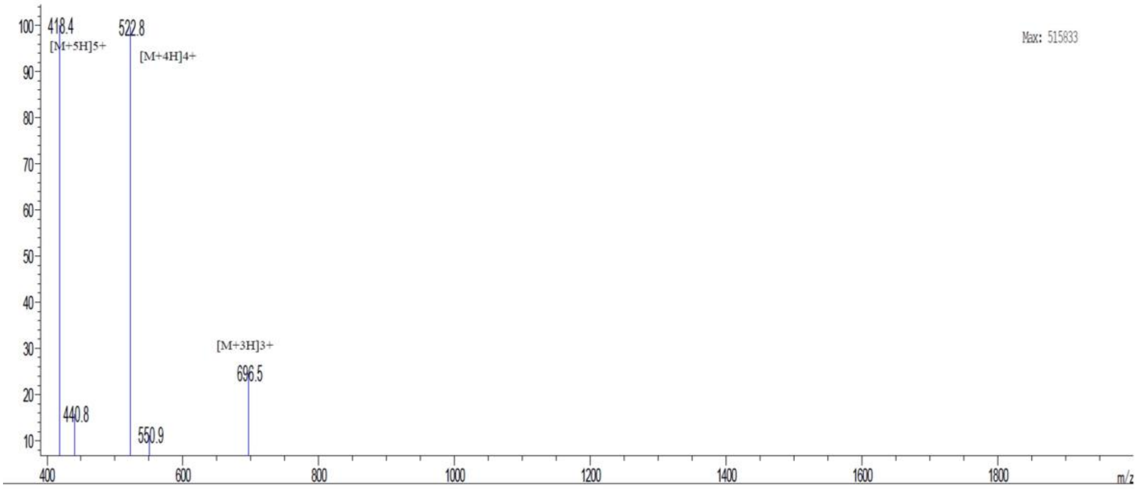

KY(NO_2)NDTESOAK(Abz)AHFUKG (24)

UV trace

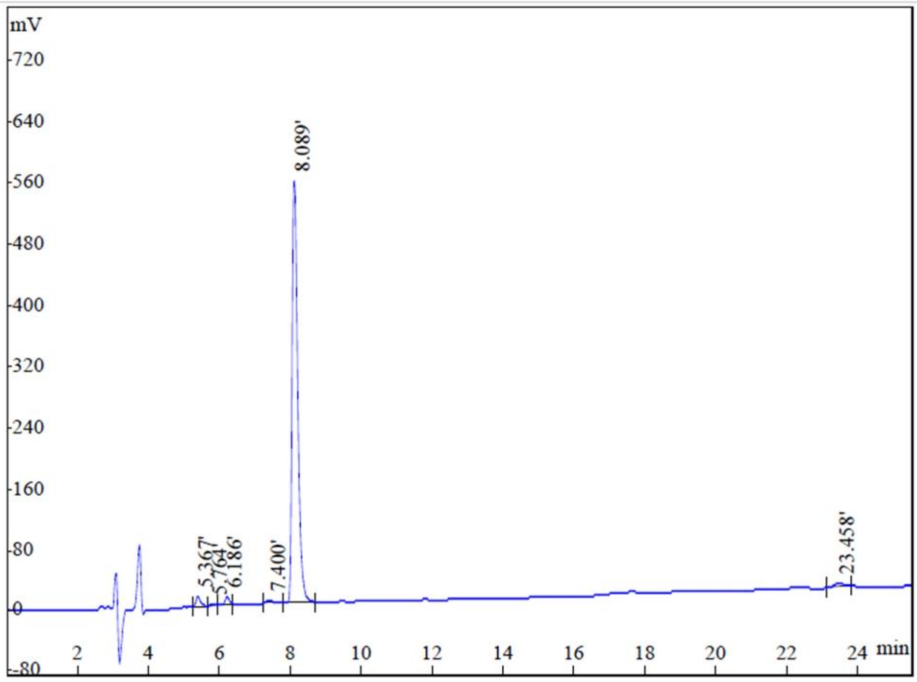

Rank	Time	Conc.	Area	Height
1	5.367	1.563	88791	11881
2	5.764	0.1759	9992	1437
3	6.186	1.264	71813	9589
4	7.400	0.4651	26419	2430
5	8.089	95.12	5402438	550326
6	23.458	1.415	80389	4605
Total		100.0000		

Mass trace

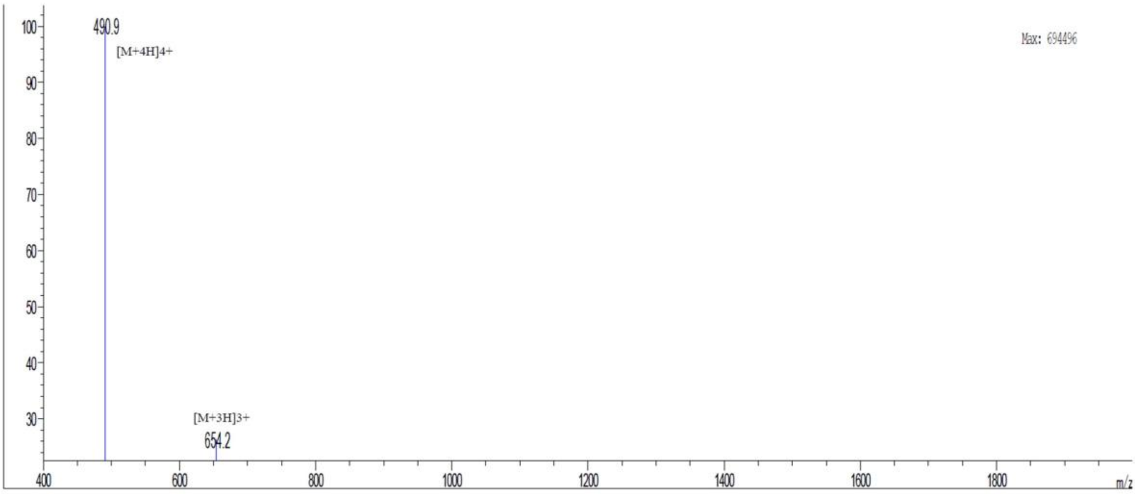

KY(NO_2)NESESKAK(Abz)VHYKKG (25)

UV trace

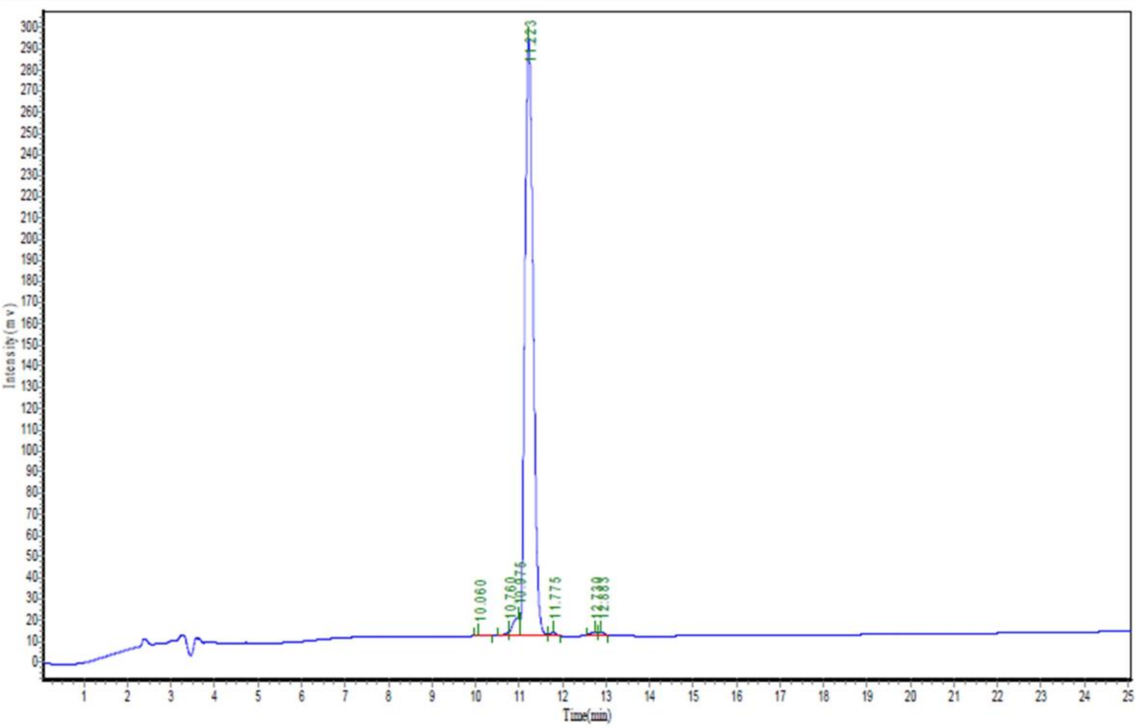

Peak	Time	Height	Area	Conc.
1	10.060	485.004	7658.506	0.1972
2	10.760	1679.758	8693.121	0.2239
3	10.975	8697.797	94104.883	2.4237
4	11.223	282049.031	3729261.500	96.0472
5	11.775	1437.979	15084.591	0.3885
6	12.730	1471.926	14147.716	0.3644
7	12.883	1450.531	13789.783	0.3552
Total				100.000

Mass trace

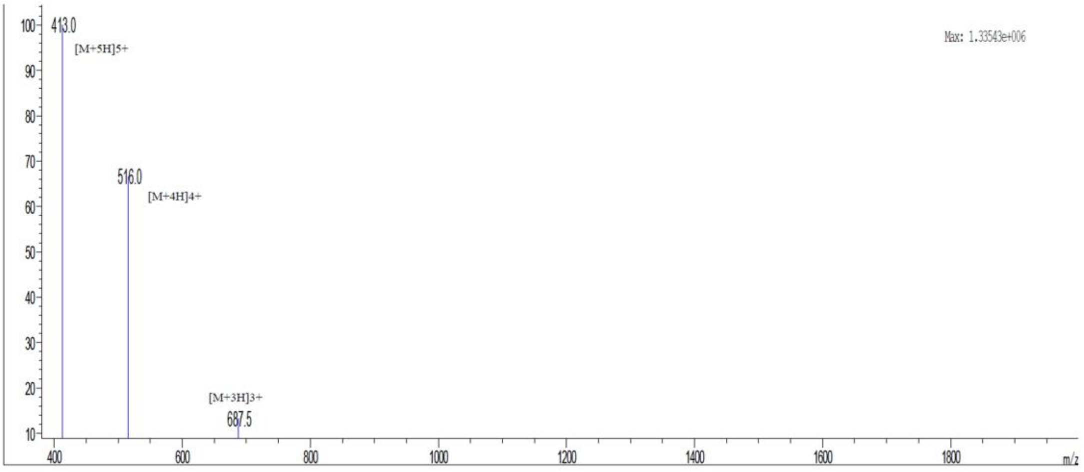

KY(NO_2)SENESRGK(Abz)IYYKKG (26)

UV trace

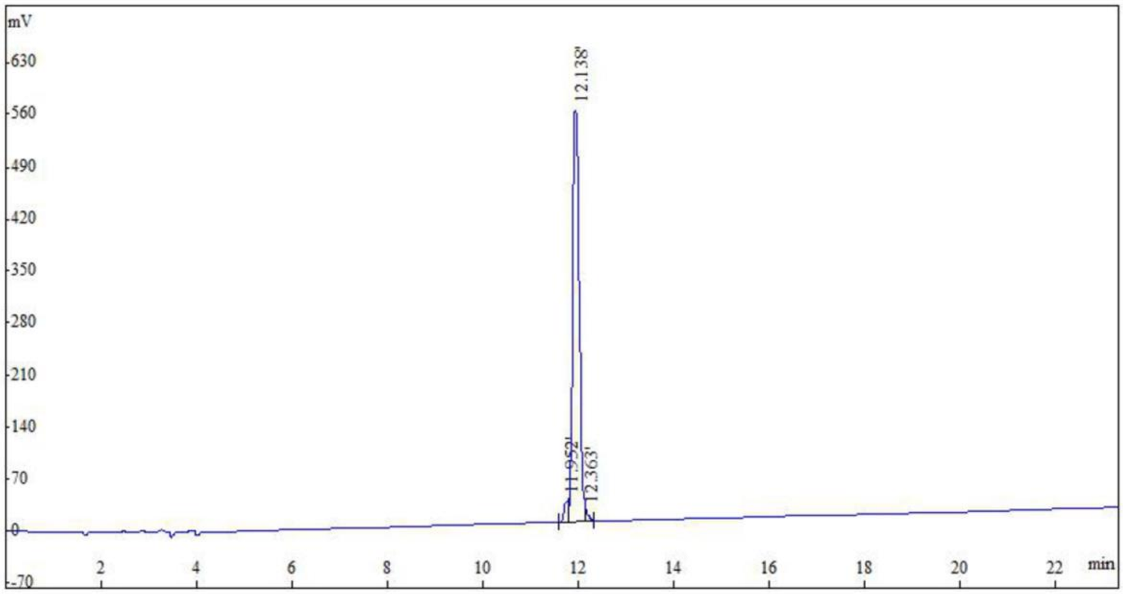

Rank	Time	Conc.	Area	Height
1	11.952	3.5895	180849	27966
2	12.138	95.6302	4818110	551791
3	12.363	0.7803	39312	12293
Total		100	5038271	592050

Mass trace

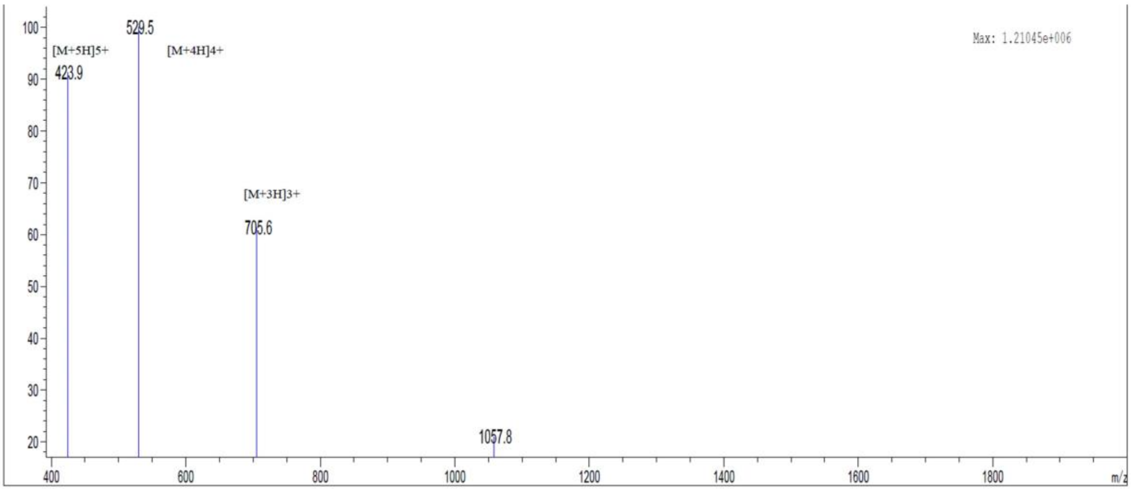

Figures and Tables continued

Table S6. Inner filter effect correction factors for different concentrations of the current substrate

[Substrate], μM	Average Correction factor*
200	0.376
100	0.597
50	0.735
25	0.870
12.5	0.961
6.3	0.943
3.1	1.004
1.6	1.008
0.8	0.981
0	1.000

*n = 3 wells

Table S7. Inner filter effect correction factors for different concentrations of the parent peptide 3

[Substrate], μM	Average Correction factor*
200	0.777
100	0.732
50	0.938
25	1.042
12.5	1.080
6.3	1.060
3.1	1.041
1.6	1.013
0.8	0.954
0	1.000

*n = 3 wells

Table S8. Inner filter effect correction factors for different concentrations of the hit peptide 19

[Substrate], μM	Average Correction factor*
160	0.672
80	0.681
40	0.797
20	0.842
10	0.905
5	0.933
2.5	0.931
1.3	0.964
0	1.000

*n = 3 wells

Table S9. Inner filter effect correction factors for different concentrations of the hit peptide 23

[Substrate], μM	Average Correction factor*
100	0.817
50	0.983
25	1.049
12.5	1.009
6.3	1.066
3.1	1.054
1.6	1.057
0.8	1.083
0	1.000

*n = 3 wells

Table S10. Inner filter effect correction factors for different concentrations of the hit peptide 26

[Substrate], μM	Average Correction factor*
200	0.452
100	0.643
50	0.789
25	0.883
12.5	0.902
6.3	0.910
3.1	0.975
1.6	0.961
0.8	0.983
0	1.000

*n = 3 wells

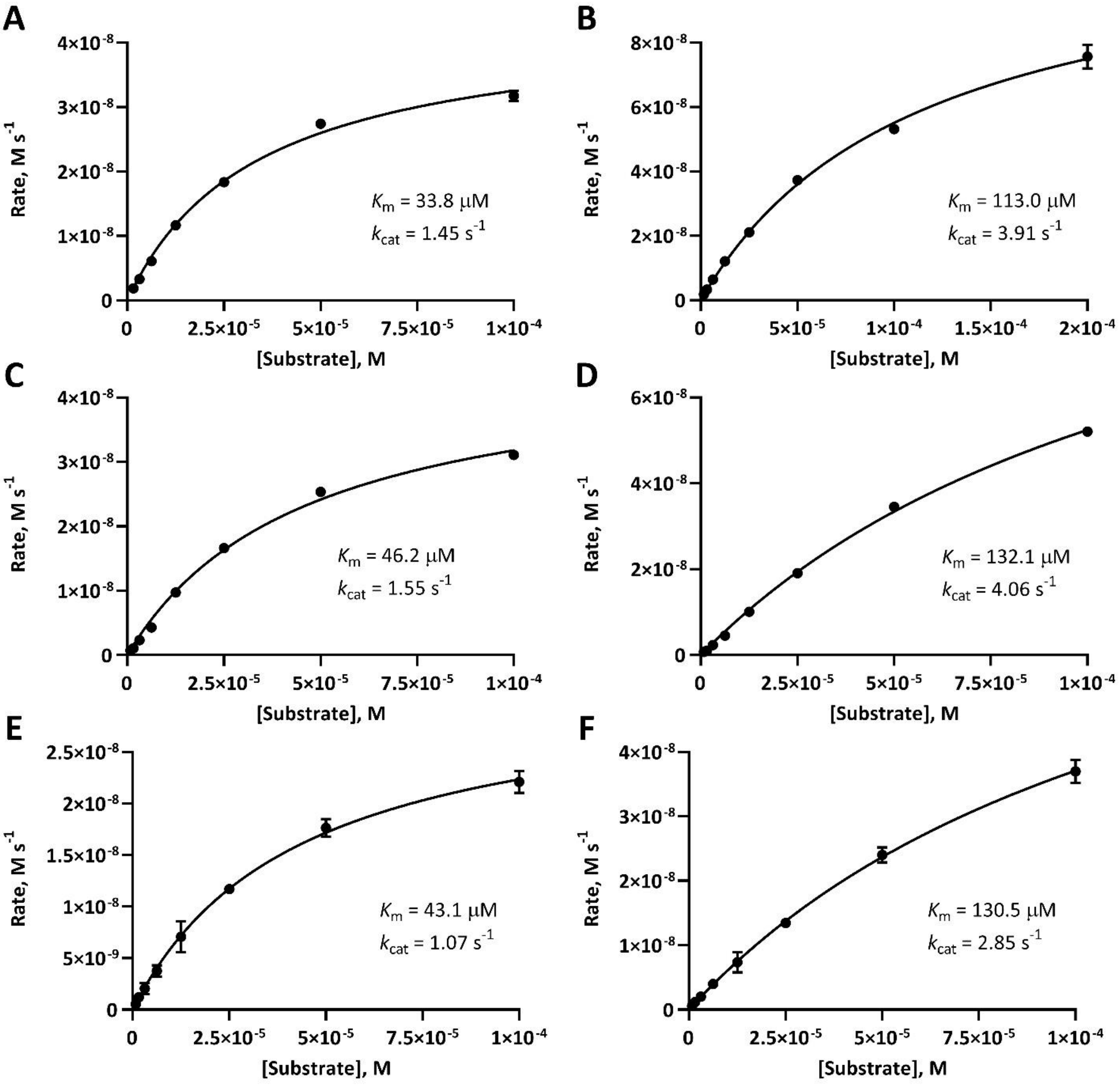

Figure S11. Michaelis-Menten saturation curves for cleavage of the current substrate by ADAMTS-5. Individual curves showing cleavage of Abz-TESE↓SRGAIY-Dpa-KK-NH_2 at different concentrations by a fixed concentration of TS5-5 (30 nM) prior to (*A*, *C*, *E*) and after (*B*, *D*, *F*) correction for the inner-filter effect. The rate at each concentration is expressed in M s^{-1} ± SEM from a single experiment performed in triplicate at 37 °C.

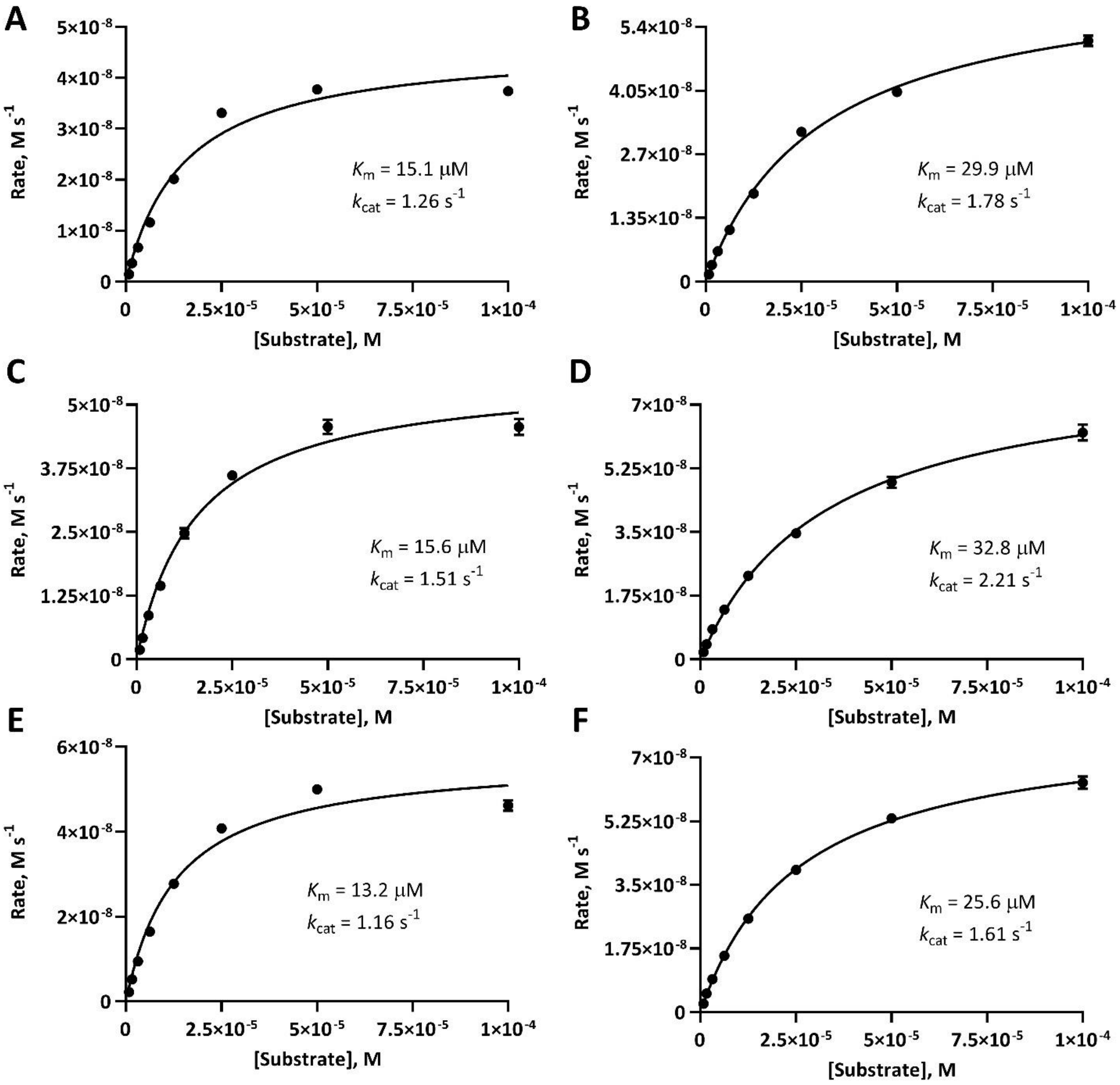

Figure S12. Michaelis-Menten saturation curves for cleavage of parent peptide 3 by ADAMTS-5. Individual curves showing cleavage of KY(NO_2)TESESRGK(Abz)IYYKKG at different concentrations by a fixed concentration of TS5-5 prior to (*A*, *C*, *E*) and after (*B*, *D*, *F*) correction for the inner-filter effect. The rate at each concentration is expressed in M s^{-1} ± SEM from a single experiment performed in triplicate at 37 °C. The concentration of TS5-5 for each experiment was 37 nM (*A*–*D*) or 50 nM (*E*, *F*).

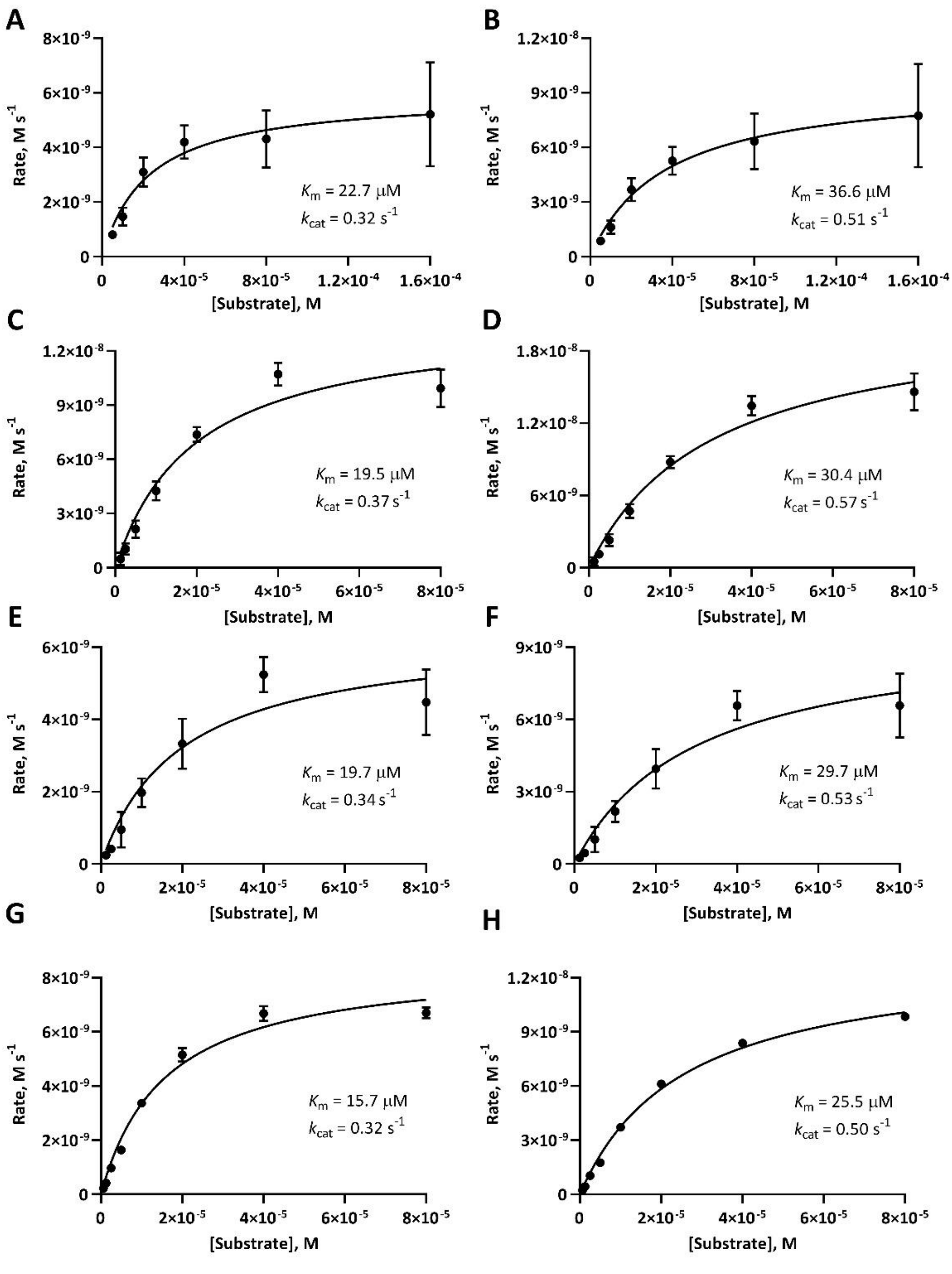

Figure S13. Michaelis-Menten saturation curves for cleavage of hit peptide 19 by ADAMTS-5. Individual curves showing cleavage of KY(NO_2)TENESRGK(Abz)IYYKKG at different concentrations by a fixed concentration of TS5-5 prior to (*A, C, E*, *G*) and after (*B, D, F*, *H*) correction for the inner-filter effect. The rate at each concentration is expressed in M s^{-1} ± SEM from a single experiment performed in triplicate at 37 °C. The concentration of TS5-5 for each experiment was 19 nM (*A*, *B* and *E*, *F*), 37 nM (*C*, *D*) or 27 nM (*G*, *H*).

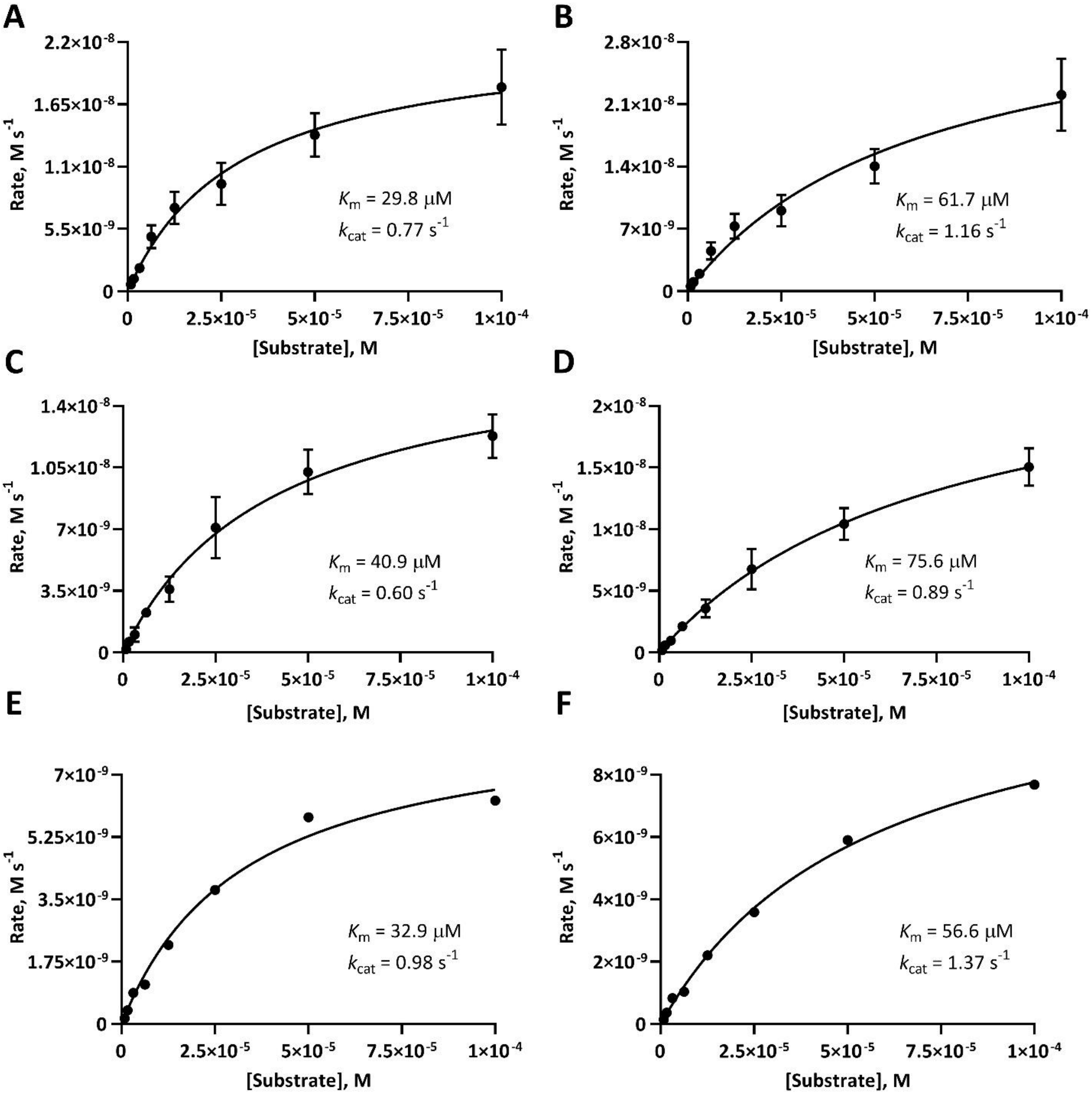

Figure S14. Michaelis-Menten saturation curves for cleavage of hit peptide 23 by ADAMTS-5. Individual curves showing cleavage of KY(NO_2)SESESRGK(Abz)IYYKKG at different concentrations by a fixed concentration of TS5-5 prior to (*A*, *C*, *E*) and after (*B*, *D*, *F*) correction for the inner-filter effect. The rate at each concentration is expressed in M s^{-1} ± SEM from a single experiment performed in triplicate at 37 °C. The concentration of TS5-5 for each experiment was 30 nM (*A*–*D*) or 90 nM (*E, F*).

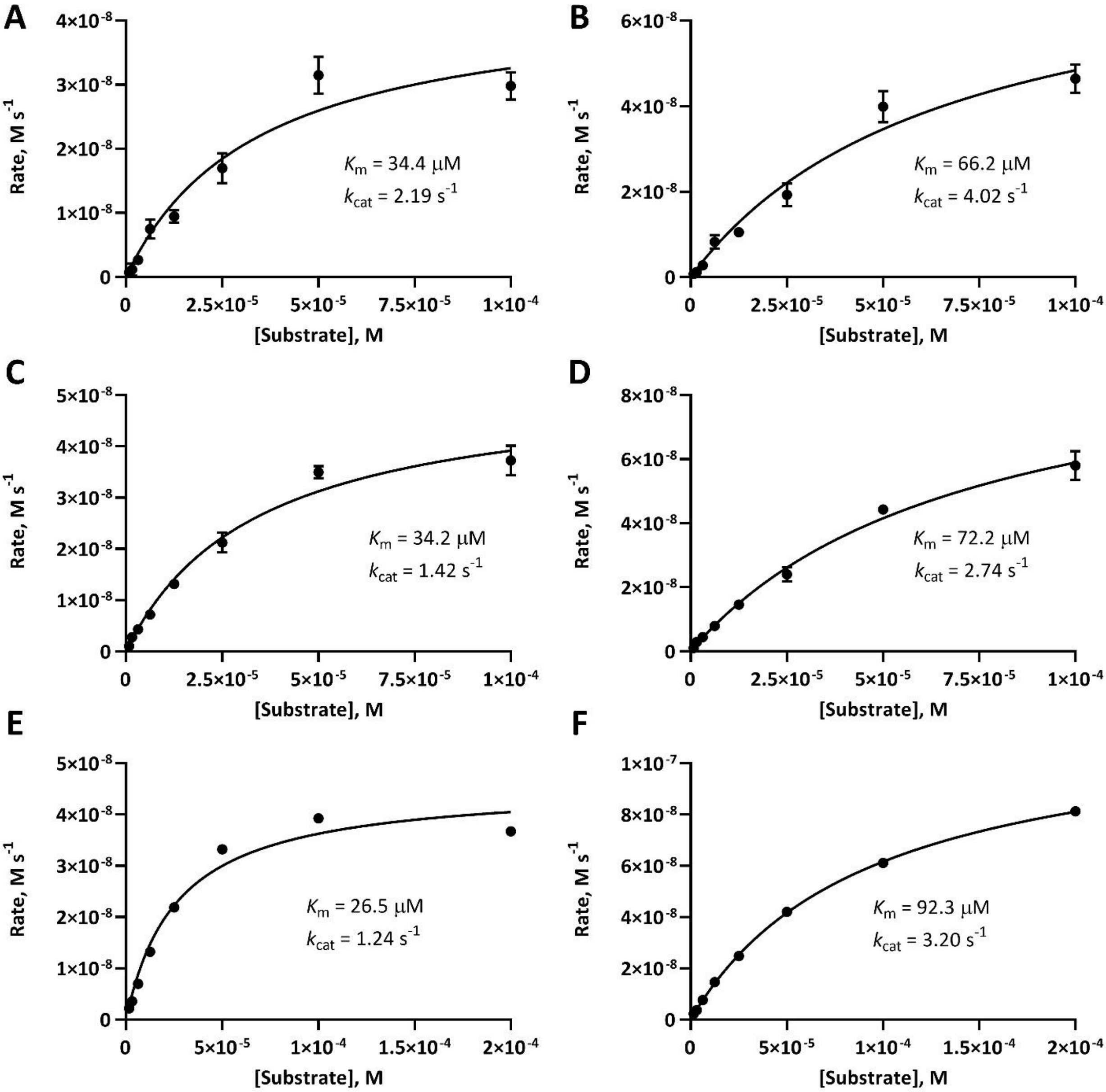

Figure S15. Michaelis-Menten saturation curves for cleavage of hit peptide 26 by ADAMTS-5. Individual curves showing cleavage of KY(NO_2)SENESRGK(Abz)IYYKKG at different concentrations by a fixed concentration of TS5-5 prior to (*A*, *C*, *E*) and after (*B*, *D*, *F*) correction for the inner-filter effect. The rate at each concentration is expressed in M s^{-1} ± SEM from a single experiment performed in triplicate at 37 °C. The concentration of TS5-5 for each experiment was 20 nM (*A*, *B*) or 37 nM (*C*–*F*).

Table S11. Summary of computed *n*-octanol/water partition coefficients for the current ADAMTS-5 substrate, library parent peptide and selected hit peptides.

Peptide	Sequence	cLog*P*
**Current**	Abz-TESE↓SRGAIY-Dpa-KK-NH_2	-2.86 ± 1.11
**3**	KY(NO_2)TESESRGK(Abz)IYYKKG	-3.93 ± 1.13
**19**	KY(NO_2)TENESRGK(Abz)IYYKKG	-4.19 ± 1.14
**23**	KY(NO_2)SESESRGK(Abz)IYYKKG	-4.28 ± 1.13
**26**	KY(NO_2)SENESRGK(Abz)IYYKKG	-4.54 ± 1.14
